# A Nomogram Integrating Ferroptosis- and Immune-Related Biomarkers for Prediction of Overall Survival in Lung Adenocarcinoma

**DOI:** 10.3389/fgene.2021.706814

**Published:** 2021-09-01

**Authors:** Mengyu Chai, Xiuchun Li, Yaxin Zhang, Yemeng Tang, Pingping Shu, Jing Lin, Keqing Shi, Liangxing Wang, Xiaoying Huang

**Affiliations:** ^1^Division of Pulmonary Medicine, Key Laboratory of Heart and Lung, The First Affiliated Hospital of Wenzhou Medical University, Zhejiang, China; ^2^Translational Medicine Laboratory, The First Affiliated Hospital of Wenzhou Medical University, Zhejiang, China

**Keywords:** lung adenocarcinoma, ferroptosis, immune, bioinformatics analysis, nomogram, overall survival

## Abstract

Ferroptosis plays a dual role in cancer, which is known to be affected to antitumor immune responses. However, the association between ferroptosis and antitumor immune responses is uncertain in lung adenocarcinoma (LUAD). In this work, 38 ferroptosis-related genes (FRGs) and 429 immune-related genes (IRGs) were identified as being differentially expressed between tumor and normal samples. Two risk score formulas consisting of seven FRGs and four IRGs, respectively, were developed by Lasso-penalized Cox regression and verified in the GSE13213 dataset. The CIBERSORT algorithm was used to estimate the relative abundance of immune cells in tumors. The correlation between FRGs and immune cells was evaluated using the TIMER database. The results indicated that the development of ferroptosis was synergistic with that of anti-tumor immunity in LUAD. The concordance index and calibration curves showed that the performance of a nomogram that combines clinical staging and risk scores is superior to that of models using a single prognostic factor. In conclusion, ferroptosis might be synergistic with anti-tumor immunity in LUAD. The combined nomogram could reliably predict the probability of overall survival of LUAD patients. These findings may be useful for future investigation of prognostic value and therapeutic potential related to ferroptosis and tumor immunity in LUAD.

## Introduction

Lung cancer is the most common type of cancer and the leading cause of cancer-related death worldwide. An estimated 1.8million deaths from lung cancer occurred in 2020 (Sung et al., 2021). Lung adenocarcinoma (LUAD) is the most prevalent subtype of lung cancer, accounting for about 40% (Ma et al., 2013). Although there are many therapeutic options for LUAD, overall prognosis remains poor (Herbst et al., 2018). Therefore, it is necessary to further explore factors related to survival time that may contribute to the development of more effective treatment methods for lung adenocarcinoma.

Ferroptosis is an iron-dependent cell death modality marked by the oxidative modification of the phospholipid membrane (Stockwell et al., 2017). The effect of ferroptosis on tumor growth is not clear and requires further investigation. On the one hand, ferroptosis inhibits tumor growth in mice (Badgley et al., 2020). For example, although pancreatic cancer cells are prone to resisting chemotherapy, they are highly sensitive to artemisinin-induced ferroptosis (Efferth, 2017). On the other hand, ferroptosis can promote tumor growth (Dai et al., 2020). Therefore, examining the association between ferroptosis marker genes and survival may increase understanding of the role played by ferroptosis in LUAD.

Moreover, there seems to be an inextricable link between ferroptosis and immunity. Levels of CD8^+^ and CD4^+^ T cells cannot increase if they lack glutathione peroxidase 4 (GPX4), a key regulator of ferroptosis (Matsushita et al., 2015). Induction of ferroptosis is related to release of PGE2 (Yang et al., 2014), which attenuates antitumor immunity by affecting cDC1s and NK cells (Wang and DuBois, 2015). The synergistic effect of ferroptosis and immune regulation might not only inhibit the primary tumor but also stimulate immune responses in combination with immune checkpoint blockade (Li and Rong, 2020). In a word, FRGs and immunity have a profound impact on each other, and it is necessary to do more research to understand their relationship.

In this work, we constructed two formulas for calculating ferroptosis-related and immune-related risk scores using The Cancer Genome Atlas (TCGA) database. LUAD patients from the Gene Expression Omnibus (GEO) database were used for validation. Some immune cells had the same infiltration trend as risk score increased in two prognostic multigene signatures. In addition, a nomogram combining tumor stage, ferroptosis-related risk score, and immune-related risk score was developed for more accurate prediction of patient survival. Finally, the correlation between ferroptosis-related genes (FRGs) and immune-related genes (IRGs) was assessed.

## Materials and Methods

### Data Acquisition

Gene expression data, including 497 tumor samples and 54 normal samples, in the HTSeq-FPKM format were obtained from the TCGA database using the GDC tool.^1^ We also downloaded clinical data at the GDC portal. Meanwhile, the microarray dataset GSE13213 consisting of 117 patients with LUAD was downloaded from the GEO database^2^ as an external validation dataset. Probes were annotated based on annotation files. In total, 123 FRGs and 2,483 IRGs were obtained from the FerrDb database^3^ and ImmPort Resources^4^ (Supplementary Tables S1, S2), respectively. There was no ethical conflict since all data was obtained from public databases.

### Construction of A Predictive Nomogram

The R/Bioconductor package “limma,” which provides an integrated solution for analyzing gene expression data and is a popular choice for gene discovery through differential expression analyses, was used to identify differentially expressed FRGs and IRGs. The results were visualized in a volcano map using the popular drawing packages “ggplot2” and “ggrepel.” |log2 foldchange|>1 and FDR<0.05 were considered significant. Prognosis-related genes were identified using univariate Cox proportional hazards regression analysis (*p*<0.05). “Venn” R package is a common way to compare different datasets and identify and visualize intersections between sets. It was used to identify intersections between differentially expressed genes (DEGs) and prognosis-related genes as candidates for risk scoring.

To reduce the risk of overfitting, we applied LASSO-Cox regression to construct survival-predicting models. “Glmnet” is the most widely used package for LASSO analysis, and can generate a variety of models, including binary and multinomial logistic regression models, Poisson models, Cox proportional hazards models and SVM models. The risk score was calculated using “glmnet” and “survival” packages. The Cox coefficient and expression levels of the prognosis-related DEGs were extracted to calculate risk scores. The formula was as follows: Risk score=$\overset{N}{\underset{i=1}{\sum }}\left(\mathrm{Coei}*\mathrm{Expi}\right)$, where N represents gene number, Coei represents coefficient value and Expi represents gene expression level. Based on the median risk score, LUAD patients were dichotomized into low- and high-risk groups. Considering the significant batch effect between the TCGA and GEO dataset, we used the median value of each to divide the high and low risk groups. The predictive ability of the model was evaluated using the log-rank test and receiver operating characteristic (ROC) analysis. The t-distributed stochastic neighbor embedding (t-SNE) test was implemented in “Rtsne” package to visualize clustering. Principal component analysis (PCA) was completed using the prcomp function. A nomogram was constructed to predict overall survival (OS) based on the results of multivariate Cox regression. The concordance index (C-index) was calculated to assess the stability of the nomogram by 1,000 bootstrap replicates. The performance of the prognostic nomogram was evaluated by plotting calibration curves.

### Functional Annotation Analysis and Evaluation of Immune Cell Infiltration

In order to identify different pathways between the two groups, DEGs among groups were analyzed using R package “clusterProfiler” for Gene Ontology (GO). The Kyoto Encyclopedia of Genes and Genomes (KEGG) analysis was performed by the same procedure.

CIBERSORT is a gene-based deconvolution algorithm to estimate the abundance of any of 22 human immune cell types. We loaded “e1071” package to execute this algorithm to quantify the distribution of types of infiltrating immune cells in lung adenocarcinoma samples. The Wilcox test was conducted to identify disparities in the infiltration levels of immune cells between different risk groups, including B cells, plasma cells, T cells, natural killer cells, monocytes, macrophages, dendritic cells, mast cells, eosinophils, and neutrophils. Inter-group differences were identified by “limma” package and displayed in Violin plots using “Vioplot” package. The TIMER web server^5^ is a comprehensive resource for analysis of immune infiltrates in multiple cancers. We analyzed the association between gene expression and abundance of infiltrating immune cells using gene modules.

### qRT-PCR Verification

Human bronchial epithelial cells (BEAS-2B) and human LUAD cell lines (PC9 and H1299) were purchased from the American Type Culture Collection (ATCC, United States). They were cultured in RPMI-1640 medium (Gibco, China) or high glucose Dulbecco’s Modified Eagle’s media (DMEM; Hyclone, Logan, UT, United States) supplemented with 10% fetal bovine serum (Gibco, China) at 37°C in an atmosphere of 5% CO_2_.

Total RNA was extracted using the RNeasy mini kit (Qiagen, United States) and reverse-transcribed with the iScript cDNA Synthesis Kit. qRT-PCR was performed using the CFX96 Real-Time System (Bio-Rad, Hercules, CA, United States). All samples were tested in triplicate. Primers were purchased from *Ribobio* (Guangzhou, China) and are listed in Supplementary Table S3.

### Statistical Analysis

The Wilcoxon rank-sum test was used to compare the gene expression in tumor and normal tissues. Risk scores were validated as independent prognostic factors by the application of Cox regression. Pearson correlation analysis was implemented to assess the correlation of gene expression levels. The statistical analysis tool used in this study was R software 4.0.2. All statistical tests were two-tailed. A value of *p*<0.05 was considered statistically significant.

## Results

### Calculation of Risk Scores for FRGs and IRGs

**Figure 1:**
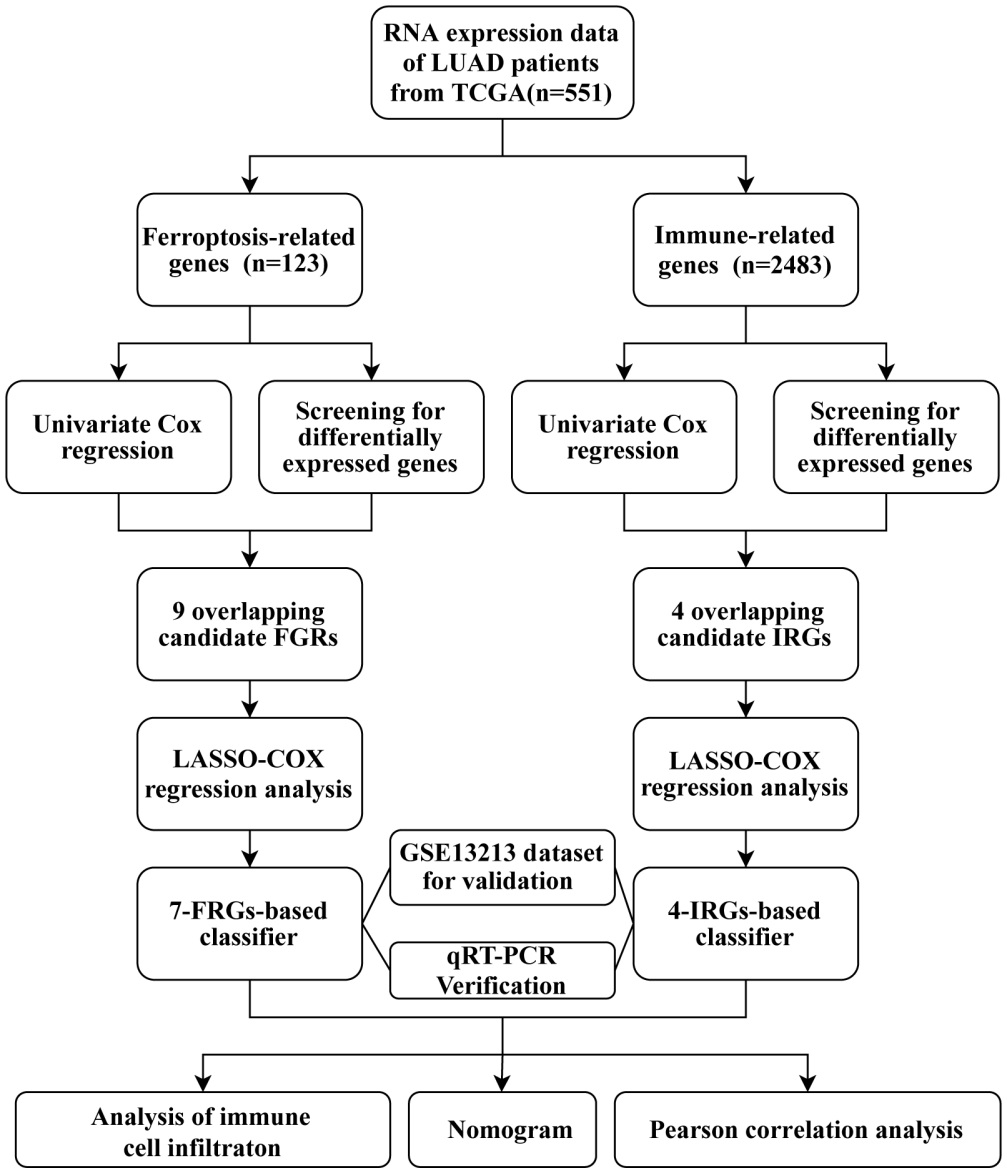
Workflow chart.

**Figure 2:**
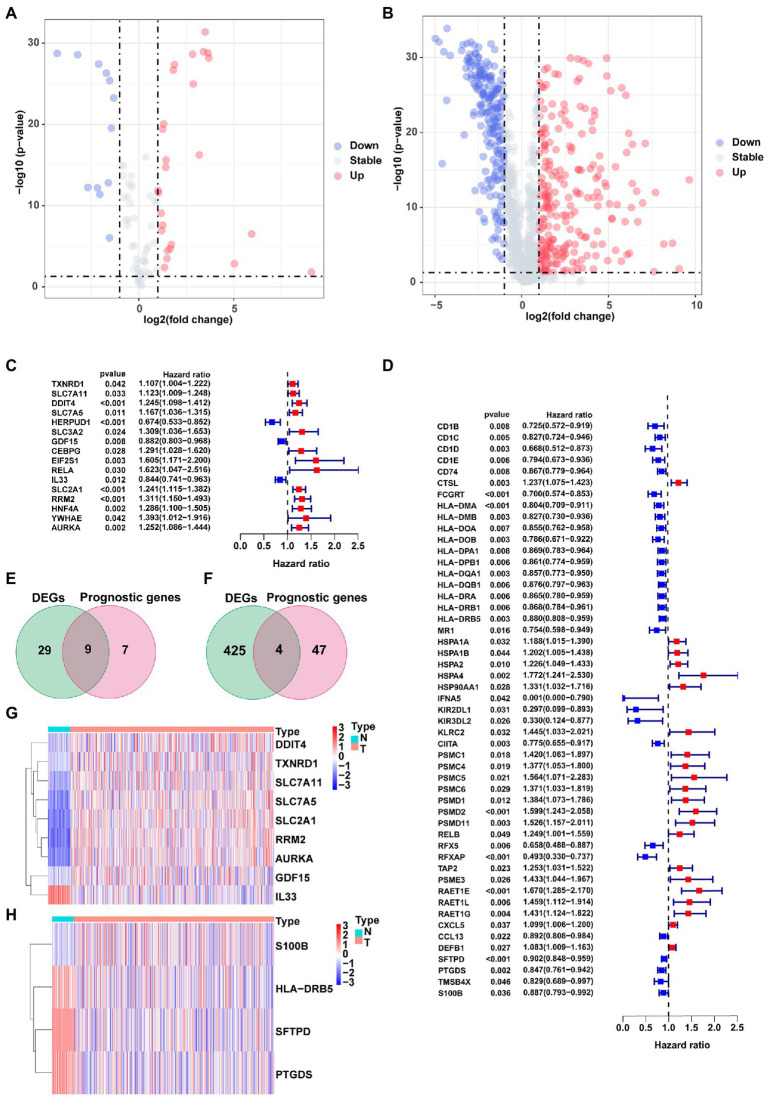
Identification of prognostic ferroptosis-related DEGs and immune-related DEGs. **(A)** Volcano plot of ferroptosis-related DEGs. **(B)** Volcano plot of Immune-related DEGs. **(C)** Forest plot from the univariate Cox proportional hazards regression analysis between FRG expression and survival time. **(D)** Forest plot from the univariate Cox proportional hazards regression analysis between IRG expression and survival time. **(E,F)** Venn diagrams of overlapping candidate genes. **(G,H)** Heatmap plots of overlapping candidate genes.

The study flow chart is shown in Figure 1. Compared with normal samples, 38 significant ferroptosis-related DEGs (26 upregulated and 12 downregulated) and 429 significant immune-related DEGs (224 upregulated and 205 downregulated) were identified (Figures 2A,B; Supplementary Tables S4, S5). By univariate Cox regression analysis, 16 FRGs and 51 IRGs were identified as being related to prognosis (Figures 2C,D; Supplementary Tables S6, S7). Then the intersections of DEGs and prognostic-related genes were identified. Finally, 9 overlapping candidate FGRs and 4 overlapping candidate IRGs were obtained (Figures 2E,F). Heatmaps showed expression of the 13 genes (Figures 2G,H).

**Figure 3:**
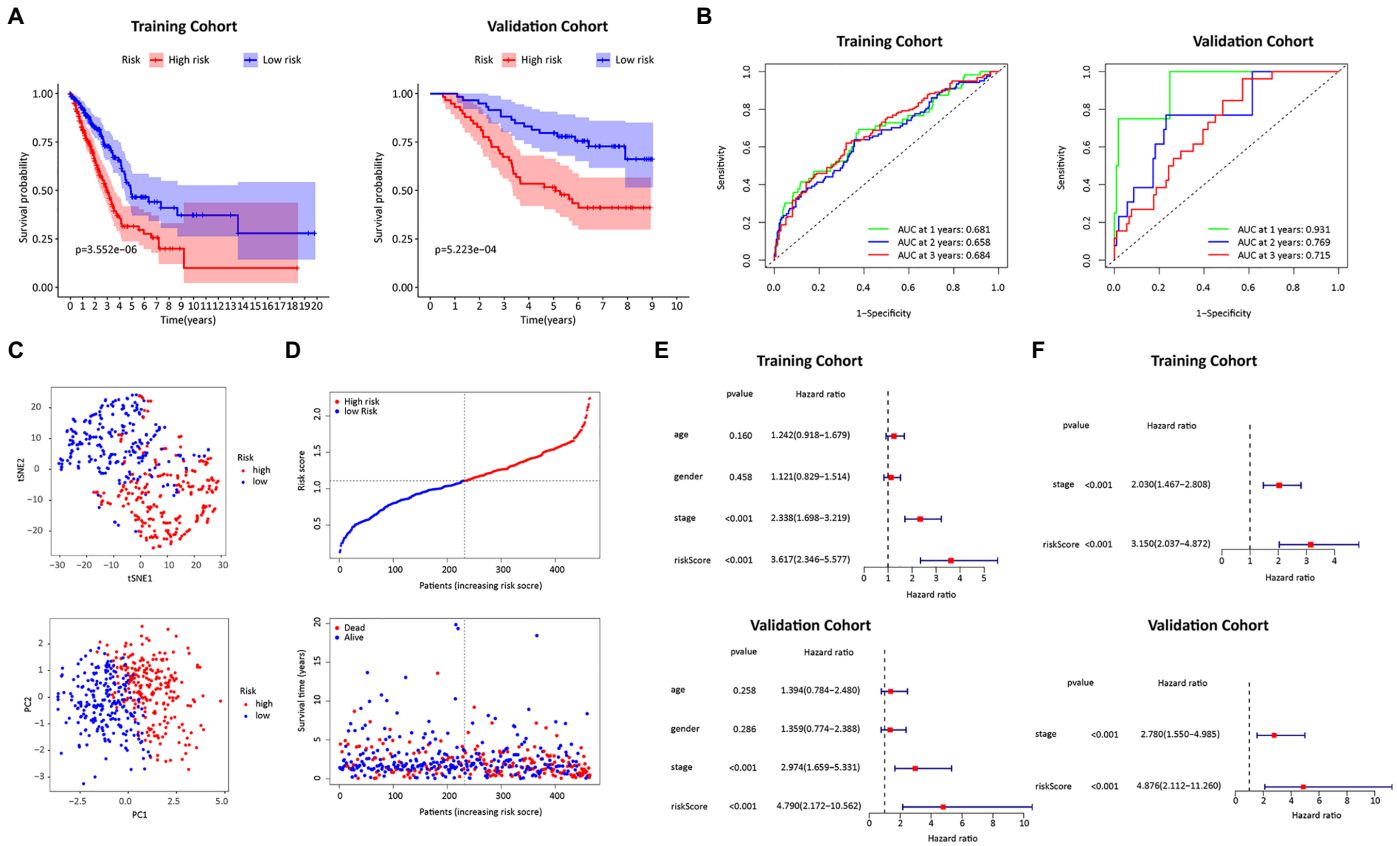
Construction and validation of the FRGs risk score formula. **(A)** Kaplan Meier survival curves for the TCGA training cohort and the GEO validation cohort. **(B)** ROC curves of the FRGs risk score formula in the training and validation cohorts. **(C)** T-SNE analysis and PCA plot of the training cohort. **(D)** The risk and survival status plots in the training cohort. **(E)** Forest plot from the univariate Cox regression analysis in the training and validation cohort. **(F)** The results of multivariate analysis regarding OS in the training and validation cohorts.

We calculated the risk score of FRGs according to the following formula: risk score=(0.048216×SLC7A11)+(0.161996×DDIT4)+(0.004447×SLC7A5)+(−0.068488×GDF15)+(−0.061102×IL33)+(0.027458×SLC2A1)+(0.140388×RRM2). A total of 464 patients were divided into high-risk (*N*=232) and low-risk (*N*=232) groups with the median as the cutoff value. The patients in the low-risk group had significantly higher survival rates than those in the high-risk group (left panel of Figure 3A). The DEGs between the two groups were mainly related to cell cycle transition, mitosis, protein synthesis, and chemotaxis (Supplementary Table S8). The signaling pathways obtained by KEGG analysis were not only correlated with cell division and maturation, but also correlated with immunity (Supplementary Table S9). In the ROC analysis, the AUCs in the first, second and third year were 0.681, 0.658, and 0.684, respectively (left panel of Figure 3B). PCA and t-SNE analysis showed that patients were clustered into distinct groups (Figures 3C,D). In the univariate Cox regression analyses, the risk score was significantly associated with OS in the FRG prognostic risk model (HR=3.617, 95% CI=2.346–5.577, *p*<0.001; upper panel of Figure 3E). Multivariate Cox regression revealed that the classifier was an independent prognostic factor (HR=3.150, 95% CI=2.037–4.872, *p*<0.001; upper panel of Figure 3F). After applying the risk score formula to the GSE13213 dataset, significant differences in OS between the two groups still existed (right panel of Figures 3A,B). Moreover, the risk score remained an independent prognostic factor in the GSE13213 dataset (bottom panel of Figures 3E,F).

**Figure 4:**
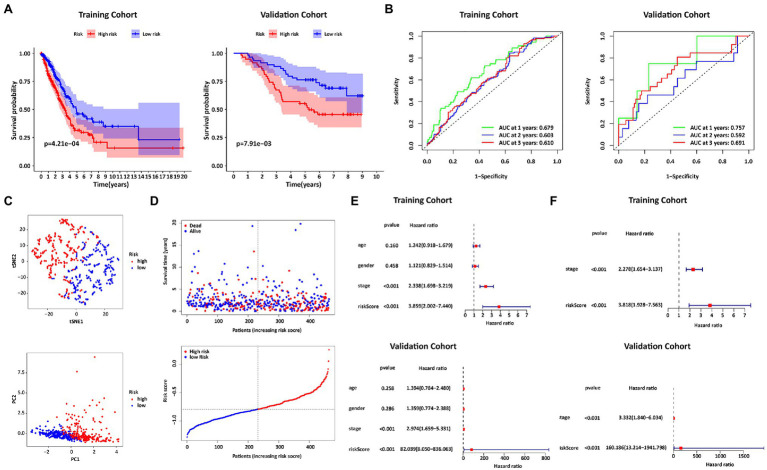
Construction and Validation of the IRGs risk score formula. **(A)** Kaplan Meier survival curves for the TCGA training cohort and the GEO validation cohort. **(B)** ROC curves of the IRGs risk score formula in the training and validation cohorts. **(C)** T-SNE analysis and PCA plot of the training cohort. **(D)** The risk and survival status plots in the training cohort. **(E)** Forest plot from the univariate Cox regression analysis in the training and validation cohort. **(F)** The results of multivariate analysis regarding OS in the training and validation cohorts.

The prognostic risk score of IRGs=(−0.043721×HLA−DRB5)+(−0.055446×SFTPD)+(−0.053414×PTGDS)+(−0.003929×S100B). As shown in the left panel of Figure 4A, patients with high-risk scores tended to have shorter survival times than those with low-risk scores. AUCs in the first, second, and third-year reached 0.679, 0.603, and 0.610, respectively (left panel of Figure 4B). Patients were also distributed in 2 regions (Figures 4C,D). In addition, in the univariate Cox regression analysis, the IRGs prognostic risk score was a variable closely related to prognosis (HR=3.859, 95% CI=2.002–7.440, *p*<0.001; upper panel of Figure 4E). After multivariate Cox regression analysis of multiple factors, the IRGs prognostic risk model remained a reliable independent prognostic factor (HR=3.818, 95% CI=1.928–7.563, *p*<0.001; upper panel of Figure 4F). The validity of the model was also verified in the GSE13213 dataset (right panel of Figures 4A,B; bottom panel of Figures 4E,F).

### Correlation of Immune Cell Infiltration With Risk Scores and Gene Expression

**Figure 5:**
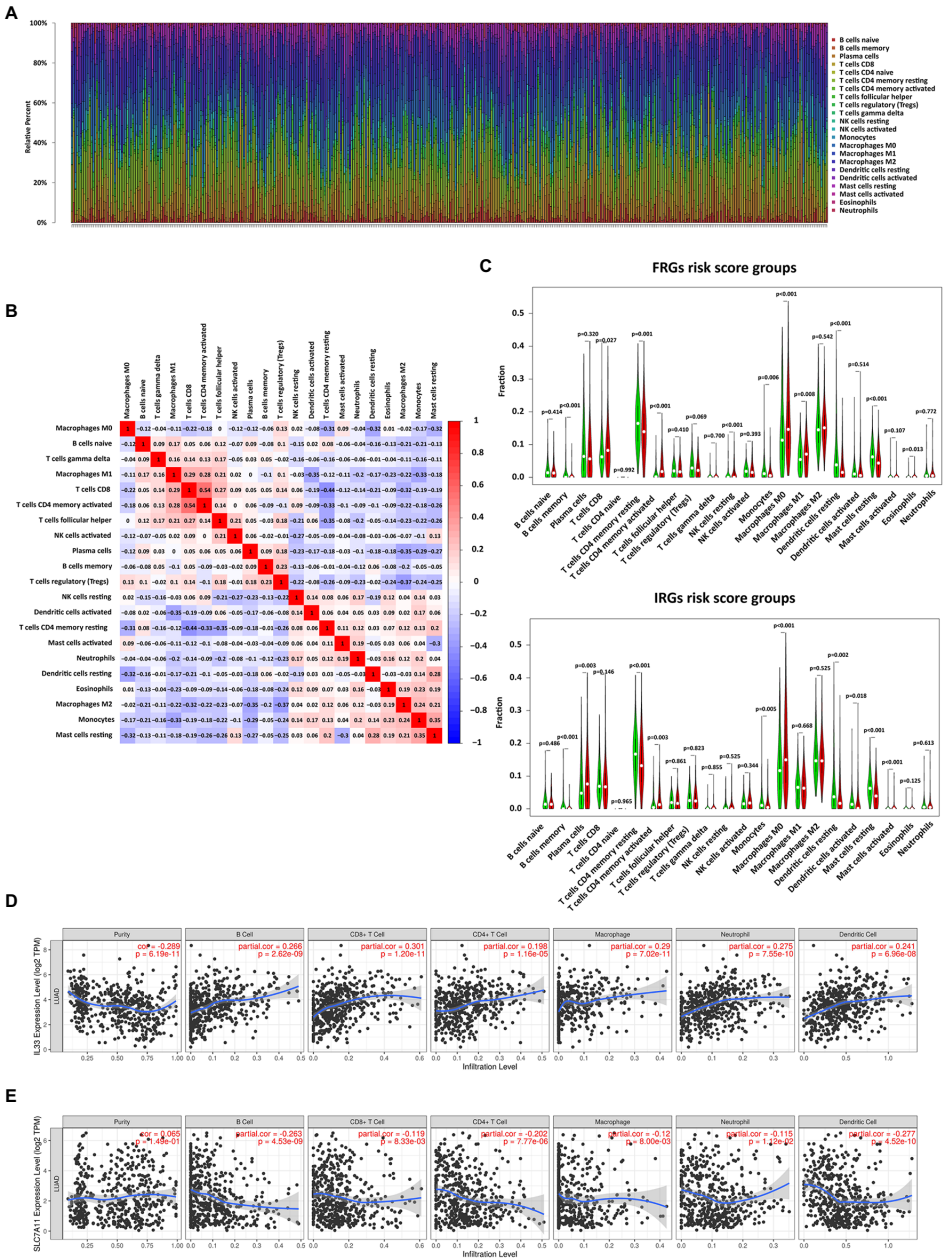
Correlation analysis of immune cell infiltration with risk scores and expression of FRGs. **(A)** The bar chart shows the distribution of 22 types of immune cells in each sample. **(B)** Correlation matrix of immune cell proportions. **(C)** Violin plots of the differentially infiltrated immune cells between the low- and high-risk groups. **(D)** The correlation between IL33 expression and infiltration of immune cell subtypes. **(E)** The correlation between SLC7A11 expression and infiltration of immune cell subtypes.

To better understand the relationship between risk score and immune response, we calculated the proportions of 22 immune cells (Figure 5A). In addition, the correlation matrix of immune cells showed that the infiltration level of activated memory CD8^+^ T cells was highly correlated with CD4^+^ T cells (Figure 5B). In addition, the level of resting mast cells highly correlated with that of monocytes was also high. Subsequently, immune cell infiltration was compared between high and low-risk groups. There were similar trends of differences in infiltration levels for memory B cells, activated memory CD4^+^ T cells, resting memory CD4^+^ T cells, monocytes, M0 macrophages, resting mast cells, and resting dendritic cells in the 2 models (Figure 5C). Next, the relationship between gene expression and immune cell subtype infiltration was further analyzed in the TIMER database (Figures 5D,E). SLC7A11 and IL33 showed a strong correlation with immune cell infiltration. In contrast to SLC7A11, IL33 promoted the infiltration of nearly all types of immune cell. All IRGs were positively correlated with the level of immune cell infiltration (Supplementary Figure S1).

### Construction of the Nomogram

**Figure 6:**
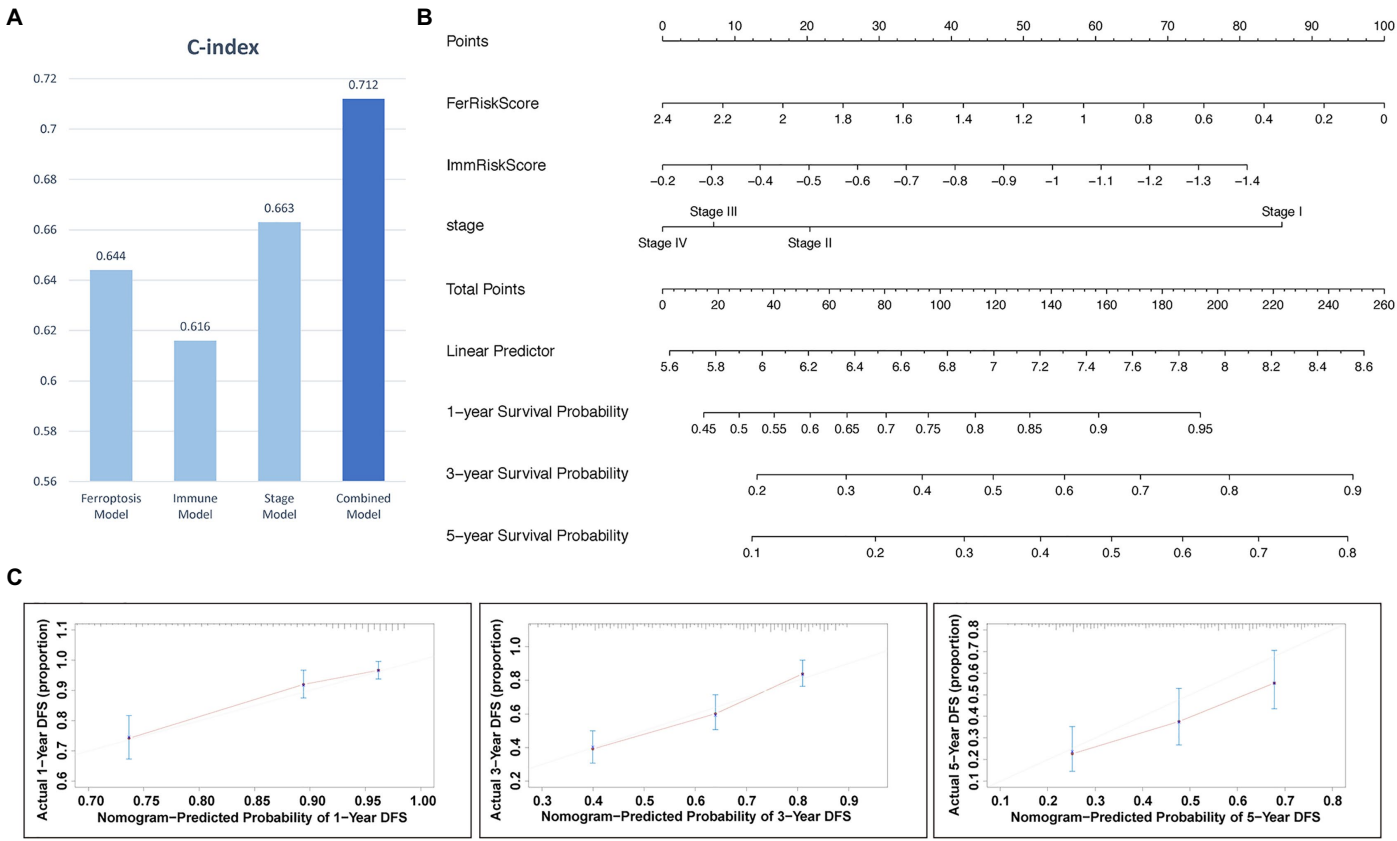
The nomogram for predicting the 1-, 3-, and 5-year survival probability of patients with LUAD. **(A)** Comparison of C index among models. **(B)** Prognostic nomogram for predicting 1-, 3-, and 5-years OS probability of LUAD. **(C)** Calibration curves of the nomogram for assessing predicted OS at 1-, 3-, and 5- years in the TCGA cohort.

The above results above showed that tumor stage, FRGs risk score, and IRGs risk score were independent prognostic factors in LUAD. The c-indices of the stage, ferroptosis, immune and combined models were 0.663, 0.644, 0.616, and 0.712, respectively (Figure 6A). Therefore, the combined model was selected for prediction of 1-, 3-, and 5-year OS rates (Figure 6B). Calibration plots showed that the combined model performed well in predicting 1- and 3-year survival but not 5-year survival (Figure 6C). Taken together, compared with models established using a single prognostic factor, the combined model was superior for short-term survival prediction, which might be beneficial to diagnosis and treatment.

### Relationship Between the FRGs and the IRGs in the Prognostic Risk Models

**Figure 7:**
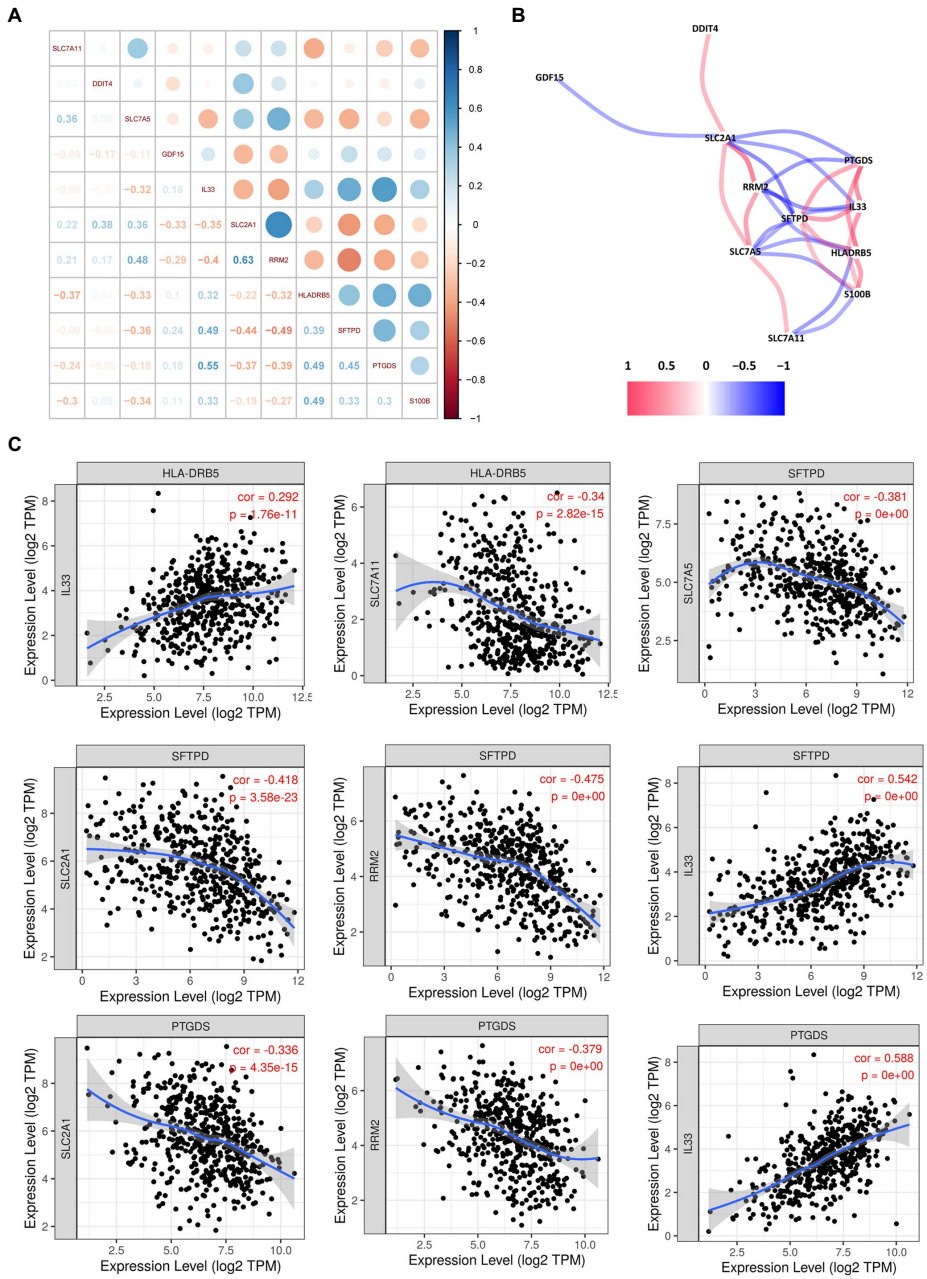
Correlation analysis among the hub genes. **(A)** Correlation diagram of the likely relationships among the hub genes. **(B)** Correlation network of the hub genes. **(C)** Association between the expression levels of hub genes.

To identify interactions between FRGs and IRGs, we performed a correlation analysis of gene expression (Figure 7A). Interleukin 33 (IL33) was synergistically co-expressed with HLA-DRB5, SFTPD, PTGDS, and S100B, and their high expression levels helped to prolong patient survival (Figure 7B). The association between IL33 and prostaglandin D2 synthase (PTGDS) was the strongest among all genes, followed by IL33 and surfactant protein D (SFTPD). All the other FRGs were negatively correlated with IRGs. This result was consistent with their opposite effects on survival time. Similar results were observed in the TIMER databases (Figure 7C).

### Validation of Gene Expression by qRT-PCR

**Figure 8:**
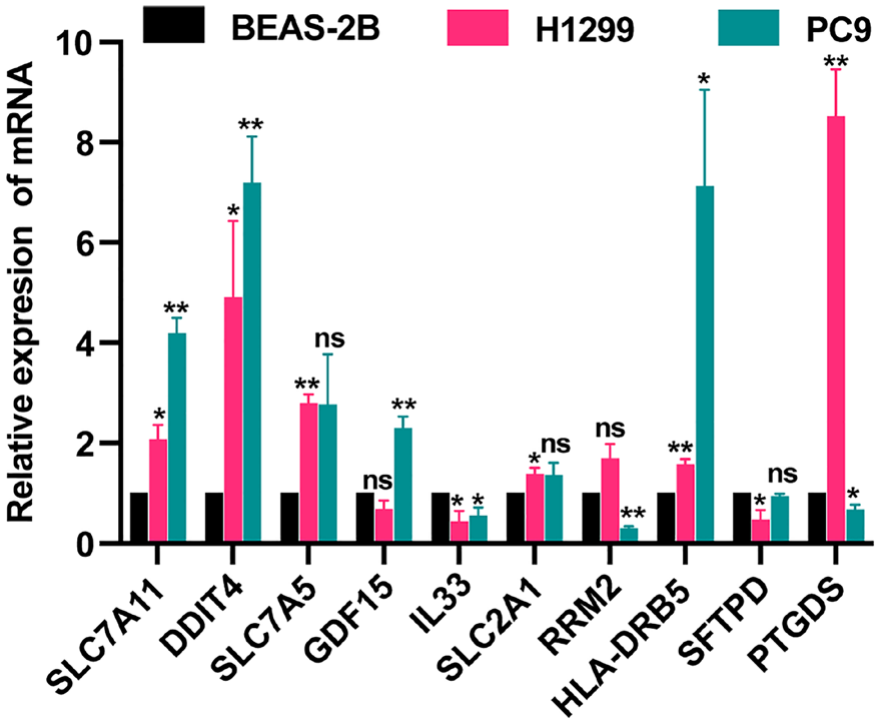
Validation of expression of FRGs and IRGs by qRT-PCR. ^*^p<0.05 and ^**^p<0.01.

We detected the levels of 7 FRGs and 4 IRGs in the BEAS-2B, PC9, and H1299 cell lines by using qRT-PCR (Figure 8). SLC7A11, DDIT4, SLC7A5, GDF15 SLC2A1, and HLA-DRB5 were highly expressed in LUAD cell lines. The mRNA levels of IL33, RRM2, and SFTPD were downregulated in LUAD cell lines. PTGDS was highly expressed in the H1299 cell line but low in PC9 cell line. The above results were generally consistent with the TCGA results, which indicates that our bioinformatics analysis was credible.

## Discussion

Non-small cell lung cancer (NSCLC) is one of the deadliest form of cancers. LUAD and lung squamous cell carcinoma (LUSC) are the predominant histological phenotypes of NSCLC, and they exhibit significant differences in morphologic differentiation, underlying drivers, and response to various therapies (Wilkerson et al., 2010; Cancer Genome Atlas Research Network, 2014). In recent years, the incidence of LUAD has significantly increased compared with LUSC (Kinoshita et al., 2016). The possible benefits and potential harm of inducing ferroptosis during treatment are receiving increasing attention. Cell death in LUAD in response to treatment with siramesine and lapatinib has been reported to be mediated by ferroptosis (Villalpando-Rodriguez et al., 2019). Additionally, triggering ferroptosis increased sensitivity to radiotherapy in human patient-derived models of LUAD (Ye et al., 2020). This suggests that new therapies that combine immunotherapy with regulation of ferroptosis may lead to improved outcomes. Therefore, studies exploring the interaction between ferroptosis and immunity in LUAD may have far-reaching implications.

The ferroptosis-related risk score calculation formulas used here contained seven FRGs. The qRT-PCR results showed that five genes were upregulated and two genes were downregulated. Except for RRM2, the other results were consistent with TCGA results. SLC7A11 is overexpressed in multiple types of cancer (Shi et al., 2019), and suppressing transcription and protein expression of SLC7A11 can effectively induce ferroptosis in cancer cells (Chang et al., 2018). DDIT4 has potential not only as a prognostic biomarker (Ho et al., 2020) but also as a therapeutic target (Wang et al., 2015). Paradoxically, upregulation of DDIT4 may promote cellular ferroptosis (Dixon et al., 2014), and therefore its specific role in cancer merits further exploration. Inhibition of SLC7A5 can regulate amino acid transport and affect ferroptosis (Dixon et al., 2014). Ansari et al. (2020) found that there is a significant correlation between overexpression of SLC7A5 and specific immune cell subtypes. Their study not only provided clinical evidence that SLC7A5 in breast cancer can aid the personalization of anti-PD1/PDL1 inhibition therapies but also suggested that targeting SLC7A5 may enhance the efficacy of anti-PDL1 immunotherapy. GDF15 may promote ferroptosis (Dixon et al., 2014), and some studies indicate that GDF15 plays an anti-cancer role, but other data suggest that it may promote tumor progression and metastasis (Tsui et al., 2015; Wang et al., 2019b). In a study of acute kidney injury, Martin-Sanchez et al. (2017) found that IL-33 release was associated with ferroptosis, leading them to hypothesize that ferroptosis may regulate inflammation by activating IL-33 *in vivo*. Moreover, tumor-derived IL33 enhanced the antitumor effects of checkpoint inhibitors (Chen et al., 2020). SLC2A1 can be activated by lymphoid-specific helicase (LSH) to inhibit ferroptosis (Jiang et al., 2017), and high SLC2A1 expression usually correlates with poorer patient outcomes (Kawamura et al., 2001; Tohma et al., 2005). RRM2 was reported to be highly expressed in liver cancer tissues and to prevent ferroptosis (Zhang et al., 2019), and upregulation of RRM2 in NSCLC cells promoted proliferation and chemotherapeutic resistance (Huang et al., 2019).

The immune-related risk score calculation formulas used here contained four IRGs. The HLA-DRB5 alleles were associated with cervical neoplasia through a linkage disequilibrium with amino acid variations and HLA-DRB1 alleles (Bao et al., 2018). HLA-DRB5 has also been considered a possible prognostic factor for gastric cancer (Hang et al., 2018). High expression of SFTPD might function to prevent progression of lung cancer (Yamaguchi et al., 2011). Increased SFTPD levels have been shown to be associated with fewer distant metastases and progression-free survival in LUAD that harbors EGFR mutations (Umeda et al., 2017). The low expression of SFTPD in H1299 cells and TCGA samples indicates that SFTPD may be a protective factor in lung adenocarcinoma patients but is inhibited in tumor tissue. Prostaglandin H2 is converted to prostaglandin D2 (PGD2) under the catalytic action of PTGDS (Fukuhara et al., 2012). PGD2 has previously been shown to inhibit migration of cancer cells (Shyu et al., 2013). This effect can be achieved by influencing immune responses (Fagerberg et al., 2014). S100B is significantly downregulated in esophageal squamous cell carcinoma, and may cause cell growth stagnation and apoptosis through synergistic action with p53 (Ji et al., 2004). CacyBP/SIP is a target protein of S100B and an inhibitor of gastric cancer (Ning et al., 2007).

Ferroptosis seems to be involved in the immune response to tumors. We observed that expression of the FRGs that inhibit ferroptosis negatively correlated with expression of IRGs. IL33 expression was consistent with the presence of ferroptosis and positively correlated with expression of IRGs. These results might suggest that ferroptosis had a positive relationship with anti-tumor immunity in LUAD. It has been shown that reduced uptake of cystine results in increased ferroptosis of tumor cells after tumor immunotherapy, which helps improve anti-tumor efficacy (Wang et al., 2019a). This means that the immune system can drive ferroptosis to mediate inhibition of tumor growth. By calculating the abundance of immune cells, it can be shown that the two systems overlap in differences in the levels of multiple types of immune cells. Xu et al. (2020) found that IFN- γ secreted in tumor tissues by infiltrating lymphocytes can help downregulate expression of SLC7A11, thereby promoting tumor cell ferroptosis and decreasing tumor volume. Efimova et al. (2020) observed that dendritic cells can be induced to mature and active by phagocytosis of early ferroptotic cancer cells. Further exploration of the clinical relevance of ferroptosis- and immune-related marker genes, and potential connection between them, would help to identify more efficient diagnostic and therapeutic approaches to LUAD.

Although many studies have attempted to elucidate an association between ferroptosis and immunity, there is no report of combining two variables for predicting OS of LUAD patients. At present, prognosis in LUAD is based on tumor stage. In the present study, the nomogram composed of tumor staging combined with FRGs and IRGs-related risk scores provided more precise prediction of patient outcomes. However, there are several limitations. Firstly, the data used in this work were all downloaded from public databases. The results should also be externally validated with other primary data. Secondly, although we have found some similarity in immune function, further understanding of functional connections between the seven FRGs and four IRGs identified here requires additional experimental study.

In conclusion, our study identified seven FRGs and four IRGs that are differentially expressed in LUAD and significantly associated with prognosis. A nomogram that combines these sets of genes is more beneficial to individualized prognosis in LUAD than the current standard that uses a single prognostic factor. Using Pearson correlation analysis, we inferred that immune response is positively correlated with ferroptosis in LUAD. These results provide valuable information for development of new therapies that combine immunotherapy with ferroptosis-related drugs.

## Data Availability Statement

Publicly available datasets were analyzed in this study. This data can be found at: TCGA database: https://portal.gdc.cancer.gov/repository. GSE13213 was from GEO database: https://www.ncbi.nlm.nih.gov/geo/.

## Author Contributions

MC and XL designed the study, executed the bioinformatics analysis, and prepared figures and tables. YZ and YT collected the data. MC, XL, YZ, PS, and JL wrote parts of the manuscript. XH, LW, and KS revised the manuscript. All authors contributed to the article and approved the submitted version.

## Conflict of Interest

The authors declare that the research was conducted in the absence of any commercial or financial relationships that could be construed as a potential conflict of interest.

## Publisher’s Note

All claims expressed in this article are solely those of the authors and do not necessarily represent those of their affiliated organizations, or those of the publisher, the editors and the reviewers. Any product that may be evaluated in this article, or claim that may be made by its manufacturer, is not guaranteed or endorsed by the publisher.

## Footnotes

^1^ https://portal.gdc.cancer.gov/repository

^2^ https://www.ncbi.nlm.nih.gov/geo/

^3^ http://www.zhounan.org/ferrdb/

^4^ https://www.immport.org

^5^ https://cistrome.shinyapps.io/timer/

## Abbreviations

LUAD: Lung adenocarcinoma
FRGs: Ferroptosis-related genes
IRGs: Immune-related genes
GPX4: Glutathione peroxidase 4
TCGA: The Cancer Genome Atlas
GEO: The Gene Expression Omnibus
NSCLC: Non-small cell lung cancer
DEGs: Differentially expressed genes
ROC: Receiver operating characteristic
t-SNE: t-Distributed stochastic neighbor embedding
PCA: Principal component analysis
OS: Overall survival
C-index: Concordance index
GO: Gene Ontology
KEGG: Kyoto Encyclopedia of Genes and Genomes
LUSC: Lung squamous cell carcinoma

## References

[ref1] Ansari R. E. Craze M. L. Althobiti M. Alfarsi L. Ellis I. O. Rakha E. A.. (2020). Enhanced glutamine uptake influences composition of immune cell infiltrates in breast cancer. Br. J. Cancer 122, 94–101. 10.1038/s41416-019-0626-z, PMID: 31819174 PMC6964696

[ref2] Badgley M. A. Kremer D. M. Maurer H. C. DelGiorno K. E. Lee H. J. Purohit V.. (2020). Cysteine depletion induces pancreatic tumor ferroptosis in mice. Science 368, 85–89. 10.1126/science.aaw9872, PMID: 32241947 PMC7681911

[ref3] Bao X. Hanson A. L. Madeleine M. M. Wang S. S. Schwartz S. M. Newell F.. (2018). HLA and KIR associations of cervical neoplasia. J. Infect. Dis. 218, 2006–2015. 10.1093/infdis/jiy483, PMID: 30099516 PMC6217726

[ref4] Cancer Genome Atlas Research Network (2014). Comprehensive molecular profiling of lung adenocarcinoma. Nature 511, 543–550. 10.1038/nature13385, PMID: 25079552 PMC4231481

[ref5] Chang L. C. Chiang S. K. Chen S. E. Yu Y. L. Chou R. H. Chang W. C. (2018). Heme oxygenase-1 mediates BAY 11-7085 induced ferroptosis. Cancer Lett. 416, 124–137. 10.1016/j.canlet.2017.12.025, PMID: 29274359

[ref6] Chen L. Sun R. Xu J. Zhai W. Zhang D. Yang M.. (2020). Tumor-derived IL33 promotes tissue-resident CD8(+) T cells and is required for checkpoint blockade tumor immunotherapy. Cancer Immunol. Res. 8, 1381–1392. 10.1158/2326-6066.CIR-19-1024, PMID: 32917659 PMC7642190

[ref7] Dai E. Han L. Liu J. Xie Y. Zeh H. J. Kang R.. (2020). Ferroptotic damage promotes pancreatic tumorigenesis through a TMEM173/STING-dependent DNA sensor pathway. Nat. Commun. 11:6339. 10.1038/s41467-020-20154-8, PMID: 33311482 PMC7732843

[ref8] Dixon S. J. Patel D. N. Welsch M. Skouta R. Lee E. D. Hayano M.. (2014). Pharmacological inhibition of cystine-glutamate exchange induces endoplasmic reticulum stress and ferroptosis. elife 3:e02523. 10.7554/eLife.02523, PMID: 24844246 PMC4054777

[ref9] Efferth T. (2017). From ancient herb to modern drug: Artemisia annua and artemisinin for cancer therapy. Semin. Cancer Biol. 46, 65–83. 10.1016/j.semcancer.2017.02.009, PMID: 28254675

[ref10] Efimova I. Catanzaro E. Van der Meeren L. Turubanova V. D. Hammad H. Mishchenko T. A.. (2020). Vaccination with early ferroptotic cancer cells induces efficient antitumor immunity. J. Immunother. Cancer 8:e001369. 10.1136/jitc-2020-001369, PMID: 33188036 PMC7668384

[ref11] Fagerberg L. Hallström B. M. Oksvold P. Kampf C. Djureinovic D. Odeberg J.. (2014). Analysis of the human tissue-specific expression by genome-wide integration of transcriptomics and antibody-based proteomics. Mol. Cell. Proteomics 13, 397–406. 10.1074/mcp.M113.035600, PMID: 24309898 PMC3916642

[ref12] Fukuhara A. Yamada M. Fujimori K. Miyamoto Y. Kusumoto T. Nakajima H.. (2012). Lipocalin-type prostaglandin D synthase protects against oxidative stress-induced neuronal cell death. Biochem. J. 443, 75–84. 10.1042/BJ20111889, PMID: 22248185

[ref13] Hang X. Li D. Wang J. Wang G. (2018). Prognostic significance of microsatellite instability-associated pathways and genes in gastric cancer. Int. J. Mol. Med. 42, 149–160. 10.3892/ijmm.2018.3643, PMID: 29717769 PMC5979886

[ref14] Herbst R. S. Morgensztern D. Boshoff C. (2018). The biology and management of non-small cell lung cancer. Nature 553, 446–454. 10.1038/nature25183, PMID: 29364287

[ref15] Ho K. H. Chen P. H. Chou C. M. Shih C. M. Lee Y. T. Cheng C. H.. (2020). A key role of DNA damage-inducible transcript 4 (DDIT4) connects autophagy and GLUT3-mediated stemness to desensitize temozolomide efficacy in glioblastomas. Neurotherapeutics 17, 1212–1227. 10.1007/s13311-019-00826-0, PMID: 31916238 PMC7609792

[ref16] Huang N. Guo W. Ren K. Li W. Jiang Y. Sun J.. (2019). LncRNA AFAP1-AS1 supresses miR-139-5p and promotes cell proliferation and chemotherapy resistance of non-small cell lung cancer by competitively upregulating RRM2. Front. Oncol. 9:1103. 10.3389/fonc.2019.01103, PMID: 31696057 PMC6817562

[ref17] Ji J. Zhao L. Wang X. Zhou C. Ding F. Su L.. (2004). Differential expression of S100 gene family in human esophageal squamous cell carcinoma. J. Cancer Res. Clin. Oncol. 130, 480–486. 10.1007/s00432-004-0555-x, PMID: 15185146 PMC12161862

[ref18] Jiang Y. Mao C. Yang R. Yan B. Shi Y. Liu X.. (2017). EGLN1/c-Myc induced lymphoid-specific helicase inhibits ferroptosis through lipid metabolic gene expression changes. Theranostics 7, 3293–3305. 10.7150/thno.19988, PMID: 28900510 PMC5595132

[ref19] Kawamura T. Kusakabe T. Sugino T. Watanabe K. Fukuda T. Nashimoto A.. (2001). Expression of glucose transporter-1 in human gastric carcinoma: association with tumor aggressiveness, metastasis, and patient survival. Cancer 92, 634–641. 10.1002/1097-0142(20010801)92:3<634::AID-CNCR1364>3.0.CO;2-X, PMID: 11505409

[ref20] Kinoshita F. L. Ito Y. Nakayama T. (2016). Trends in lung cancer incidence rates by histological type in 1975-2008: a population-based study in Osaka, Japan. J. Epidemiol. 26, 579–586. 10.2188/jea.JE20150257, PMID: 27150013 PMC5083321

[ref21] Li Z. Rong L. (2020). Cascade reaction-mediated efficient ferroptosis synergizes with immunomodulation for high-performance cancer therapy. Biomater. Sci. 8, 6272–6285. 10.1039/D0BM01168A, PMID: 33016289

[ref22] Ma J. Ward E. M. Smith R. Jemal A. (2013). Annual number of lung cancer deaths potentially avertable by screening in the United States. Cancer 119, 1381–1385. 10.1002/cncr.27813, PMID: 23440730

[ref23] Martin-Sanchez D. Ruiz-Andres O. Poveda J. Carrasco S. Cannata-Ortiz P. Sanchez-Niño M. D.. (2017). Ferroptosis, but not necroptosis, is important in nephrotoxic folic acid-induced AKI. J. Am. Soc. Nephrol. 28, 218–229. 10.1681/asn.2015121376, PMID: 27352622 PMC5198282

[ref24] Matsushita M. Freigang S. Schneider C. Conrad M. Bornkamm G. W. Kopf M. (2015). T cell lipid peroxidation induces ferroptosis and prevents immunity to infection. J. Exp. Med. 212, 555–568. 10.1084/jem.20140857, PMID: 25824823 PMC4387287

[ref25] Ning X. Sun S. Hong L. Liang J. Liu L. Han S.. (2007). Calcyclin-binding protein inhibits proliferation, tumorigenicity, and invasion of gastric cancer. Mol. Cancer Res. 5, 1254–1262. 10.1158/1541-7786.MCR-06-0426, PMID: 18171983

[ref26] Shi Z. Z. Fan Z. W. Chen Y. X. Xie X. F. Jiang W. Wang W. J.. (2019). Ferroptosis in carcinoma: regulatory mechanisms and new method for cancer therapy. Onco Targets Ther. 12, 11291–11304. 10.2147/ott.S232852, PMID: 31908494 PMC6927606

[ref27] Shyu R. Y. Wu C. C. Wang C. H. Tsai T. C. Wang L. K. Chen M. L.. (2013). H-rev107 regulates prostaglandin D2 synthase-mediated suppression of cellular invasion in testicular cancer cells. J. Biomed. Sci. 20:30. 10.1186/1423-0127-20-30, PMID: 23687991 PMC3669107

[ref28] Stockwell B. R. Friedmann Angeli J. P. Bayir H. Bush A. I. Conrad M. Dixon S. J.. (2017). Ferroptosis: a regulated cell death nexus linking metabolism, redox biology, and disease. Cell 171, 273–285. 10.1016/j.cell.2017.09.021, PMID: 28985560 PMC5685180

[ref29] Sung H. Ferlay J. Siegel R. L. Laversanne M. Soerjomataram I. Jemal A.. (2021). Global cancer statistics 2020: GLOBOCAN estimates of incidence and mortality worldwide for 36 cancers in 185 countries. CA Cancer J. Clin. 71, 209–249. 10.3322/caac.21660, PMID: 33538338

[ref30] Tohma T. Okazumi S. Makino H. Cho A. Mochizuki R. Shuto K.. (2005). Overexpression of glucose transporter 1 in esophageal squamous cell carcinomas: a marker for poor prognosis. Dis. Esophagus 18, 185–189. 10.1111/j.1442-2050.2005.00489.x, PMID: 16045581

[ref31] Tsui K. H. Hsu S. Y. Chung L. C. Lin Y. H. Feng T. H. Lee T. Y.. (2015). Growth differentiation factor-15: a p53- and demethylation-upregulating gene represses cell proliferation, invasion, and tumorigenesis in bladder carcinoma cells. Sci. Rep. 5:12870. 10.1038/srep12870, PMID: 26249737 PMC4528199

[ref32] Umeda Y. Hasegawa Y. Otsuka M. Ariki S. Takamiya R. Saito A.. (2017). Surfactant protein D inhibits activation of non-small cell lung cancer-associated mutant EGFR and affects clinical outcomes of patients. Oncogene 36, 6432–6445. 10.1038/onc.2017.253, PMID: 28745320

[ref33] Villalpando-Rodriguez G. E. Blankstein A. R. Konzelman C. Gibson S. B. (2019). Lysosomal destabilizing drug siramesine and the dual tyrosine kinase inhibitor lapatinib induce a synergistic ferroptosis through reduced heme oxygenase-1 (HO-1) levels. Oxidative Med. Cell. Longev. 2019:9561281. 10.1155/2019/9561281, PMID: 31636810 PMC6766165

[ref34] Wang D. DuBois R. N. (2015). Immunosuppression associated with chronic inflammation in the tumor microenvironment. Carcinogenesis 36, 1085–1093. 10.1093/carcin/bgv123, PMID: 26354776 PMC5006153

[ref35] Wang W. Green M. Choi J. E. Gijón M. Kennedy P. D. Johnson J. K.. (2019a). CD8(+) T cells regulate tumour ferroptosis during cancer immunotherapy. Nature 569, 270–274. 10.1038/s41586-019-1170-y, PMID: 31043744 PMC6533917

[ref36] Wang Y. Han E. Xing Q. Yan J. Arrington A. Wang C.. (2015). Baicalein upregulates DDIT4 expression which mediates mTOR inhibition and growth inhibition in cancer cells. Cancer Lett. 358, 170–179. 10.1016/j.canlet.2014.12.033, PMID: 25543165 PMC5989711

[ref37] Wang W. Yang X. Dai J. Lu Y. Zhang J. Keller E. T. (2019b). Prostate cancer promotes a vicious cycle of bone metastasis progression through inducing osteocytes to secrete GDF15 that stimulates prostate cancer growth and invasion. Oncogene 38, 4540–4559. 10.1038/s41388-019-0736-3, PMID: 30755731 PMC9097780

[ref38] Wilkerson M. D. Yin X. Hoadley K. A. Liu Y. Hayward M. C. Cabanski C. R.. (2010). Lung squamous cell carcinoma mRNA expression subtypes are reproducible, clinically important, and correspond to normal cell types. Clin. Cancer Res. 16, 4864–4875. 10.1158/1078-0432.CCR-10-0199, PMID: 20643781 PMC2953768

[ref39] Xu T. Ma Y. Yuan Q. Hu H. Hu X. Qian Z.. (2020). Enhanced ferroptosis by oxygen-boosted phototherapy based on a 2-in-1 nanoplatform of ferrous hemoglobin for tumor synergistic therapy. ACS Nano 14, 3414–3425. 10.1021/acsnano.9b09426, PMID: 32155051

[ref40] Yamaguchi H. Soda H. Nakamura Y. Takasu M. Tomonaga N. Nakano H.. (2011). Serum levels of surfactant protein D predict the anti-tumor activity of gefitinib in patients with advanced non-small cell lung cancer. Cancer Chemother. Pharmacol. 67, 331–338. 10.1007/s00280-010-1325-x, PMID: 20401612

[ref41] Yang W. S. SriRamaratnam R. Welsch M. E. Shimada K. Skouta R. Viswanathan V. S.. (2014). Regulation of ferroptotic cancer cell death by GPX4. Cell 156, 317–331. 10.1016/j.cell.2013.12.010, PMID: 24439385 PMC4076414

[ref42] Ye L. F. Chaudhary K. R. Zandkarimi F. Harken A. D. Kinslow C. J. Upadhyayula P. S.. (2020). Radiation-induced lipid peroxidation triggers ferroptosis and synergizes with ferroptosis inducers. ACS Chem. Biol. 15, 469–484. 10.1021/acschembio.9b00939, PMID: 31899616 PMC7180072

[ref43] Zhang X. Du L. Qiao Y. Zhang X. Zheng W. Wu Q.. (2019). Ferroptosis is governed by differential regulation of transcription in liver cancer. Redox Biol. 24:101211. 10.1016/j.redox.2019.101211, PMID: 31108460 PMC6526247

