# Supplementary material for: A Nomogram Integrating Ferroptosis- and Immune-Related Biomarkers for Prediction of Overall Survival in Lung Adenocarcinoma
Source: Front Genet. 2021 Sep 1;12:706814. doi: 10.3389/fgene.2021.706814 (PMC8441018; doi:10.3389/fgene.2021.706814)

Supplementary Table 1. Summary of ferroptosis-related genes.

| No. | Gene Symbol | No. | Gene Symbol | No. | Gene Symbol | No. | Gene Symbol | No. | Gene Symbol |
| --- | --- | --- | --- | --- | --- | --- | --- | --- | --- |
| 1 | PTGS2 | 26 | TXNIP | 51 | LOC390705 | 76 | NFE2L2 | 101 | HMOX1 |
| 2 | DUSP1 | 27 | VLDLR | 52 | EIF2S1 | 77 | STEAP3 | 102 | NFE2L2 |
| 3 | NOS2 | 28 | GPT2 | 53 | KIM-1 | 78 | DRD5 | 103 | ELAVL1 |
| 4 | NCF2 | 29 | PSAT1 | 54 | IL6 | 79 | GPX4 | 104 | SLC3A2 |
| 5 | MT3 | 30 | LURAP1L | 55 | CXCL2 | 80 | DRD4 | 105 | SLC7A11 |
| 6 | UBC | 31 | SLC7A5 | 56 | RELA | 81 | MAP3K5 | 106 | TFAP2C |
| 7 | ALB | 32 | HERPUD1 | 57 | HSD17B11 | 82 | MAPK14 | 107 | SP1 |
| 8 | TXNRD1 | 33 | XBP1 | 58 | AGPAT3 | 83 | SLC2A1 | 108 | HBA1 |
| 9 | SRXN1 | 34 | ATF3 | 59 | SETD1B | 84 | SLC2A3 | 109 | NNMT |
| 10 | GPX2 | 35 | SLC3A2 | 60 | HMOX1 | 85 | SLC2A6 | 110 | PLIN4 |
| 11 | BNIP3 | 36 | CBS | 61 | TF | 86 | SLC2A8 | 111 | HIC1 |
| 12 | OXSR1 | 37 | ATF4 | 62 | FTL | 87 | SLC2A12 | 112 | STMN1 |
| 13 | SELENOS | 38 | ZNF419 | 63 | RPL8 | 88 | GLUT13 | 113 | RRM2 |
| 14 | ANGPTL7 | 39 | KLHL24 | 64 | ATP5MC3 | 89 | SLC2A14 | 114 | CAPG |
| 15 | CHAC1 | 40 | TRIB3 | 65 | TFRC | 90 | EIF2AK4 | 115 | HNF4A |
| 16 | SLC7A11 | 41 | ZFP69B | 66 | MAFG | 91 | EIF2S1 | 116 | NGB |
| 17 | DDIT4 | 42 | ATP6V1G2 | 67 | IL33 | 92 | ATF4 | 117 | YWHAE |
| 18 | LOC284561 | 43 | VEGFA | 68 | FTH1 | 93 | ALOX5 | 118 | GABPB1 |
| 19 | ASNS | 44 | GDF15 | 69 | SLC40A1 | 94 | ALOX12 | 119 | AURKA |
| 20 | TSC22D3 | 45 | TUBE1 | 70 | TF | 95 | ALOX15 | 120 | MIR4715 |
| 21 | DDIT3 | 46 | ARRDC3 | 71 | TFRC | 96 | ALOX5 | 121 | RIPK1 |
| 22 | JDP2 | 47 | CEBPG | 72 | FTH1 | 97 | ACSF2 | 122 | PRDX1 |
| 23 | SESN2 | 48 | SNORA16A | 73 | GPX4 | 98 | IREB2 | 123 | MIR30B |
| 24 | SLC1A4 | 49 | RGS4 | 74 | HAMP | 99 | GPX4 |  |  |
| 25 | PCK2 | 50 | BLOC1S5-TXNDC5 | 75 | HSPB1 | 100 | HMGB1 |  |  |

Supplementary Table 2. Summary of immune-related genes.

| No. | Gene Symbol | No. | Gene Symbol | No. | Gene Symbol | No. | Gene Symbol | No. | Gene Symbol |
| --- | --- | --- | --- | --- | --- | --- | --- | --- | --- |
| 1 | AZGP1 | 498 | APOBEC3H | 995 | CXCL11 | 1492 | SAA2 | 1989 | KIR3DL2 |
| 2 | B2M | 499 | TMPRSS6 | 996 | CXCL12 | 1493 | SBDS | 1990 | KIR2DL1 |
| 3 | CALR | 500 | SPINK5 | 997 | CXCL13 | 1494 | SCG2 | 1991 | KIR2DL2 |
| 4 | CANX | 501 | MARCO | 998 | CXCL14 | 1495 | SCGB3A1 | 1992 | KIR2DL3 |
| 5 | CD1A | 502 | BECN1 | 999 | CXCL16 | 1496 | SCT | 1993 | KIR2DL4 |
| 6 | CD1B | 503 | TNFSF11 | 1000 | CXCL17 | 1497 | AIMP1 | 1994 | KIR2DL5A |
| 7 | CD1C | 504 | KNG1 | 1001 | CXCL2 | 1498 | SECTM1 | 1995 | KLRC1 |
| 8 | CD1D | 505 | CSK | 1002 | CXCL3 | 1499 | SEMA3A | 1996 | KLRC2 |
| 9 | CD1E | 506 | KLRK1 | 1003 | CXCL5 | 1500 | SEMA3B | 1997 | KLRC3 |
| 10 | CD4 | 507 | KCNH2 | 1004 | CXCL6 | 1501 | SEMA3C | 1998 | KLRD1 |
| 11 | CD8A | 508 | JUND | 1005 | CXCL9 | 1502 | SEMA3D | 1999 | PTPN6 |
| 12 | CD8B | 509 | JAK1 | 1006 | CCN1 | 1503 | SEMA3E | 2000 | PTPN11 |
| 13 | CD74 | 510 | CREB1 | 1007 | DEFA1 | 1504 | SEMA3F | 2001 | ICAM1 |
| 14 | CREB1 | 511 | CLDN4 | 1008 | DEFA3 | 1505 | SEMA3G | 2002 | ICAM2 |
| 15 | CTSB | 512 | CCL28 | 1009 | DEFA5 | 1506 | SEMA4A | 2003 | ITGAL |
| 16 | CTSE | 513 | RNASE3 | 1010 | DEFB1 | 1507 | SEMA4B | 2004 | ITGB2 |
| 17 | CTSL | 514 | RN7SL1 | 1011 | DEFB103B | 1508 | SEMA4C | 2005 | PTK2B |
| 18 | CTSS | 515 | IRF7 | 1012 | DEFB104A | 1509 | SEMA4D | 2006 | VAV3 |
| 19 | FCER1G | 516 | IREB2 | 1013 | DEFB4A | 1510 | SEMA4F | 2007 | VAV1 |
| 20 | FCGRT | 517 | ILK | 1014 | EDN1 | 1511 | SEMA4G | 2008 | VAV2 |
| 21 | PDIA3 | 518 | IL18 | 1015 | EDN2 | 1512 | SEMA5A | 2009 | RAC1 |
| 22 | HFE | 519 | IL17A | 1016 | EDN3 | 1513 | SEMA5B | 2010 | RAC2 |
| 23 | HLA-A | 520 | LTB4R | 1017 | FGF10 | 1514 | SEMA6A | 2011 | RAC3 |
| 24 | HLA-B | 521 | APOBEC3A | 1018 | FGF2 | 1515 | SEMA6B | 2012 | PAK1 |
| 25 | HLA-C | 522 | MASP2 | 1019 | HTN3 | 1516 | SEMA6C | 2013 | MAP2K1 |
| 26 | HLA-DMA | 523 | TRIM27 | 1020 | CXCL8 | 1517 | SEMA6D | 2014 | MAP2K2 |
| 27 | HLA-DMB | 524 | RELA | 1021 | LECT2 | 1518 | SEMA7A | 2015 | MAPK1 |
| 28 | HLA-DOA | 525 | IL7R | 1022 | PF4 | 1519 | SLIT1 | 2016 | MAPK3 |
| 29 | HLA-DOB | 526 | IL1A | 1023 | PF4V1 | 1520 | SLIT2 | 2017 | TNF |
| 30 | HLA-DPA1 | 527 | PTX3 | 1024 | PLAU | 1521 | SLURP1 | 2018 | CSF2 |
| 31 | HLA-DPB1 | 528 | IFNAR2 | 1025 | PPBP | 1522 | SPP1 | 2019 | IFNG |
| 32 | HLA-DQA1 | 529 | IFN1@ | 1026 | PPBPP1 | 1523 | SST | 2020 | KIR2DS1 |
| 33 | HLA-DQA2 | 530 | SYTL1 | 1027 | PROK2 | 1524 | STC1 | 2021 | KIR2DS3 |
| 34 | HLA-DQB1 | 531 | APOBEC3C | 1028 | RNASE2 | 1525 | STC2 | 2022 | KIR2DS4 |
| 35 | HLA-DRA | 532 | DDX17 | 1029 | SAA1 | 1526 | TAC1 | 2023 | KIR2DS5 |
| 36 | HLA-DRB1 | 533 | PTGS2 | 1030 | SAA2 | 1527 | TDGF1 | 2024 | NCR2 |
| 37 | HLA-DRB3 | 534 | HTR1A | 1031 | SBDS | 1528 | TDGF1P3 | 2025 | TYROBP |
| 38 | HLA-DRB4 | 535 | SEPTIN7 | 1032 | SEMA3A | 1529 | TG | 2026 | LCK |
| 39 | HLA-DRB5 | 536 | CD40LG | 1033 | SEMA3B | 1530 | TGFA | 2027 | FCGR3A |
| 40 | HLA-E | 537 | CD14 | 1034 | SEMA3C | 1531 | TGFB1 | 2028 | FCGR3B |
| 41 | HLA-F | 538 | CD8A | 1035 | SEMA3D | 1532 | TGFB2 | 2029 | NCR1 |
| 42 | HLA-G | 539 | CD4 | 1036 | SEMA3E | 1533 | TGFB3 | 2030 | NCR3 |
| 43 | HLA-H | 540 | MASP1 | 1037 | SEMA3F | 1534 | THPO | 2031 | FCER1G |
| 44 | MR1 | 541 | PROC | 1038 | SEMA3G | 1535 | TNC | 2032 | CD247 |
| 45 | HSPA1A | 542 | MAP2K2 | 1039 | SEMA4A | 1536 | TNF | 2033 | ZAP70 |
| 46 | HSPA1B | 543 | MAP2K1 | 1040 | SEMA4B | 1537 | TNFRSF11B | 2034 | SYK |
| 47 | HSPA1L | 544 | HRG | 1041 | SEMA4C | 1538 | TNFSF10 | 2035 | LCP2 |
| 48 | HSPA2 | 545 | NDRG1 | 1042 | SEMA4D | 1539 | TNFSF11 | 2036 | LAT |
| 49 | HSPA4 | 546 | IRF9 | 1043 | SEMA4F | 1540 | TNFSF12 | 2037 | PLCG1 |
| 50 | HSPA5 | 547 | TRIM22 | 1044 | SEMA4G | 1541 | TNFSF13 | 2038 | PLCG2 |
| 51 | HSPA6 | 548 | LANCL1 | 1045 | SEMA5A | 1542 | TNFSF13B | 2039 | SH3BP2 |
| 52 | HSPA8 | 549 | PPP4C | 1046 | SEMA5B | 1543 | TNFSF14 | 2040 | PIK3CA |
| 53 | HSP90AA1 | 550 | HMOX1 | 1047 | SEMA6A | 1544 | TNFSF15 | 2041 | PIK3CB |
| 54 | HSP90AB1 | 551 | HMGB1 | 1048 | SEMA6B | 1545 | TNFSF18 | 2042 | PIK3CD |
| 55 | ICAM1 | 552 | HLA-B | 1049 | SEMA6C | 1546 | TNFSF4 | 2043 | PIK3CG |
| 56 | IFNA1 | 553 | RNASE7 | 1050 | SEMA6D | 1547 | TNFSF8 | 2044 | PIK3R5 |
| 57 | IFNA2 | 554 | ABCC4 | 1051 | SEMA7A | 1548 | TNFSF9 | 2045 | PIK3R1 |
| 58 | IFNA4 | 555 | HGF | 1052 | SLIT1 | 1549 | TOR2A | 2046 | PIK3R2 |
| 59 | IFNA5 | 556 | HDAC1 | 1053 | SLIT2 | 1550 | TRH | 2047 | PIK3R3 |
| 60 | IFNA6 | 557 | IFNLR1 | 1054 | TNC | 1551 | TSHB | 2048 | FYN |
| 61 | IFNA7 | 558 | PLSCR1 | 1055 | TYMP | 1552 | TSLP | 2049 | SHC2 |
| 62 | IFNA8 | 559 | B2M | 1056 | XCL1 | 1553 | TXLNA | 2050 | SHC4 |
| 63 | IFNA10 | 560 | BACH2 | 1057 | XCL2 | 1554 | TYMP | 2051 | SHC3 |
| 64 | IFNA13 | 561 | TANK | 1058 | C5AR1 | 1555 | UCN | 2052 | SHC1 |
| 65 | IFNA14 | 562 | PIK3CG | 1059 | ACKR2 | 1556 | UCN2 | 2053 | GRB2 |
| 66 | IFNA16 | 563 | ARRB1 | 1060 | CCR1 | 1557 | UCN3 | 2054 | SOS1 |
| 67 | IFNA17 | 564 | RSAD2 | 1061 | CCR10 | 1558 | UTS2 | 2055 | SOS2 |
| 68 | IFNA21 | 565 | STAB2 | 1062 | CCR3 | 1559 | UTS2B | 2056 | HRAS |
| 69 | IFNG | 566 | TBK1 | 1063 | CCR4 | 1560 | VEGFA | 2057 | KRAS |
| 70 | KIR2DL1 | 567 | PDYN | 1064 | CCR5 | 1561 | VEGFB | 2058 | NRAS |
| 71 | KIR2DL2 | 568 | PDGFRB | 1065 | CCR6 | 1562 | VEGFC | 2059 | ARAF |
| 72 | KIR2DL3 | 569 | PDCD1 | 1066 | CCR7 | 1563 | VGF | 2060 | BRAF |
| 73 | KIR2DL4 | 570 | PCSK2 | 1067 | CCR8 | 1564 | VIP | 2061 | RAF1 |
| 74 | KIR2DS1 | 571 | PCSK1 | 1068 | CCR9 | 1565 | XCL1 | 2062 | MICA |
| 75 | KIR2DS3 | 572 | ARG2 | 1069 | ACKR4 | 1566 | XCL2 | 2063 | MICB |
| 76 | KIR2DS4 | 573 | AQP9 | 1070 | CCRL2 | 1567 | ACVR1B | 2064 | ULBP3 |
| 77 | KIR2DS5 | 574 | FASLG | 1071 | CMKLR1 | 1568 | ACVR1C | 2065 | ULBP2 |
| 78 | KIR3DL1 | 575 | APOH | 1072 | CX3CR1 | 1569 | ACVR2A | 2066 | ULBP1 |
| 79 | KIR3DL2 | 576 | BIRC5 | 1073 | CXCR3 | 1570 | ACVR2B | 2067 | KLRK1 |
| 80 | KLRC1 | 577 | ANXA6 | 1074 | CXCR4 | 1571 | ACVRL1 | 2068 | HCST |
| 81 | KLRC2 | 578 | IL22 | 1075 | CXCR5 | 1572 | ADCYAP1R1 | 2069 | CD48 |
| 82 | KLRC3 | 579 | VTN | 1076 | CXCR6 | 1573 | ADIPOR1 | 2070 | CD244 |
| 83 | KLRD1 | 580 | VIM | 1077 | ACKR3 | 1574 | ADIPOR2 | 2071 | PPP3CA |
| 84 | LTA | 581 | VCAM1 | 1078 | CYSLTR1 | 1575 | ADRB1 | 2072 | PPP3CB |
| 85 | CIITA | 582 | PRDX1 | 1079 | CYSLTR2 | 1576 | ADRB2 | 2073 | PPP3CC |
| 86 | MICA | 583 | GFAP | 1080 | ACKR1 | 1577 | AGTR1 | 2074 | CHP1 |
| 87 | MICB | 584 | GBP2 | 1081 | EDNRA | 1578 | AGTR2 | 2075 | PPP3R1 |
| 88 | NFYA | 585 | ALB | 1082 | EDNRB | 1579 | AMHR2 | 2076 | PPP3R2 |
| 89 | NFYB | 586 | SLC29A3 | 1083 | FPR1 | 1580 | ANGPT1 | 2077 | CHP2 |
| 90 | NFYC | 587 | OAS1 | 1084 | FPR2 | 1581 | ANGPT4 | 2078 | NFAT5 |
| 91 | LGMN | 588 | AGER | 1085 | FPR2 | 1582 | ANGPTL1 | 2079 | NFATC1 |
| 92 | PSMB8 | 589 | UNC93B1 | 1086 | GPR17 | 1583 | ANGPTL2 | 2080 | NFATC2 |
| 93 | PSMC1 | 590 | TNFSF4 | 1087 | GPR32 | 1584 | ANGPTL3 | 2081 | NFATC3 |
| 94 | PSMC2 | 591 | NOS1 | 1088 | GPR33 | 1585 | ANGPTL4 | 2082 | NFATC4 |
| 95 | PSMC3 | 592 | ACTG1 | 1089 | PTGDR2 | 1586 | ANGPTL6 | 2083 | PRKCA |
| 96 | PSMC4 | 593 | ACTA1 | 1090 | C5AR2 | 1587 | APLNR | 2084 | PRKCB |
| 97 | PSMC5 | 594 | ACO1 | 1091 | CXCR1 | 1588 | AR | 2085 | PRKCG |
| 98 | PSMC6 | 595 | SERPINA3 | 1092 | CXCR2 | 1589 | AVPR1A | 2086 | SH2D1B |
| 99 | PSMD1 | 596 | CXCR1 | 1093 | LTB4R | 1590 | AVPR1B | 2087 | SH2D1A |
| 100 | PSMD2 | 597 | CCL15 | 1094 | LTB4R2 | 1591 | AVPR2 | 2088 | IFNGR1 |
| 101 | PSMD3 | 598 | CCL14 | 1095 | PLAUR | 1592 | BMPR1A | 2089 | IFNGR2 |
| 102 | PSMD4 | 599 | CCL4 | 1096 | PLXNA1 | 1593 | BMPR1B | 2090 | IFNA1 |
| 103 | PSMD5 | 600 | CCL16 | 1097 | PLXNA2 | 1594 | BMPR2 | 2091 | IFNA2 |
| 104 | PSMD7 | 601 | CCL19 | 1098 | PLXNA3 | 1595 | BRD8 | 2092 | IFNA4 |
| 105 | PSMD8 | 602 | CCL13 | 1099 | PLXNA4 | 1596 | C3AR1 | 2093 | IFNA5 |
| 106 | PSMD10 | 603 | CCL18 | 1100 | PLXNB1 | 1597 | C5AR1 | 2094 | IFNA6 |
| 107 | PSMD11 | 604 | CCL17 | 1101 | PLXNB2 | 1598 | CALCR | 2095 | IFNA7 |
| 108 | PSMD13 | 605 | CCL26 | 1102 | PLXNB3 | 1599 | CALCRL | 2096 | IFNA8 |
| 109 | PSME1 | 606 | CCL22 | 1103 | PLXNC1 | 1600 | ACKR2 | 2097 | IFNA10 |
| 110 | PSME1 | 607 | CCR3 | 1104 | PLXND1 | 1601 | CCR1 | 2098 | IFNA13 |
| 111 | PSME2 | 608 | CCL28 | 1105 | PTAFR | 1602 | CCR10 | 2099 | IFNA14 |
| 112 | PSME2 | 609 | CCL4L1 | 1106 | ROBO1 | 1603 | CCR3 | 2100 | IFNA16 |
| 113 | RELB | 610 | ACKR2 | 1107 | ROBO2 | 1604 | CCR4 | 2101 | IFNA17 |
| 114 | RFX5 | 611 | CCR7 | 1108 | ROBO3 | 1605 | CCR5 | 2102 | IFNA21 |
| 115 | RFXAP | 612 | CCL27 | 1109 | RXFP3 | 1606 | CCR6 | 2103 | IFNB1 |
| 116 | SLC10A2 | 613 | CCR8 | 1110 | XCR1 | 1607 | CCR7 | 2104 | IFNAR1 |
| 117 | TAP1 | 614 | ACKR4 | 1111 | ADIPOQ | 1608 | CCR8 | 2105 | IFNAR2 |
| 118 | TAP2 | 615 | CCR10 | 1112 | ADM | 1609 | CCR9 | 2106 | TNFSF10 |
| 119 | TAPBP | 616 | CCL2 | 1113 | ADM2 | 1610 | ACKR4 | 2107 | TNFRSF10D |
| 120 | THBS1 | 617 | CCL21 | 1114 | AGRP | 1611 | CCRL2 | 2108 | TNFRSF10C |
| 121 | SEM1 | 618 | CCL7 | 1115 | AGT | 1612 | CD40 | 2109 | TNFRSF10B |
| 122 | KLRC4 | 619 | CCL5 | 1116 | AMBN | 1613 | CMKLR1 | 2110 | TNFRSF10A |
| 123 | AP3B1 | 620 | CCL3 | 1117 | AMELX | 1614 | CNTFR | 2111 | FASLG |
| 124 | RFXANK | 621 | CCL20 | 1118 | AMH | 1615 | CRHR1 | 2112 | FAS |
| 125 | PSMD6 | 622 | CCL11 | 1119 | ANGPTL5 | 1616 | CRHR2 | 2113 | GZMB |
| 126 | PSME3 | 623 | CCR5 | 1120 | ANGPTL7 | 1617 | CRIM1 | 2114 | PRF1 |
| 127 | PSMD14 | 624 | CCL23 | 1121 | APLN | 1618 | CRLF1 | 2115 | CASP3 |
| 128 | CLEC4M | 625 | CCL25 | 1122 | AREG | 1619 | CRLF2 | 2116 | BID |
| 129 | IFI30 | 626 | CCL1 | 1123 | MANF | 1620 | CRLF3 | 2117 | CD3D |
| 130 | PROCR | 627 | CCL3L3 | 1124 | CDNF | 1621 | CSF1R | 2118 | CD3E |
| 131 | ADRM1 | 628 | CCL4L2 | 1125 | ARTN | 1622 | CSF2RA | 2119 | CD3G |
| 132 | ECPAS | 629 | CXCL12 | 1126 | AVP | 1623 | CSF2RB | 2120 | CD247 |
| 133 | TRPC4AP | 630 | XCL1 | 1127 | AZU1 | 1624 | CSF3R | 2121 | CD4 |
| 134 | CD209 | 631 | CCL8 | 1128 | BDNF | 1625 | CX3CR1 | 2122 | CD8A |
| 135 | UBXN1 | 632 | CCL3L1 | 1129 | BMP1 | 1626 | CXCR3 | 2123 | CD8B |
| 136 | ERAP1 | 633 | CCR1 | 1130 | BMP10 | 1627 | CXCR4 | 2124 | PTPRC |
| 137 | TAPBPL | 634 | CCL24 | 1131 | BMP15 | 1628 | CXCR5 | 2125 | LCK |
| 138 | KIR2DL5A | 635 | XCL2 | 1132 | BMP2 | 1629 | CXCR6 | 2126 | FYN |
| 139 | ERAP2 | 636 | CXCL1 | 1133 | BMP3 | 1630 | ACKR3 | 2127 | ZAP70 |
| 140 | ULBP3 | 637 | CXCL10 | 1134 | BMP4 | 1631 | CYSLTR1 | 2128 | LCP2 |
| 141 | ULBP2 | 638 | CXCR4 | 1135 | BMP5 | 1632 | CYSLTR2 | 2129 | LAT |
| 142 | ULBP1 | 639 | CXCL2 | 1136 | BMP6 | 1633 | ACKR1 | 2130 | ITK |
| 143 | KIR3DL3 | 640 | CXCR6 | 1137 | BMP7 | 1634 | EDNRA | 2131 | TEC |
| 144 | RAET1E | 641 | CCR4 | 1138 | BMP8A | 1635 | EDNRB | 2132 | NCK1 |
| 145 | RAET1L | 642 | CXCL11 | 1139 | BMP8B | 1636 | EGFR | 2133 | NCK2 |
| 146 | UBR1 | 643 | TAFA5 | 1140 | BTC | 1637 | ENG | 2134 | VAV3 |
| 147 | RAET1G | 644 | TAFA3 | 1141 | MYDGF | 1638 | EPOR | 2135 | VAV1 |
| 148 | PDIA2 | 645 | TAFA4 | 1142 | C3 | 1639 | ESR1 | 2136 | VAV2 |
| 149 | HAMP | 646 | TAFA1 | 1143 | C5 | 1640 | ESR2 | 2137 | GRAP2 |
| 150 | PI3 | 647 | TAFA2 | 1144 | CALCA | 1641 | ESRRA | 2138 | GRB2 |
| 151 | CAMP | 648 | CCL15-CCL14 | 1145 | CALCB | 1642 | ESRRB | 2139 | PAK1 |
| 152 | DEFB4A | 649 | IL6 | 1146 | CAMP | 1643 | ESRRG | 2140 | PAK2 |
| 153 | PPBP | 650 | TNF | 1147 | CAT | 1644 | FGFR1 | 2141 | PAK3 |
| 154 | REG3G | 651 | IL1B | 1148 | CCK | 1645 | FGFR2 | 2142 | PAK4 |
| 155 | CXCL14 | 652 | IL18 | 1149 | CCL1 | 1646 | FGFR3 | 2143 | PAK6 |
| 156 | CXCL16 | 653 | PTK2B | 1150 | CCL11 | 1647 | FGFR4 | 2144 | PAK5 |
| 157 | SLPI | 654 | VEGFA | 1151 | CCL13 | 1648 | FGFRL1 | 2145 | RHOA |
| 158 | CXCL8 | 655 | IL4 | 1152 | CCL14 | 1649 | FLT1 | 2146 | CDC42 |
| 159 | CXCL10 | 656 | CDH1 | 1153 | CCL15-CCL14 | 1650 | FLT3 | 2147 | PPP3CA |
| 160 | CXCL9 | 657 | CD40 | 1154 | CCL15 | 1651 | FLT4 | 2148 | PPP3CB |
| 161 | CXCL5 | 658 | DEFB103B | 1155 | CCL16 | 1652 | FPR1 | 2149 | PPP3CC |
| 162 | CXCL11 | 659 | F2RL1 | 1156 | CCL17 | 1653 | FPR2 | 2150 | CHP1 |
| 163 | CXCL6 | 660 | MMP9 | 1157 | CCL18 | 1654 | FPR2 | 2151 | PPP3R1 |
| 164 | CXCL1 | 661 | LTBP1 | 1158 | CCL19 | 1655 | FSHR | 2152 | PPP3R2 |
| 165 | CXCL12 | 662 | DEFB4A | 1159 | CCL2 | 1656 | GALR2 | 2153 | CHP2 |
| 166 | CXCL13 | 663 | TNFSF10 | 1160 | CCL20 | 1657 | GALR3 | 2154 | NFAT5 |
| 167 | CXCL2 | 664 | IL13 | 1161 | CCL21 | 1658 | GCGR | 2155 | NFATC1 |
| 168 | PF4 | 665 | IL10 | 1162 | CCL22 | 1659 | GHR | 2156 | NFATC2 |
| 169 | XCL1 | 666 | IL2 | 1163 | CCL23 | 1660 | GHRHR | 2157 | NFATC3 |
| 170 | CXCL3 | 667 | PPARG | 1164 | CCL24 | 1661 | GHSR | 2158 | NFATC4 |
| 171 | DEFB103B | 668 | FGR | 1165 | CCL25 | 1662 | GIPR | 2159 | SOS1 |
| 172 | CCL13 | 669 | MIF | 1166 | CCL26 | 1663 | GLP1R | 2160 | SOS2 |
| 173 | CCL1 | 670 | CRP | 1167 | CCL27 | 1664 | GLP2R | 2161 | HRAS |
| 174 | DEFB1 | 671 | JAK2 | 1168 | CCL28 | 1665 | GNRHR | 2162 | KRAS |
| 175 | CCL8 | 672 | IL1A | 1169 | CCL3 | 1666 | GPER1 | 2163 | NRAS |
| 176 | ELANE | 673 | PTK2 | 1170 | CCL3L1 | 1667 | GPR17 | 2164 | FOS |
| 177 | DEFB103A | 674 | PTGDR | 1171 | CCL3P1 | 1668 | GPR32 | 2165 | JUN |
| 178 | DEFA3 | 675 | CD86 | 1172 | CCL3L3 | 1669 | GPR33 | 2166 | CARD11 |
| 179 | DEFA1 | 676 | HCK | 1173 | CCL4 | 1670 | PTGDR2 | 2167 | BCL10 |
| 180 | TMSB10 | 677 | ARRB1 | 1174 | CCL4L2 | 1671 | C5AR2 | 2168 | MALT1 |
| 181 | DEFA6 | 678 | GNAI1 | 1175 | CCL4L1 | 1672 | HNF4A | 2169 | CHUK |
| 182 | DEFA5 | 679 | VDR | 1176 | CCL5 | 1673 | HNF4G | 2170 | IKBKB |
| 183 | DEFA4 | 680 | OLR1 | 1177 | CCL7 | 1674 | HTR3A | 2171 | IKBKG |
| 184 | LCN2 | 681 | GRK2 | 1178 | CCL8 | 1675 | HTR3B | 2172 | NFKB1 |
| 185 | LCN1 | 682 | TXK | 1179 | CD320 | 1676 | HTR3C | 2173 | RELA |
| 186 | COLEC10 | 683 | RNASE2 | 1180 | CD40LG | 1677 | HTR3D | 2174 | NFKBIA |
| 187 | BPI | 684 | CD79A | 1181 | CD70 | 1678 | HTR3E | 2175 | NFKBIB |
| 188 | S100A9 | 685 | CD79B | 1182 | ADA2 | 1679 | IFNAR1 | 2176 | NFKBIE |
| 189 | S100A8 | 686 | LYN | 1183 | CER1 | 1680 | IFNAR2 | 2177 | CD28 |
| 190 | DCD | 687 | SYK | 1184 | CGA | 1681 | IFNGR1 | 2178 | ICOS |
| 191 | LCN6 | 688 | BTK | 1185 | CGB3 | 1682 | IFNGR2 | 2179 | CD40LG |
| 192 | S100A12 | 689 | BLNK | 1186 | CGB1 | 1683 | IGF1R | 2180 | PIK3R5 |
| 193 | HTN3 | 690 | VAV3 | 1187 | CGB2 | 1684 | IGF2R | 2181 | PIK3R1 |
| 194 | LCN8 | 691 | VAV1 | 1188 | CGB5 | 1685 | IL10RA | 2182 | PIK3R2 |
| 195 | DEFA1B | 692 | VAV2 | 1189 | CGB7 | 1686 | IL10RB | 2183 | PIK3R3 |
| 196 | CCR10 | 693 | RAC1 | 1190 | CGB8 | 1687 | IL11RA | 2184 | PIK3CA |
| 197 | CELA1 | 694 | RAC2 | 1191 | CHGA | 1688 | IL12RB1 | 2185 | PIK3CB |
| 198 | DEFB106A | 695 | RAC3 | 1192 | CHGB | 1689 | IL12RB2 | 2186 | PIK3CD |
| 199 | PENK | 696 | PPP3CA | 1193 | CKLF | 1690 | IL13RA1 | 2187 | PIK3CG |
| 200 | BPIFC | 697 | PPP3CB | 1194 | CLCF1 | 1691 | IL13RA2 | 2188 | AKT3 |
| 201 | MMP12 | 698 | PPP3CC | 1195 | CLEC11A | 1692 | IL15RA | 2189 | AKT1 |
| 202 | BPIFB6 | 699 | CHP1 | 1196 | CMA1 | 1693 | IL2RB | 2190 | AKT2 |
| 203 | LEAP2 | 700 | PPP3R1 | 1197 | CMTM1 | 1694 | IL17RA | 2191 | MAP3K8 |
| 204 | SFTPD | 701 | PPP3R2 | 1198 | CMTM2 | 1695 | IL17RB | 2192 | MAP3K14 |
| 205 | LCN9 | 702 | CHP2 | 1199 | CMTM3 | 1696 | IL17RC | 2193 | PDCD1 |
| 206 | BPIFB2 | 703 | NFAT5 | 1200 | CMTM4 | 1697 | IL17RD | 2194 | CTLA4 |
| 207 | PTGDS | 704 | NFATC1 | 1201 | CMTM5 | 1698 | IL17RE | 2195 | PTPN6 |
| 208 | TMSB4X | 705 | NFATC2 | 1202 | CMTM6 | 1699 | IL18R1 | 2196 | CBLC |
| 209 | PGLYRP1 | 706 | NFATC3 | 1203 | CMTM7 | 1700 | IL18RAP | 2197 | CBL |
| 210 | ZC3HAV1 | 707 | NFATC4 | 1204 | CMTM8 | 1701 | IL1R1 | 2198 | CBLB |
| 211 | TMSB15A | 708 | HRAS | 1205 | CNTF | 1702 | IL1R2 | 2199 | IL2 |
| 212 | S100B | 709 | KRAS | 1206 | CORT | 1703 | IL1RAP | 2200 | IL4 |
| 213 | S100A13 | 710 | NRAS | 1207 | CRH | 1704 | IL1RL1 | 2201 | IL5 |
| 214 | S100A6 | 711 | FOS | 1208 | CSF1 | 1705 | IL1RL2 | 2202 | IL10 |
| 215 | DEFB119 | 712 | JUN | 1209 | CSF2 | 1706 | IL20RA | 2203 | IFNG |
| 216 | DEFB107A | 713 | CARD11 | 1210 | CSF3 | 1707 | IL20RB | 2204 | CSF2 |
| 217 | DEFB105A | 714 | BCL10 | 1211 | CSH1 | 1708 | IL21R | 2205 | TNF |
| 218 | SERPIND1 | 715 | MALT1 | 1212 | CSH2 | 1709 | IL22RA1 | 2206 | CDK4 |
| 219 | DEFB129 | 716 | CHUK | 1213 | CSHL1 | 1710 | IL22RA2 | 2207 | RASGRP1 |
| 220 | DEFB127 | 717 | IKBKB | 1214 | CSPG5 | 1711 | IL23R | 2208 | PDK1 |
| 221 | S100P | 718 | IKBKG | 1215 | CTF1 | 1712 | IL27RA | 2209 | PLCG1 |
| 222 | S100A7 | 719 | NFKB1 | 1216 | CCN2 | 1713 | IFNLR1 | 2210 | PRKCQ |
| 223 | DEFB104A | 720 | RELA | 1217 | CTSG | 1714 | IL2RA | 2211 | TRAC |
| 224 | DEFB126 | 721 | NFKBIA | 1218 | CX3CL1 | 1715 | IL2RB | 2212 | TRAJ1 |
| 225 | DEFB106B | 722 | NFKBIB | 1219 | CXCL1 | 1716 | IL2RG | 2213 | TRAJ2 |
| 226 | DEFB104B | 723 | NFKBIE | 1220 | CXCL10 | 1717 | IL31RA | 2214 | TRAJ3 |
| 227 | DEFB107B | 724 | CD81 | 1221 | CXCL11 | 1718 | IL3RA | 2215 | TRAJ4 |
| 228 | PGLYRP3 | 725 | CD19 | 1222 | CXCL12 | 1719 | IL4R | 2216 | TRAJ5 |
| 229 | PGLYRP2 | 726 | CR2 | 1223 | CXCL13 | 1720 | IL5RA | 2217 | TRAJ6 |
| 230 | S100A10 | 727 | PIK3R5 | 1224 | CXCL14 | 1721 | IL6R | 2218 | TRAJ7 |
| 231 | S100A2 | 728 | PIK3R1 | 1225 | CXCL16 | 1722 | IL7R | 2219 | TRAJ8 |
| 232 | DEFB125 | 729 | PIK3R2 | 1226 | CXCL17 | 1723 | CXCR1 | 2220 | TRAJ9 |
| 233 | DEFB123 | 730 | PIK3R3 | 1227 | CXCL2 | 1724 | CXCR2 | 2221 | TRAJ10 |
| 234 | DEFB105B | 731 | PIK3CA | 1228 | CXCL3 | 1725 | IL9R | 2222 | TRAJ11 |
| 235 | DEFB132 | 732 | PIK3CB | 1229 | CXCL5 | 1726 | INSR | 2223 | TRAJ12 |
| 236 | BPIFB3 | 733 | PIK3CD | 1230 | CXCL6 | 1727 | KDR | 2224 | TRAJ13 |
| 237 | LCN12 | 734 | PIK3CG | 1231 | CXCL9 | 1728 | LEPR | 2225 | TRAJ14 |
| 238 | PGLYRP4 | 735 | AKT3 | 1232 | CCN1 | 1729 | LGR4 | 2226 | TRAJ15 |
| 239 | S100A11 | 736 | AKT1 | 1233 | DEFA1 | 1730 | LGR5 | 2227 | TRAJ16 |
| 240 | S100A5 | 737 | AKT2 | 1234 | DEFA3 | 1731 | LGR6 | 2228 | TRAJ17 |
| 241 | S100A3 | 738 | GSK3B | 1235 | DEFA5 | 1732 | LHCGR | 2229 | TRAJ18 |
| 242 | S100A1 | 739 | INPP5D | 1236 | DEFB1 | 1733 | LIFR | 2230 | TRAJ19 |
| 243 | DEFB128 | 740 | CD22 | 1237 | DEFB103B | 1734 | LTB4R | 2231 | TRAJ20 |
| 244 | DEFB108B | 741 | CD72 | 1238 | DEFB104A | 1735 | LTB4R2 | 2232 | TRAJ21 |
| 245 | HTN1 | 742 | PTPN6 | 1239 | DEFB4A | 1736 | LTBR | 2233 | TRAJ22 |
| 246 | LMBR1L | 743 | LILRB3 | 1240 | DKK1 | 1737 | MC1R | 2234 | TRAJ23 |
| 247 | S100A7A | 744 | FCGR2B | 1241 | EBI3 | 1738 | MC2R | 2235 | TRAJ24 |
| 248 | DEFB118 | 745 | RASGRP3 | 1242 | EDN1 | 1739 | MC3R | 2236 | TRAJ25 |
| 249 | COLEC12 | 746 | PLCG2 | 1243 | EDN2 | 1740 | MC4R | 2237 | TRAJ26 |
| 250 | TMSB4Y | 747 | PRKCB | 1244 | EDN3 | 1741 | MCHR1 | 2238 | TRAJ27 |
| 251 | DEFB131A | 748 | IFITM1 | 1245 | EGF | 1742 | MCHR2 | 2239 | TRAJ28 |
| 252 | DEFB134 | 749 | IGH | 1246 | EPGN | 1743 | MET | 2240 | TRAJ29 |
| 253 | DEFB130A | 750 | IGHA1 | 1247 | EPO | 1744 | MLNR | 2241 | TRAJ30 |
| 254 | DEFB124 | 751 | IGHA2 | 1248 | EREG | 1745 | MPL | 2242 | TRAJ31 |
| 255 | DEFB121 | 752 | IGHD | 1249 | ESM1 | 1746 | MTNR1A | 2243 | TRAJ32 |
| 256 | DEFB116 | 753 | IGHD1-1 | 1250 | FAM3B | 1747 | MTNR1B | 2244 | TRAJ33 |
| 257 | DEFB115 | 754 | IGHD1-14 | 1251 | FAM3C | 1748 | NGFR | 2245 | TRAJ34 |
| 258 | DEFB114 | 755 | IGHD1-20 | 1252 | FAM3D | 1749 | NMBR | 2246 | TRAJ35 |
| 259 | DEFB113 | 756 | IGHD1-26 | 1253 | FASLG | 1750 | NPR1 | 2247 | TRAJ36 |
| 260 | DEFB112 | 757 | IGHD1-7 | 1254 | FGF1 | 1751 | NPR3 | 2248 | TRAJ37 |
| 261 | DEFB110 | 758 | IGHD2-15 | 1255 | FGF10 | 1752 | NR0B1 | 2249 | TRAJ38 |
| 262 | TMSB15B | 759 | IGHD2-2 | 1256 | FGF11 | 1753 | NR0B2 | 2250 | TRAJ39 |
| 263 | DEFB133 | 760 | IGHD2-21 | 1257 | FGF12 | 1754 | NR1D1 | 2251 | TRAJ40 |
| 264 | S100Z | 761 | IGHD2-8 | 1258 | FGF13 | 1755 | NR1D2 | 2252 | TRAJ41 |
| 265 | MAVS | 762 | IGHD3-10 | 1259 | FGF14 | 1756 | NR1H2 | 2253 | TRAJ42 |
| 266 | TMSB4XP8 | 763 | IGHD3-16 | 1260 | FGF16 | 1757 | NR1H3 | 2254 | TRAJ43 |
| 267 | S100A14 | 764 | IGHD3-22 | 1261 | FGF17 | 1758 | NR1H4 | 2255 | TRAJ44 |
| 268 | LCN10 | 765 | IGHD3-3 | 1262 | FGF18 | 1759 | NR1I2 | 2256 | TRAJ45 |
| 269 | S100A16 | 766 | IGHD3-9 | 1263 | FGF19 | 1760 | NR1I3 | 2257 | TRAJ46 |
| 270 | DEFB136 | 767 | IGHD4-11 | 1264 | FGF2 | 1761 | NR2C1 | 2258 | TRAJ47 |
| 271 | DEFB135 | 768 | IGHD4-17 | 1265 | FGF20 | 1762 | NR2C2 | 2259 | TRAJ48 |
| 272 | DEFB117 | 769 | IGHD4-23 | 1266 | FGF21 | 1763 | NR2E1 | 2260 | TRAJ49 |
| 273 | DEFB110 | 770 | IGHD4-4 | 1267 | FGF22 | 1764 | NR2E3 | 2261 | TRAJ50 |
| 274 | ZC3HAV1L | 771 | IGHD5-12 | 1268 | FGF23 | 1765 | NR2F1 | 2262 | TRAJ52 |
| 275 | S100A7L2 | 772 | IGHD5-18 | 1269 | FGF3 | 1766 | NR2F2 | 2263 | TRAJ53 |
| 276 | MBL3P | 773 | IGHD5-24 | 1270 | FGF4 | 1767 | NR2F6 | 2264 | TRAJ54 |
| 277 | DEFB4B | 774 | IGHD5-5 | 1271 | FGF5 | 1768 | NR3C1 | 2265 | TRAJ56 |
| 278 | BPIFB4 | 775 | IGHD6-13 | 1272 | FGF6 | 1769 | NR3C2 | 2266 | TRAJ57 |
| 279 | IFNAR1 | 776 | IGHD6-19 | 1273 | FGF7 | 1770 | NR4A1 | 2267 | TRAJ58 |
| 280 | AZU1 | 777 | IGHD6-25 | 1274 | FGF8 | 1771 | NR4A2 | 2268 | TRAJ59 |
| 281 | DEFB131B | 778 | IGHD6-6 | 1275 | FGF9 | 1772 | NR4A3 | 2269 | TRAJ61 |
| 282 | DEFA1A3 | 779 | IGHD7-27 | 1276 | VEGFD | 1773 | NR5A1 | 2270 | TRAV1-1 |
| 283 | LCN1P1 | 780 | IGHE | 1277 | FIGNL2 | 1774 | NR5A2 | 2271 | TRAV1-2 |
| 284 | S100G | 781 | IGHG1 | 1278 | FLT3LG | 1775 | NR6A1 | 2272 | TRAV2 |
| 285 | DEFA7P | 782 | IGHG2 | 1279 | FSHB | 1776 | NRP1 | 2273 | TRAV3 |
| 286 | DEFB130B | 783 | IGHG3 | 1280 | GAL | 1777 | NRP2 | 2274 | TRAV4 |
| 287 | DEFB108F | 784 | IGHG4 | 1281 | GALP | 1778 | OGFR | 2275 | TRAV5 |
| 288 | DEFB131C | 785 | IGHJ1 | 1282 | GAST | 1779 | OPRD1 | 2276 | TRAV7 |
| 289 | TCHHL1 | 786 | IGHJ2 | 1283 | GCG | 1780 | OPRK1 | 2277 | TRAV8-1 |
| 290 | TINAGL1 | 787 | IGHJ3 | 1284 | GDF1 | 1781 | OPRL1 | 2278 | TRAV8-2 |
| 291 | IFNGR1 | 788 | IGHJ4 | 1285 | GDF10 | 1782 | OPRM1 | 2279 | TRAV8-3 |
| 292 | SLC22A17 | 789 | IGHJ5 | 1286 | GDF11 | 1783 | OSMR | 2280 | TRAV8-4 |
| 293 | WFIKKN1 | 790 | IGHJ6 | 1287 | GDF15 | 1784 | OXTR | 2281 | TRAV8-6 |
| 294 | WFDC2 | 791 | IGHM | 1288 | GDF2 | 1785 | PGR | 2282 | TRAV8-7 |
| 295 | IL6 | 792 | IGH | 1289 | GDF3 | 1786 | PGRMC2 | 2283 | TRAV9-1 |
| 296 | UMODL1 | 793 | IGHV1-18 | 1290 | GDF5 | 1787 | PLAUR | 2284 | TRAV9-2 |
| 297 | TGFB1 | 794 | IGHV1-2 | 1291 | GDF6 | 1788 | PLXNA1 | 2285 | TRAV10 |
| 298 | PF4V1 | 795 | IGHV1-24 | 1292 | GDF7 | 1789 | PLXNA2 | 2286 | TRAV12-1 |
| 299 | MMP9 | 796 | IGHV1-3 | 1293 | GDF9 | 1790 | PLXNA3 | 2287 | TRAV12-2 |
| 300 | ANOS1 | 797 | IGHV1-45 | 1294 | GDNF | 1791 | PLXNA4 | 2288 | TRAV12-3 |
| 301 | TLR4 | 798 | IGHV1-46 | 1295 | GH1 | 1792 | PLXNB1 | 2289 | TRAV13-1 |
| 302 | IFNG | 799 | IGHV1-58 | 1296 | GH2 | 1793 | PLXNB2 | 2290 | TRAV13-2 |
| 303 | SPAG11B | 800 | IGHV1-69 | 1297 | GHRH | 1794 | PLXNB3 | 2291 | TRAV14DV4 |
| 304 | A2M | 801 | IGHV1-8 | 1298 | GHRL | 1795 | PLXNC1 | 2292 | TRAV16 |
| 305 | CTSL | 802 | IGHV1-38-4 | 1299 | GIP | 1796 | PLXND1 | 2293 | TRAV17 |
| 306 | NFKB1 | 803 | IGHV1-69-2 | 1300 | GKN1 | 1797 | PPARA | 2294 | TRAV18 |
| 307 | APOBEC3G | 804 | IGHV2-26 | 1301 | GMFB | 1798 | PPARD | 2295 | TRAV19 |
| 308 | FABP6 | 805 | IGHV2-5 | 1302 | GMFG | 1799 | PPARG | 2296 | TRAV20 |
| 309 | NOD2 | 806 | IGHV2-70 | 1303 | GNRH1 | 1800 | PRLHR | 2297 | TRAV21 |
| 310 | MBL2 | 807 | IGHV3-11 | 1304 | GNRH2 | 1801 | PRLR | 2298 | TRAV22 |
| 311 | SFTPA1 | 808 | IGHV3-13 | 1305 | GPHA2 | 1802 | PTAFR | 2299 | TRAV23DV6 |
| 312 | RBP1 | 809 | IGHV3-15 | 1306 | GPHB5 | 1803 | PTGDR | 2300 | TRAV24 |
| 313 | TLR2 | 810 | IGHV3-16 | 1307 | GPI | 1804 | PTGDS | 2301 | TRAV25 |
| 314 | SLC40A1 | 811 | IGHV3-20 | 1308 | GREM1 | 1805 | PTGER1 | 2302 | TRAV26-1 |
| 315 | PLAU | 812 | IGHV3-21 | 1309 | GREM2 | 1806 | PTGER2 | 2303 | TRAV26-2 |
| 316 | IL1B | 813 | IGHV3-23 | 1310 | GRN | 1807 | PTGER3 | 2304 | TRAV27 |
| 317 | PAEP | 814 | IGHV3-30 | 1311 | GRP | 1808 | PTGER4 | 2305 | TRAV29DV5 |
| 318 | HJV | 815 | IGHV3-30-3 | 1312 | GUCA2A | 1809 | PTGFR | 2306 | TRAV30 |
| 319 | MUC5AC | 816 | IGHV3-30-5 | 1313 | HAMP | 1810 | PTH1R | 2307 | TRAV34 |
| 320 | CTSS | 817 | IGHV3-33 | 1314 | HBEGF | 1811 | PTH2R | 2308 | TRAV35 |
| 321 | OBP2A | 818 | IGHV3-35 | 1315 | HDGF | 1812 | RARA | 2309 | TRAV36DV7 |
| 322 | PLTP | 819 | IGHV3-38 | 1316 | HDGFL3 | 1813 | RARB | 2310 | TRAV38-1 |
| 323 | MX1 | 820 | IGHV3-43 | 1317 | HGF | 1814 | RARG | 2311 | TRAV38-2DV8 |
| 324 | DDX58 | 821 | IGHV3-48 | 1318 | HTN3 | 1815 | ROBO1 | 2312 | TRAV39 |
| 325 | IFNL1 | 822 | IGHV3-49 | 1319 | IAPP | 1816 | ROBO2 | 2313 | TRAV40 |
| 326 | IRF3 | 823 | IGHV3-53 | 1320 | IFNA1 | 1817 | ROBO3 | 2314 | TRAV41 |
| 327 | SFTPA2 | 824 | IGHV3-64 | 1321 | IFNA10 | 1818 | RORA | 2315 | TRBC1 |
| 328 | LPA | 825 | IGHV3-66 | 1322 | IFNA13 | 1819 | RORB | 2316 | TRBC2 |
| 329 | LBP | 826 | IGHV3-7 | 1323 | IFNA14 | 1820 | RORC | 2317 | TRBD1 |
| 330 | RBP4 | 827 | IGHV3-72 | 1324 | IFNA16 | 1821 | RXFP1 | 2318 | TRBD2 |
| 331 | SFTPA1 | 828 | IGHV3-73 | 1325 | IFNA17 | 1822 | RXFP2 | 2319 | TRBJ1-1 |
| 332 | NOX4 | 829 | IGHV3-74 | 1326 | IFNA2 | 1823 | RXFP3 | 2320 | TRBJ1-2 |
| 333 | LTF | 830 | IGHV3-9 | 1327 | IFNA21 | 1824 | RXRA | 2321 | TRBJ1-3 |
| 334 | IFNB1 | 831 | IGHV3-38-3 | 1328 | IFNA4 | 1825 | RXRB | 2322 | TRBJ1-4 |
| 335 | RBP5 | 832 | IGHV3-69-1 | 1329 | IFNA5 | 1826 | RXRG | 2323 | TRBJ1-5 |
| 336 | FABP7 | 833 | IGHV4-28 | 1330 | IFNA6 | 1827 | S1PR1 | 2324 | TRBJ1-6 |
| 337 | FABP5 | 834 | IGHV4-30-1 | 1331 | IFNA7 | 1828 | S1PR2 | 2325 | TRBJ2-1 |
| 338 | FABP3 | 835 | IGHV4-30-2 | 1332 | IFNA8 | 1829 | SCTR | 2326 | TRBJ2-2 |
| 339 | FABP2 | 836 | IGHV4-30-4 | 1333 | IFNB1 | 1830 | SDC1 | 2327 | TRBJ2-3 |
| 340 | FABP4 | 837 | IGHV4-31 | 1334 | IFNE | 1831 | SDC2 | 2328 | TRBJ2-4 |
| 341 | R3HDML | 838 | IGHV4-34 | 1335 | IFNG | 1832 | SDC3 | 2329 | TRBJ2-5 |
| 342 | BPIFA3 | 839 | IGHV4-39 | 1336 | IFNK | 1833 | SDC4 | 2330 | TRBJ2-6 |
| 343 | BPIFB1 | 840 | IGHV4-4 | 1337 | IFNW1 | 1834 | SORT1 | 2331 | TRBJ2-7 |
| 344 | OASL | 841 | IGHV4-59 | 1338 | IGF1 | 1835 | SSTR1 | 2332 | TRBV2 |
| 345 | CRABP2 | 842 | IGHV4-61 | 1339 | IGF2 | 1836 | SSTR2 | 2333 | TRBV3-1 |
| 346 | CRABP1 | 843 | IGHV4-38-2 | 1340 | IL10 | 1837 | SSTR5 | 2334 | TRBV4-1 |
| 347 | RBP7 | 844 | IGHV5-51 | 1341 | IL11 | 1838 | ST2 | 2335 | TRBV4-2 |
| 348 | DUOX1 | 845 | IGHV5-10-1 | 1342 | IL12A | 1839 | TACR1 | 2336 | TRBV4-3 |
| 349 | OBP2B | 846 | IGHV6-1 | 1343 | IL12B | 1840 | TEK | 2337 | TRBV5-1 |
| 350 | RBP2 | 847 | IGHV7-4-1 | 1344 | IL13 | 1841 | TGFBR1 | 2338 | TRBV5-4 |
| 351 | LCN15 | 848 | IGHV7-81 | 1345 | IL15 | 1842 | TGFBR2 | 2339 | TRBV5-5 |
| 352 | CETP | 849 | IGK | 1346 | IL16 | 1843 | TGFBR3 | 2340 | TRBV5-6 |
| 353 | FABP12 | 850 | IGKC | 1347 | IL17A | 1844 | THRA | 2341 | TRBV5-7 |
| 354 | FABP9 | 851 | IGKDEL | 1348 | IL17B | 1845 | THRB | 2342 | TRBV5-8 |
| 355 | BPIFA1 | 852 | IGKJ | 1349 | IL17C | 1846 | TIE1 | 2343 | TRBV6-1 |
| 356 | LCNL1 | 853 | IGKJ1 | 1350 | IL17D | 1847 | TNFRSF10A | 2344 | TRBV6-2 |
| 357 | C8G | 854 | IGKJ2 | 1351 | IL17F | 1848 | TNFRSF10B | 2345 | TRBV6-3 |
| 358 | SPAG11A | 855 | IGKJ3 | 1352 | IL18 | 1849 | TNFRSF10C | 2346 | TRBV6-4 |
| 359 | PI15 | 856 | IGKJ4 | 1353 | IL19 | 1850 | TNFRSF10D | 2347 | TRBV6-5 |
| 360 | NOX1 | 857 | IGKJ5 | 1354 | IL1A | 1851 | TNFRSF11A | 2348 | TRBV6-6 |
| 361 | PMP2 | 858 | IGKV@ | 1355 | IL1B | 1852 | TNFRSF12A | 2349 | TRBV6-7 |
| 362 | APOD | 859 | IGKV1-12 | 1356 | IL1F10 | 1853 | TNFRSF13B | 2350 | TRBV6-8 |
| 363 | ORM2 | 860 | IGKV1-13 | 1357 | IL36RN | 1854 | TNFRSF13C | 2351 | TRBV6-9 |
| 364 | ORM1 | 861 | IGKV1-16 | 1358 | IL36A | 1855 | TNFRSF14 | 2352 | TRBV7-2 |
| 365 | TNF | 862 | IGKV1-17 | 1359 | IL37 | 1856 | TNFRSF17 | 2353 | TRBV7-3 |
| 366 | CTSG | 863 | IGKV1-27 | 1360 | IL36B | 1857 | TNFRSF18 | 2354 | TRBV7-4 |
| 367 | PRTN3 | 864 | IGKV1-33 | 1361 | IL36G | 1858 | TNFRSF19 | 2355 | TRBV7-6 |
| 368 | MAPK1 | 865 | IGKV1-37 | 1362 | IL1RN | 1859 | TNFRSF1A | 2356 | TRBV7-7 |
| 369 | PML | 866 | IGKV1-39 | 1363 | IL2 | 1860 | TNFRSF1B | 2357 | TRBV7-8 |
| 370 | AEN | 867 | IGKV1-5 | 1364 | IL20 | 1861 | TNFRSF21 | 2358 | TRBV7-9 |
| 371 | CYBB | 868 | IGKV1-6 | 1365 | IL21 | 1862 | TNFRSF25 | 2359 | TRBV9 |
| 372 | BPIFA2 | 869 | IGKV1-8 | 1366 | IL22 | 1863 | TNFRSF4 | 2360 | TRBV10-1 |
| 373 | ISG20 | 870 | IGKV1-9 | 1367 | IL23A | 1864 | TNFRSF6B | 2361 | TRBV10-2 |
| 374 | BCL3 | 871 | IGKV1D-12 | 1368 | IL24 | 1865 | TNFRSF8 | 2362 | TRBV10-3 |
| 375 | ISG20L2 | 872 | IGKV1D-13 | 1369 | IL25 | 1866 | TNFRSF9 | 2363 | TRBV11-1 |
| 376 | NOX5 | 873 | IGKV1D-16 | 1370 | IL26 | 1867 | TRHR | 2364 | TRBV11-2 |
| 377 | NOX3 | 874 | IGKV1D-17 | 1371 | IL27 | 1868 | TSHR | 2365 | TRBV11-3 |
| 378 | DUOX2 | 875 | IGKV1D-33 | 1372 | IFNL2 | 1869 | TUBB3 | 2366 | TRBV12-3 |
| 379 | TLR3 | 876 | IGKV1D-37 | 1373 | IFNL3 | 1870 | VDR | 2367 | TRBV12-4 |
| 380 | TFRC | 877 | IGKV1D-39 | 1374 | IFNL1 | 1871 | VIPR1 | 2368 | TRBV12-5 |
| 381 | IFIH1 | 878 | IGKV1D-42 | 1375 | IL3 | 1872 | VIPR2 | 2369 | TRBV13 |
| 382 | LRP1 | 879 | IGKV1D-43 | 1376 | IL31 | 1873 | XCR1 | 2370 | TRBV14 |
| 383 | TRIM5 | 880 | IGKV1D-8 | 1377 | IL32 | 1874 | IFNA10 | 2371 | TRBV15 |
| 384 | IDO1 | 881 | IGKV2-24 | 1378 | IL33 | 1875 | IFNA13 | 2372 | TRBV16 |
| 385 | GDF15 | 882 | IGKV2-28 | 1379 | IL34 | 1876 | IFNA14 | 2373 | TRBV17 |
| 386 | NEDD4 | 883 | IGKV2-30 | 1380 | IL4 | 1877 | IFNA16 | 2374 | TRBV18 |
| 387 | ADIPOQ | 884 | IGKV2-40 | 1381 | IL5 | 1878 | IFNA17 | 2375 | TRBV19 |
| 388 | STAT3 | 885 | IGKV2D-24 | 1382 | IL6 | 1879 | IFNA2 | 2376 | TRBV20-1 |
| 389 | STAT1 | 886 | IGKV2D-28 | 1383 | IL6ST | 1880 | IFNA21 | 2377 | TRBV24-1 |
| 390 | IFNL2 | 887 | IGKV2D-29 | 1384 | IL7 | 1881 | IFNA4 | 2378 | TRBV25-1 |
| 391 | SOCS3 | 888 | IGKV2D-30 | 1385 | CXCL8 | 1882 | IFNA5 | 2379 | TRBV27 |
| 392 | SEMG1 | 889 | IGKV2D-40 | 1386 | IL9 | 1883 | IFNA6 | 2380 | TRBV28 |
| 393 | TNFSF10 | 890 | IGKV3-11 | 1387 | INHA | 1884 | IFNA7 | 2381 | TRBV29-1 |
| 394 | CCL20 | 891 | IGKV3-15 | 1388 | INHBA | 1885 | IFNA8 | 2382 | TRBV30 |
| 395 | SOCS1 | 892 | IGKV3-20 | 1389 | INHBB | 1886 | IFNB1 | 2383 | TRDC |
| 396 | RNASEL | 893 | IGKV3-7 | 1390 | INHBC | 1887 | IFNE | 2384 | TRDD1 |
| 397 | IRF1 | 894 | IGKV3D-11 | 1391 | INHBE | 1888 | IFNG | 2385 | TRDD2 |
| 398 | IL15 | 895 | IGKV3D-15 | 1392 | INS | 1889 | IFNK | 2386 | TRDD3 |
| 399 | APOBEC3F | 896 | IGKV3D-20 | 1393 | INS-IGF2 | 1890 | IFNW1 | 2387 | TRDJ1 |
| 400 | PLAAT4 | 897 | IGKV3D-7 | 1394 | INSL3 | 1891 | IFNAR2 | 2388 | TRDJ2 |
| 401 | CHIT1 | 898 | IGKV4-1 | 1395 | INSL4 | 1892 | IFNGR1 | 2389 | TRDJ3 |
| 402 | IFNA1 | 899 | IGKV5-2 | 1396 | INSL5 | 1893 | IFNGR2 | 2390 | TRDJ4 |
| 403 | CD40 | 900 | IGKV6-21 | 1397 | INSL6 | 1894 | IL11 | 2391 | TRDV1 |
| 404 | TLR7 | 901 | IGKV6D-21 | 1398 | JAG1 | 1895 | IL12A | 2392 | TRDV2 |
| 405 | PPIA | 902 | IGKV6D-41 | 1399 | JAG2 | 1896 | IL12B | 2393 | TRDV3 |
| 406 | HFE | 903 | IGL | 1400 | FGF7P6 | 1897 | IL13 | 2394 | TRGV9 |
| 407 | ZYX | 904 | IGLC1 | 1401 | FGF7P3 | 1898 | IL15 | 2395 | TRGV8 |
| 408 | NLRX1 | 905 | IGLC2 | 1402 | KITLG | 1899 | IL16 | 2396 | TRGV5 |
| 409 | PGC | 906 | IGLC3 | 1403 | KL | 1900 | IL17A | 2397 | TRGV4 |
| 410 | VEGFA | 907 | IGLC6 | 1404 | LACRT | 1901 | IL17B | 2398 | TRGV3 |
| 411 | IKBKE | 908 | IGLC7 | 1405 | LECT2 | 1902 | IL17C | 2399 | TRGV2 |
| 412 | ISG15 | 909 | IGLJ | 1406 | LEFTY1 | 1903 | IL17D | 2400 | TRGJP2 |
| 413 | DHX58 | 910 | IGLJ1 | 1407 | LEFTY2 | 1904 | IL17F | 2401 | TRGJP1 |
| 414 | TNFAIP3 | 911 | IGLJ2 | 1408 | LEP | 1905 | IL18 | 2402 | TRGJP |
| 415 | TFR2 | 912 | IGLJ3 | 1409 | LHB | 1906 | IL19 | 2403 | TRGJ2 |
| 416 | FCN2 | 913 | IGLJ4 | 1410 | LIF | 1907 | IL1A | 2404 | TRGJ1 |
| 417 | MUC4 | 914 | IGLJ5 | 1411 | LRSAM1 | 1908 | IL1B | 2405 | TRGC2 |
| 418 | F2R | 915 | IGLJ6 | 1412 | LTA | 1909 | IL1F10 | 2406 | TRGC1 |
| 419 | ELN | 916 | IGLJ7 | 1413 | LTB | 1910 | IL36RN | 2407 | TRAV6 |
| 420 | IL27 | 917 | IGLV@ | 1414 | LTBP1 | 1911 | IL36A | 2408 | BMP1 |
| 421 | MAPT | 918 | IGLV1-36 | 1415 | LTBP2 | 1912 | IL37 | 2409 | BMP10 |
| 422 | LYZ | 919 | IGLV1-40 | 1416 | LTBP3 | 1913 | IL36B | 2410 | BMP15 |
| 423 | CCL5 | 920 | IGLV1-44 | 1417 | LTBP4 | 1914 | IL36G | 2411 | BMP2 |
| 424 | LEP | 921 | IGLV1-47 | 1418 | MDK | 1915 | IL1RN | 2412 | BMP3 |
| 425 | CYLD | 922 | IGLV1-50 | 1419 | MIA | 1916 | IL2 | 2413 | BMP4 |
| 426 | KLKB1 | 923 | IGLV1-51 | 1420 | MIF | 1917 | IL20 | 2414 | BMP5 |
| 427 | CST4 | 924 | IGLV10-54 | 1421 | MLN | 1918 | IL21 | 2415 | BMP6 |
| 428 | CSRP1 | 925 | IGLV11-55 | 1422 | MSTN | 1919 | IL22 | 2416 | BMP7 |
| 429 | MAPK14 | 926 | IGLV2-11 | 1423 | NAMPT | 1920 | IL23A | 2417 | BMP8A |
| 430 | JUN | 927 | IGLV2-14 | 1424 | NDP | 1921 | IL24 | 2418 | BMP8B |
| 431 | ITGAV | 928 | IGLV2-18 | 1425 | NENF | 1922 | IL25 | 2419 | GDF1 |
| 432 | IRF5 | 929 | IGLV2-23 | 1426 | NGF | 1923 | IL26 | 2420 | GDF10 |
| 433 | CCR6 | 930 | IGLV2-33 | 1427 | NMB | 1924 | IL27 | 2421 | GDF11 |
| 434 | IL12B | 931 | IGLV2-8 | 1428 | NODAL | 1925 | IFNL2 | 2422 | GDF15 |
| 435 | TLR8 | 932 | IGLV3-1 | 1429 | CCN3 | 1926 | IFNL3 | 2423 | GDF2 |
| 436 | GNLY | 933 | IGLV3-10 | 1430 | NPFF | 1927 | IFNL1 | 2424 | GDF3 |
| 437 | CD81 | 934 | IGLV3-12 | 1431 | NPPA | 1928 | IL3 | 2425 | GDF5 |
| 438 | EIF2AK2 | 935 | IGLV3-16 | 1432 | NPPB | 1929 | IL31 | 2426 | GDF6 |
| 439 | APOM | 936 | IGLV3-19 | 1433 | NPPC | 1930 | IL32 | 2427 | GDF7 |
| 440 | CACYBP | 937 | IGLV3-21 | 1434 | NPY | 1931 | IL33 | 2428 | GDF9 |
| 441 | NOD1 | 938 | IGLV3-22 | 1435 | NRG1 | 1932 | IL34 | 2429 | GDNF |
| 442 | MAPK8 | 939 | IGLV3-25 | 1436 | NRG2 | 1933 | IL4 | 2430 | INHA |
| 443 | MAPK3 | 940 | IGLV3-27 | 1437 | NRG3 | 1934 | IL5 | 2431 | INHBA |
| 444 | BST2 | 941 | IGLV3-32 | 1438 | NRG4 | 1935 | IL6 | 2432 | INHBB |
| 445 | BPHL | 942 | IGLV3-9 | 1439 | NRTN | 1936 | IL6ST | 2433 | INHBC |
| 446 | PLA2G2A | 943 | IGLV4-3 | 1440 | NTF3 | 1937 | IL7 | 2434 | INHBE |
| 447 | GRN | 944 | IGLV4-60 | 1441 | NTF4 | 1938 | CXCL8 | 2435 | LEFTY1 |
| 448 | NEWENTRY | 945 | IGLV4-69 | 1442 | NTS | 1939 | IL9 | 2436 | LEFTY2 |
| 449 | PDGFRA | 946 | IGLV5-37 | 1443 | NUDT6 | 1940 | TXLNA | 2437 | NODAL |
| 450 | GNAI1 | 947 | IGLV5-39 | 1444 | OGN | 1941 | IL10RA | 2438 | TGFB1 |
| 451 | WNT5A | 948 | IGLV5-45 | 1445 | OSGIN1 | 1942 | IL10RB | 2439 | TGFB2 |
| 452 | FURIN | 949 | IGLV5-48 | 1446 | OSM | 1943 | IL11RA | 2440 | TGFB3 |
| 453 | ADAR | 950 | IGLV5-52 | 1447 | OSTN | 1944 | IL12RB1 | 2441 | ACVR1B |
| 454 | TYK2 | 951 | IGLV6-57 | 1448 | OXT | 1945 | IL12RB2 | 2442 | ACVR1C |
| 455 | NOS2 | 952 | IGLV7-43 | 1449 | ENDOU | 1946 | IL13RA1 | 2443 | ACVR2A |
| 456 | TRAF3 | 953 | IGLV7-46 | 1450 | PDGFA | 1947 | IL13RA2 | 2444 | ACVR2B |
| 457 | TPT1 | 954 | IGLV8-61 | 1451 | PDGFB | 1948 | IL15RA | 2445 | ACVRL1 |
| 458 | TPM2 | 955 | IGLV9-49 | 1452 | PDGFC | 1949 | IL2RB | 2446 | AMHR2 |
| 459 | NEO1 | 956 | C3 | 1453 | PDGFD | 1950 | IL17RA | 2447 | BMPR1A |
| 460 | AHNAK | 957 | C5 | 1454 | PDGFRA | 1951 | IL17RB | 2448 | BMPR1B |
| 461 | TLR1 | 958 | CAMP | 1455 | PDGFRB | 1952 | IL17RC | 2449 | BMPR2 |
| 462 | TK2 | 959 | CCL1 | 1456 | PDGFRL | 1953 | IL17RD | 2450 | TGFBR1 |
| 463 | PRDX2 | 960 | CCL11 | 1457 | PDYN | 1954 | IL17RE | 2451 | TGFBR2 |
| 464 | MX2 | 961 | CCL13 | 1458 | PENK | 1955 | IL18R1 | 2452 | TGFBR3 |
| 465 | FGF2 | 962 | CCL14 | 1459 | PF4 | 1956 | IL18RAP | 2453 | TNFRSF11B |
| 466 | FGA | 963 | CCL15-CCL14 | 1460 | PF4V1 | 1957 | IL1R1 | 2454 | TNFSF10 |
| 467 | TCF7L2 | 964 | CCL15 | 1461 | PGF | 1958 | IL1R2 | 2455 | TNFSF11 |
| 468 | F2RL1 | 965 | CCL16 | 1462 | PLAU | 1959 | IL1RAP | 2456 | TNFSF12 |
| 469 | TKFC | 966 | CCL17 | 1463 | PMCH | 1960 | IL1RL1 | 2457 | TNFSF13 |
| 470 | MSR1 | 967 | CCL18 | 1464 | PNOC | 1961 | IL1RL2 | 2458 | TNFSF13B |
| 471 | NFKBIZ | 968 | CCL19 | 1465 | POMC | 1962 | IL20RA | 2459 | TNFSF14 |
| 472 | LMBR1 | 969 | CCL2 | 1466 | PPBP | 1963 | IL20RB | 2460 | TNFSF15 |
| 473 | EPPIN | 970 | CCL20 | 1467 | PPBPP1 | 1964 | IL21R | 2461 | TNFSF18 |
| 474 | SRC | 971 | CCL21 | 1468 | PPBPP2 | 1965 | IL22RA1 | 2462 | TNFSF4 |
| 475 | MPO | 972 | CCL22 | 1469 | PPY | 1966 | IL22RA2 | 2463 | TNFSF8 |
| 476 | ELAVL1 | 973 | CCL23 | 1470 | PRL | 1967 | IL23R | 2464 | TNFSF9 |
| 477 | ROBO3 | 974 | CCL24 | 1471 | PRLH | 1968 | IL27RA | 2465 | TNFRSF10B |
| 478 | SP1 | 975 | CCL25 | 1472 | PROK1 | 1969 | IFNLR1 | 2466 | TNFRSF10C |
| 479 | SOD1 | 976 | CCL26 | 1473 | PROK2 | 1970 | IL2RA | 2467 | TNFRSF10D |
| 480 | PDF | 977 | CCL27 | 1474 | PSPN | 1971 | IL2RB | 2468 | TNFRSF11A |
| 481 | DLL4 | 978 | CCL28 | 1475 | PTH | 1972 | IL2RG | 2469 | TNFRSF12A |
| 482 | ECD | 979 | CCL3 | 1476 | PTH2 | 1973 | IL31RA | 2470 | TNFRSF13B |
| 483 | SLC11A1 | 980 | CCL3L1 | 1477 | PTHLH | 1974 | IL3RA | 2471 | TNFRSF13C |
| 484 | DMBT1 | 981 | CCL3P1 | 1478 | PTN | 1975 | IL4R | 2472 | TNFRSF14 |
| 485 | STING1 | 982 | CCL3L3 | 1479 | PYY | 1976 | IL5RA | 2473 | TNFRSF17 |
| 486 | SKIV2L | 983 | CCL4 | 1480 | QRFP | 1977 | IL6R | 2474 | TNFRSF18 |
| 487 | SEMG2 | 984 | CCL4L2 | 1481 | RABEP1 | 1978 | IL7R | 2475 | TNFRSF19 |
| 488 | LTA | 985 | CCL4L1 | 1482 | RABEP2 | 1979 | CXCR1 | 2476 | TNFRSF1A |
| 489 | DES | 986 | CCL5 | 1483 | REG1A | 1980 | CXCR2 | 2477 | TNFRSF1B |
| 490 | DCK | 987 | CCL7 | 1484 | RETN | 1981 | IL9R | 2478 | TNFRSF21 |
| 491 | DAXX | 988 | CCL8 | 1485 | RETNLB | 1982 | ST2 | 2479 | TNFRSF25 |
| 492 | TNFRSF10A | 989 | CKLF | 1486 | RLN1 | 1983 | HLA-A | 2480 | TNFRSF4 |
| 493 | TNFRSF10B | 990 | CMA1 | 1487 | RLN2 | 1984 | HLA-B | 2481 | TNFRSF6B |
| 494 | EED | 991 | CTSG | 1488 | RLN3 | 1985 | HLA-C | 2482 | TNFRSF8 |
| 495 | CCL4 | 992 | CX3CL1 | 1489 | RNASE2 | 1986 | HLA-E | 2483 | TNFRSF9 |
| 496 | LIMS1 | 993 | CXCL1 | 1490 | S100A6 | 1987 | HLA-G |  |  |
| 497 | LALBA | 994 | CXCL10 | 1491 | SAA1 | 1988 | KIR3DL1 |  |  |

Supplementary Table 3. Primer Sequences

| Target Gene | Primer Sequence (5ʹ-3ʹ) |
| --- | --- |
| SLC7A11 | F: TCTCCAAAGGAGGTTACCTGC |
|  | R: AGACTCCCCTCAGTAAAGTGAC |
| DDIT4 | F: TGAGGATGAACACTTGTGTGC |
|  | R: CCAACTGGCTAGGCATCAGC |
| SLC7A5 | F: CCGTGAACTGCTACAGCGT |
|  | R: CTTCCCGATCTGGACGAAGC |
| GDF15 | F: GACCCTCAGAGTTGCACTCC |
|  | R: GCCTGGTTAGCAGGTCCTC |
| IL33 | F: GTGACGGTGTTGATGGTAAGAT |
|  | R: AGCTCCACAGATGTTCCTTG |
| SLC2A1 | F: TCTGGCATCAACGCTGTCTTC |
|  | R: VGATACCGGAGCCAATGGT |
| RRM2 | F: CACGGAGCCGAAAACTAAAGC |
|  | R: TCTGCCTTCTTATACATCTGCCA |
| HLA-DRB5 | F: AGGCAGCATTGAAGTCAGGTG |
|  | R: GAGAGGGCTTGTCACGCTT |
| SFTPD | F: AAGCAGGGGAACATAGGACCT |
|  | R: ACACCTCGCTCTCCCTTAGG |
| PTGDS | F: AGCACCTACTCCGTGTCAGT |
|  | R: TGGGTTCGGCTGTAGAGGG |
| S100B | F: TGGCCCTCATCGACGTTTTC |
|  | R: ATGTTCAAAGAACTCGTGGCA |

Supplementary Table 4. 38 differentially expressed ferroptosis-related genes.

| Gene Symbol | normalMean | tumorMean | logFC | p.Value | FDR |
| --- | --- | --- | --- | --- | --- |
| DUSP1 | 444.6026 | 163.617 | -1.44219 | 2.97E-20 | 1.54E-19 |
| NCF2 | 44.78803 | 15.51373 | -1.52957 | 4.18E-26 | 2.89E-25 |
| ALB | 0.05923 | 32.05455 | 9.079981 | 0.015358 | 0.017462 |
| TXNRD1 | 20.41002 | 67.0796 | 1.716596 | 6.34E-06 | 1.01E-05 |
| SRXN1 | 0.610746 | 1.743401 | 1.513261 | 3.52E-05 | 5.04E-05 |
| GPX2 | 1.210418 | 74.35104 | 5.940775 | 3.05E-07 | 5.16E-07 |
| BNIP3 | 7.409644 | 18.37801 | 1.310504 | 8.79E-21 | 4.86E-20 |
| ANGPTL7 | 1.503572 | 0.16191 | -3.21513 | 2.76E-29 | 3.82E-28 |
| CHAC1 | 1.363953 | 2.800435 | 1.037857 | 2.61E-12 | 6.57E-12 |
| SLC7A11 | 0.542026 | 4.924974 | 3.183683 | 5.74E-17 | 2.65E-16 |
| DDIT4 | 29.70922 | 68.49026 | 1.204988 | 1.17E-07 | 2.02E-07 |
| ASNS | 2.845765 | 10.04325 | 1.819338 | 2.05E-27 | 1.70E-26 |
| JDP2 | 12.08014 | 4.846569 | -1.3176 | 5.66E-24 | 3.36E-23 |
| SLC1A4 | 3.0729 | 7.344181 | 1.257001 | 3.91E-20 | 1.91E-19 |
| GPT2 | 1.069293 | 12.05352 | 3.494725 | 4.27E-32 | 3.55E-30 |
| PSAT1 | 1.117012 | 14.35338 | 3.683673 | 6.69E-29 | 7.93E-28 |
| SLC7A5 | 4.345734 | 31.80058 | 2.871381 | 1.07E-25 | 6.81E-25 |
| ATF3 | 44.12562 | 14.55025 | -1.60057 | 1.58E-13 | 5.03E-13 |
| CBS | 0.068678 | 0.192113 | 1.484028 | 0.000322 | 0.000445 |
| TRIB3 | 4.317575 | 11.66416 | 1.43379 | 2.52E-16 | 1.05E-15 |
| ZFP69B | 0.31234 | 1.146911 | 1.876563 | 4.51E-28 | 4.16E-27 |
| GDF15 | 14.89709 | 47.10163 | 1.660746 | 1.96E-05 | 2.91E-05 |
| IL6 | 27.56645 | 4.304622 | -2.67896 | 5.88E-13 | 1.63E-12 |
| CXCL2 | 117.0396 | 25.89358 | -2.17633 | 6.58E-13 | 1.76E-12 |
| HMOX1 | 81.32346 | 27.95556 | -1.54054 | 9.19E-07 | 1.53E-06 |
| TF | 0.185035 | 6.046326 | 5.030192 | 0.001449 | 0.00185 |
| IL33 | 35.30918 | 8.15549 | -2.1142 | 3.73E-28 | 3.87E-27 |
| HAMP | 0.260147 | 0.624249 | 1.262792 | 2.57E-08 | 4.73E-08 |
| STEAP3 | 9.436297 | 19.0294 | 1.011937 | 1.46E-12 | 3.79E-12 |
| SLC2A1 | 3.179845 | 39.69872 | 3.642064 | 1.64E-29 | 3.82E-28 |
| SLC2A14 | 0.060689 | 0.155092 | 1.353623 | 0.004144 | 0.005134 |
| ALOX5 | 46.25472 | 14.17334 | -1.70642 | 4.96E-27 | 3.75E-26 |
| ALOX15 | 9.771303 | 2.355191 | -2.05271 | 4.25E-12 | 1.04E-11 |
| TFAP2C | 2.885879 | 7.723696 | 1.42028 | 1.90E-15 | 6.87E-15 |
| HBA1 | 3.546271 | 0.181341 | -4.28952 | 1.91E-29 | 3.82E-28 |
| STMN1 | 11.67343 | 26.46883 | 1.181066 | 8.28E-10 | 1.68E-09 |
| RRM2 | 1.120227 | 11.8647 | 3.404813 | 1.17E-29 | 3.82E-28 |
| AURKA | 1.253187 | 8.944746 | 2.835439 | 2.37E-29 | 3.82E-28 |

Supplementary Table 5. 429 differentially expressed immune-related genes.

| Gene Symbol | normalMean | tumorMean | logFC | p.Value | FDR |
| --- | --- | --- | --- | --- | --- |
| CD1A | 1.478375 | 5.071861 | 1.778503 | 0.002698 | 0.003438 |
| FCER1G | 124.5139 | 58.42805 | -1.09157 | 5.99E-20 | 2.90E-19 |
| PDIA3 | 81.46195 | 168.4205 | 1.047869 | 7.49E-23 | 4.81E-22 |
| HLA-DRB5 | 557.5645 | 275.9869 | -1.01454 | 2.63E-13 | 7.51E-13 |
| HLA-E | 727.1101 | 311.3586 | -1.2236 | 1.07E-27 | 1.59E-26 |
| HSPA5 | 125.541 | 257.7451 | 1.037787 | 2.14E-27 | 3.03E-26 |
| HSPA6 | 2.694124 | 7.366297 | 1.451123 | 1.33E-05 | 2.07E-05 |
| IFNG | 0.33131 | 0.908773 | 1.455736 | 0.004785 | 0.005971 |
| KIR2DL3 | 0.133542 | 0.06161 | -1.11605 | 7.00E-16 | 2.55E-15 |
| KIR3DL1 | 0.21319 | 0.067957 | -1.64944 | 1.78E-20 | 9.05E-20 |
| SEM1 | 3.912605 | 8.325003 | 1.089321 | 7.30E-25 | 6.38E-24 |
| CLEC4M | 0.592327 | 0.029607 | -4.32238 | 5.26E-25 | 4.67E-24 |
| PDIA2 | 0.089906 | 2.612437 | 4.860834 | 1.50E-13 | 4.34E-13 |
| HAMP | 0.260147 | 0.624249 | 1.262792 | 2.57E-08 | 4.82E-08 |
| CAMP | 4.195653 | 0.758165 | -2.46831 | 5.57E-27 | 7.39E-26 |
| PPBP | 8.489927 | 2.451079 | -1.79234 | 1.43E-20 | 7.46E-20 |
| CXCL14 | 4.211762 | 88.11883 | 4.386955 | 6.12E-11 | 1.42E-10 |
| SLPI | 1396.085 | 674.5522 | -1.04939 | 1.27E-20 | 6.67E-20 |
| CXCL10 | 17.22468 | 36.94841 | 1.101035 | 0.001239 | 0.001628 |
| CXCL9 | 14.77223 | 41.83907 | 1.501963 | 0.000368 | 0.000506 |
| CXCL12 | 14.63733 | 6.623824 | -1.14392 | 9.86E-20 | 4.75E-19 |
| CXCL13 | 8.6819 | 25.86476 | 1.574905 | 7.90E-17 | 3.07E-16 |
| CXCL2 | 117.0396 | 25.89358 | -2.17633 | 6.58E-13 | 1.82E-12 |
| PF4 | 2.277067 | 0.575505 | -1.98428 | 1.46E-19 | 6.97E-19 |
| CXCL3 | 9.212927 | 3.088262 | -1.57686 | 3.59E-10 | 7.76E-10 |
| CCL1 | 0.0507 | 0.209368 | 2.045982 | 0.000262 | 0.000371 |
| DEFA3 | 0.59001 | 0.059421 | -3.31169 | 2.20E-20 | 1.11E-19 |
| DEFA4 | 0.238865 | 0.044166 | -2.43519 | 3.54E-10 | 7.69E-10 |
| LCN2 | 18.11072 | 118.4225 | 2.709027 | 4.70E-12 | 1.20E-11 |
| COLEC10 | 1.292535 | 0.344735 | -1.90664 | 2.62E-25 | 2.41E-24 |
| LCN6 | 0.36761 | 0.072496 | -2.3422 | 1.31E-21 | 7.58E-21 |
| S100A12 | 5.48208 | 1.495843 | -1.87376 | 7.02E-19 | 3.18E-18 |
| MMP12 | 1.25021 | 21.25016 | 4.087232 | 5.09E-21 | 2.75E-20 |
| SFTPD | 712.9681 | 162.0679 | -2.13724 | 8.91E-27 | 1.11E-25 |
| BPIFB2 | 0.512121 | 12.72517 | 4.635056 | 7.90E-06 | 1.26E-05 |
| PTGDS | 87.65013 | 28.54209 | -1.61867 | 7.16E-23 | 4.63E-22 |
| PGLYRP1 | 0.504042 | 0.143245 | -1.81506 | 3.29E-12 | 8.48E-12 |
| TMSB15A | 3.837132 | 1.345977 | -1.51137 | 6.53E-19 | 2.98E-18 |
| S100B | 2.815052 | 7.719893 | 1.455419 | 0.000361 | 0.000498 |
| S100P | 5.755149 | 191.9468 | 5.05971 | 3.20E-11 | 7.71E-11 |
| S100A7 | 0.105145 | 7.068429 | 6.070932 | 0.000205 | 0.000293 |
| PGLYRP3 | 0.010558 | 0.698497 | 6.047907 | 4.90E-18 | 2.05E-17 |
| PGLYRP2 | 0.018724 | 0.037738 | 1.01108 | 0.00023 | 0.000326 |
| S100A2 | 3.042723 | 32.58212 | 3.420646 | 1.09E-15 | 3.87E-15 |
| LCN12 | 0.153612 | 0.86753 | 2.497625 | 4.93E-15 | 1.66E-14 |
| PGLYRP4 | 0.04988 | 1.121126 | 4.490348 | 1.33E-17 | 5.37E-17 |
| S100A5 | 0.321265 | 1.325968 | 2.04521 | 0.003581 | 0.004496 |
| S100A3 | 9.163536 | 1.892336 | -2.27574 | 1.90E-23 | 1.33E-22 |
| COLEC12 | 18.39124 | 6.260262 | -1.55472 | 3.87E-26 | 4.25E-25 |
| LCN10 | 0.078558 | 0.038903 | -1.01388 | 1.10E-08 | 2.11E-08 |
| S100A16 | 51.44281 | 130.2045 | 1.339738 | 2.93E-12 | 7.65E-12 |
| ZC3HAV1L | 1.020528 | 3.460271 | 1.761569 | 1.67E-24 | 1.43E-23 |
| BPIFB4 | 0.006602 | 0.829389 | 6.972944 | 2.49E-11 | 6.10E-11 |
| DEFB131B | 0.220816 | 0.502217 | 1.185469 | 9.63E-05 | 0.00014 |
| WFDC2 | 75.21713 | 283.8372 | 1.91593 | 9.44E-13 | 2.58E-12 |
| IL6 | 27.56645 | 4.304622 | -2.67896 | 5.88E-13 | 1.64E-12 |
| UMODL1 | 0.050566 | 1.03563 | 4.35621 | 9.14E-13 | 2.50E-12 |
| MMP9 | 8.633327 | 36.96419 | 2.09814 | 7.56E-15 | 2.47E-14 |
| ANOS1 | 31.91014 | 5.039408 | -2.66269 | 2.55E-31 | 2.00E-29 |
| TLR4 | 10.73901 | 4.140559 | -1.37496 | 6.92E-24 | 5.24E-23 |
| A2M | 590.6411 | 175.6471 | -1.7496 | 2.66E-28 | 4.94E-27 |
| SFTPA1 | 6977.927 | 1078.989 | -2.69312 | 1.31E-29 | 3.83E-28 |
| PLAU | 10.34143 | 70.0441 | 2.759828 | 6.58E-19 | 2.99E-18 |
| PAEP | 0.302482 | 63.64531 | 7.71706 | 1.07E-12 | 2.89E-12 |
| HJV | 0.01121 | 0.043775 | 1.965287 | 0.003207 | 0.004047 |
| MUC5AC | 1.736959 | 13.44652 | 2.952598 | 0.002096 | 0.002695 |
| OBP2A | 0.056286 | 0.988767 | 4.134781 | 0.016076 | 0.019127 |
| IFNL1 | 0.024701 | 0.092326 | 1.90214 | 7.85E-05 | 0.000115 |
| SFTPA2 | 7539.332 | 1295.5 | -2.54093 | 4.69E-28 | 7.74E-27 |
| LPA | 0.035795 | 0.015187 | -1.23692 | 2.23E-18 | 9.61E-18 |
| LBP | 0.149306 | 4.621647 | 4.952059 | 0.001229 | 0.001619 |
| RBP4 | 18.45001 | 5.028048 | -1.87555 | 5.07E-25 | 4.54E-24 |
| NOX4 | 0.345453 | 0.954574 | 1.466368 | 1.21E-11 | 3.02E-11 |
| FABP7 | 0.025262 | 1.872002 | 6.211448 | 0.000133 | 0.000192 |
| FABP5 | 25.35651 | 7.718577 | -1.71595 | 1.18E-25 | 1.14E-24 |
| FABP4 | 107.7517 | 3.960279 | -4.76596 | 8.90E-33 | 2.40E-30 |
| CRABP2 | 3.418268 | 186.2009 | 5.767451 | 1.14E-26 | 1.38E-25 |
| CRABP1 | 0.061191 | 4.887019 | 6.319503 | 1.03E-10 | 2.35E-10 |
| DUOX1 | 27.36286 | 4.89136 | -2.48391 | 1.35E-29 | 3.84E-28 |
| RBP2 | 3.996103 | 0.624809 | -2.67711 | 2.37E-28 | 4.56E-27 |
| CETP | 1.927188 | 0.859384 | -1.16512 | 3.20E-17 | 1.28E-16 |
| FABP12 | 0.115673 | 0.037066 | -1.64189 | 4.76E-17 | 1.87E-16 |
| BPIFA1 | 10.95098 | 467.747 | 5.416597 | 5.31E-05 | 7.86E-05 |
| C8G | 0.330086 | 1.111537 | 1.751642 | 3.47E-05 | 5.19E-05 |
| PI15 | 0.274563 | 1.933612 | 2.816089 | 5.66E-11 | 1.32E-10 |
| NOX1 | 0.303717 | 0.882442 | 1.538776 | 9.17E-16 | 3.29E-15 |
| CTSG | 3.698904 | 1.021836 | -1.85593 | 1.89E-14 | 5.97E-14 |
| CYBB | 63.76422 | 28.89203 | -1.14208 | 1.04E-15 | 3.70E-15 |
| BPIFA2 | 0.107993 | 4.890765 | 5.501053 | 4.38E-11 | 1.04E-10 |
| NOX5 | 0.017323 | 0.177079 | 3.353592 | 1.56E-12 | 4.15E-12 |
| GDF15 | 14.89709 | 47.10163 | 1.660746 | 1.96E-05 | 3.00E-05 |
| SOCS3 | 153.4327 | 60.31969 | -1.3469 | 1.08E-07 | 1.94E-07 |
| CCL20 | 10.88857 | 29.14343 | 1.420356 | 6.00E-05 | 8.85E-05 |
| IKBKE | 2.344303 | 5.701858 | 1.282273 | 7.45E-24 | 5.60E-23 |
| ISG15 | 34.43242 | 107.6985 | 1.645159 | 8.01E-10 | 1.69E-09 |
| TFR2 | 0.085765 | 0.814056 | 3.246663 | 1.61E-30 | 7.16E-29 |
| MUC4 | 1.186956 | 6.924349 | 2.544412 | 7.30E-09 | 1.42E-08 |
| CST4 | 0.009968 | 1.389262 | 7.122829 | 2.92E-19 | 1.37E-18 |
| ITGAV | 12.7496 | 27.68125 | 1.118457 | 4.94E-14 | 1.50E-13 |
| IL12B | 0.122286 | 0.272069 | 1.153715 | 0.001267 | 0.001662 |
| TLR8 | 5.792703 | 1.942092 | -1.57663 | 2.58E-22 | 1.59E-21 |
| BPHL | 1.423359 | 3.808754 | 1.420019 | 4.37E-27 | 5.88E-26 |
| FURIN | 40.21692 | 86.72655 | 1.108671 | 0.008375 | 0.01025 |
| AHNAK | 122.6824 | 35.40462 | -1.79292 | 9.51E-26 | 9.62E-25 |
| MX2 | 1.679919 | 5.15473 | 1.617505 | 2.16E-19 | 1.02E-18 |
| FGF2 | 2.574237 | 0.700082 | -1.87855 | 6.46E-27 | 8.36E-26 |
| F2RL1 | 4.091854 | 10.90665 | 1.414381 | 8.36E-11 | 1.92E-10 |
| MSR1 | 37.7082 | 8.555869 | -2.13989 | 1.99E-28 | 4.02E-27 |
| EPPIN | 0.115102 | 0.04355 | -1.40216 | 1.48E-08 | 2.83E-08 |
| PDF | 0.693571 | 1.612843 | 1.217491 | 3.29E-21 | 1.81E-20 |
| SLC11A1 | 13.1425 | 3.962841 | -1.72963 | 2.38E-24 | 1.96E-23 |
| DES | 36.89823 | 4.6449 | -2.98983 | 3.10E-30 | 1.22E-28 |
| TMPRSS6 | 0.675629 | 3.591892 | 2.410441 | 0.028643 | 0.033152 |
| MARCO | 213.6366 | 28.35032 | -2.91372 | 8.89E-31 | 5.05E-29 |
| TNFSF11 | 0.053846 | 0.978892 | 4.184234 | 1.19E-23 | 8.54E-23 |
| JUND | 183.7335 | 73.41771 | -1.32341 | 3.02E-20 | 1.50E-19 |
| CLDN4 | 40.68778 | 101.9 | 1.324486 | 4.11E-19 | 1.89E-18 |
| CCL28 | 1.522662 | 3.332576 | 1.130042 | 0.003581 | 0.004496 |
| IRF7 | 8.281184 | 21.37489 | 1.368008 | 2.41E-21 | 1.36E-20 |
| IL17A | 0.007138 | 0.041568 | 2.541799 | 0.00015 | 0.000215 |
| APOBEC3A | 1.816199 | 0.81468 | -1.15662 | 1.45E-11 | 3.58E-11 |
| IL7R | 38.18495 | 10.28555 | -1.89239 | 1.84E-23 | 1.29E-22 |
| PTX3 | 5.654385 | 1.530073 | -1.88577 | 2.79E-13 | 7.95E-13 |
| MASP1 | 1.258358 | 0.208669 | -2.59226 | 5.42E-30 | 1.79E-28 |
| PROC | 0.116082 | 1.907603 | 4.038543 | 3.63E-22 | 2.18E-21 |
| HRG | 0.00208 | 0.07684 | 5.207268 | 2.74E-09 | 5.51E-09 |
| HMOX1 | 81.32346 | 27.95556 | -1.54054 | 9.19E-07 | 1.57E-06 |
| ARRB1 | 22.24055 | 10.29667 | -1.11101 | 7.10E-25 | 6.26E-24 |
| STAB2 | 0.35926 | 0.163622 | -1.13466 | 2.52E-09 | 5.09E-09 |
| PDYN | 0.018347 | 0.006648 | -1.46455 | 9.18E-05 | 0.000134 |
| PDCD1 | 0.953591 | 2.074048 | 1.121007 | 2.18E-06 | 3.62E-06 |
| PCSK1 | 0.102226 | 10.61905 | 6.698748 | 7.99E-09 | 1.54E-08 |
| ARG2 | 1.24401 | 3.05455 | 1.295961 | 3.84E-09 | 7.57E-09 |
| AQP9 | 12.05712 | 4.581112 | -1.39612 | 6.45E-18 | 2.64E-17 |
| BIRC5 | 0.820054 | 11.80302 | 3.847293 | 6.39E-30 | 2.04E-28 |
| VIM | 320.4624 | 154.8834 | -1.04897 | 1.40E-25 | 1.34E-24 |
| GFAP | 0.017747 | 0.170332 | 3.262664 | 9.40E-16 | 3.36E-15 |
| ALB | 0.05923 | 32.05455 | 9.079981 | 0.015358 | 0.018358 |
| OAS1 | 8.197085 | 20.21066 | 1.301933 | 1.15E-10 | 2.59E-10 |
| AGER | 1120.071 | 35.52935 | -4.97844 | 3.08E-33 | 1.57E-30 |
| TNFSF4 | 0.526542 | 1.391529 | 1.40205 | 1.40E-12 | 3.74E-12 |
| NOS1 | 0.915885 | 0.2658 | -1.78483 | 2.65E-25 | 2.42E-24 |
| CXCR1 | 2.614492 | 0.382055 | -2.77468 | 5.32E-23 | 3.53E-22 |
| CCL14 | 2.145932 | 0.418934 | -2.35681 | 3.62E-23 | 2.45E-22 |
| CCL16 | 0.133442 | 0.055881 | -1.25579 | 6.64E-09 | 1.29E-08 |
| CCL19 | 14.49633 | 32.44598 | 1.162352 | 2.31E-06 | 3.82E-06 |
| CCL18 | 167.8241 | 82.63528 | -1.02212 | 7.38E-06 | 1.18E-05 |
| CCL26 | 0.34467 | 0.887998 | 1.365342 | 0.000216 | 0.000307 |
| CCL22 | 2.71189 | 5.544523 | 1.031764 | 0.002125 | 0.002728 |
| CCR3 | 0.104107 | 0.304624 | 1.548966 | 2.99E-08 | 5.59E-08 |
| CCR8 | 0.202394 | 0.691552 | 1.772674 | 5.91E-15 | 1.98E-14 |
| ACKR4 | 2.721182 | 0.544599 | -2.32097 | 7.34E-28 | 1.12E-26 |
| CCL2 | 62.46581 | 24.09782 | -1.37416 | 1.67E-06 | 2.79E-06 |
| CCL7 | 0.348485 | 1.395245 | 2.001352 | 1.34E-09 | 2.75E-09 |
| CCL23 | 8.161916 | 1.564325 | -2.38337 | 2.91E-28 | 5.30E-27 |
| CCL25 | 0.034537 | 0.302952 | 3.132886 | 7.21E-13 | 1.98E-12 |
| CCL24 | 11.45074 | 2.31579 | -2.30586 | 1.18E-13 | 3.47E-13 |
| TAFA3 | 0.028268 | 0.123784 | 2.130606 | 2.94E-05 | 4.42E-05 |
| CCL15-CCL14 | 0.190716 | 0.06784 | -1.49122 | 2.29E-14 | 7.12E-14 |
| CDH1 | 35.14423 | 79.79412 | 1.182995 | 4.59E-20 | 2.26E-19 |
| IL13 | 0.08957 | 0.027714 | -1.69243 | 1.87E-06 | 3.12E-06 |
| PPARG | 12.57589 | 4.575455 | -1.45867 | 8.47E-24 | 6.32E-23 |
| FGR | 25.8864 | 6.45257 | -2.00425 | 4.32E-30 | 1.55E-28 |
| MIF | 17.81028 | 44.55402 | 1.322846 | 4.76E-19 | 2.18E-18 |
| HCK | 27.235 | 12.18452 | -1.16041 | 1.63E-21 | 9.43E-21 |
| OLR1 | 62.29078 | 12.04446 | -2.37065 | 4.55E-29 | 1.06E-27 |
| CD79A | 7.8141 | 22.37414 | 1.517681 | 2.90E-12 | 7.57E-12 |
| BTK | 7.635804 | 3.230653 | -1.24095 | 1.50E-23 | 1.07E-22 |
| RAC3 | 1.875082 | 10.52677 | 2.489036 | 1.19E-23 | 8.54E-23 |
| PPP3R2 | 0.009205 | 0.00395 | -1.22069 | 6.92E-11 | 1.60E-10 |
| NFATC1 | 3.947911 | 1.958811 | -1.01111 | 8.33E-17 | 3.23E-16 |
| NFATC4 | 1.975681 | 4.957471 | 1.327254 | 4.58E-16 | 1.68E-15 |
| FOS | 292.177 | 103.6393 | -1.49527 | 2.42E-12 | 6.37E-12 |
| CARD11 | 1.967008 | 7.4519 | 1.921605 | 1.82E-15 | 6.35E-15 |
| CD19 | 0.777729 | 1.837927 | 1.24074 | 4.82E-13 | 1.35E-12 |
| PIK3R5 | 4.42361 | 2.106898 | -1.0701 | 2.21E-18 | 9.59E-18 |
| LILRB3 | 3.05953 | 1.280856 | -1.2562 | 9.36E-23 | 5.91E-22 |
| IFITM1 | 56.60105 | 130.4889 | 1.205026 | 5.66E-08 | 1.04E-07 |
| CMA1 | 0.820643 | 0.243788 | -1.75113 | 1.34E-13 | 3.91E-13 |
| CCN1 | 218.9357 | 78.95409 | -1.47142 | 1.13E-16 | 4.34E-16 |
| EDN1 | 48.42873 | 13.23203 | -1.87183 | 2.65E-21 | 1.49E-20 |
| EDN2 | 1.16585 | 4.731606 | 2.020947 | 4.44E-08 | 8.19E-08 |
| EDN3 | 0.540643 | 0.085785 | -2.65588 | 5.95E-27 | 7.80E-26 |
| FGF10 | 0.984094 | 0.127715 | -2.94587 | 2.60E-29 | 6.80E-28 |
| PROK2 | 0.722528 | 0.186645 | -1.95276 | 2.42E-14 | 7.49E-14 |
| SAA1 | 4.98024 | 26.40458 | 2.406501 | 0.000338 | 0.000468 |
| SAA2 | 0.67935 | 3.665327 | 2.431715 | 2.64E-05 | 3.98E-05 |
| SEMA3A | 0.773772 | 2.956698 | 1.934007 | 0.000303 | 0.000421 |
| SEMA3B | 19.20601 | 6.722344 | -1.51452 | 3.63E-22 | 2.18E-21 |
| SEMA3D | 1.095959 | 0.53228 | -1.04194 | 2.66E-16 | 9.96E-16 |
| SEMA3G | 15.77786 | 1.774756 | -3.15221 | 2.74E-30 | 1.12E-28 |
| SEMA4B | 9.47956 | 31.33917 | 1.725075 | 6.91E-23 | 4.50E-22 |
| SEMA4C | 5.893571 | 11.9851 | 1.024028 | 5.77E-18 | 2.41E-17 |
| SEMA5A | 10.75815 | 2.073769 | -2.3751 | 2.01E-28 | 4.02E-27 |
| SEMA6A | 4.655482 | 0.86705 | -2.42474 | 1.10E-28 | 2.39E-27 |
| SEMA6D | 2.792664 | 0.773627 | -1.85193 | 3.51E-24 | 2.78E-23 |
| SEMA7A | 2.449724 | 5.104993 | 1.05929 | 8.25E-05 | 0.00012 |
| SLIT2 | 12.74012 | 2.553428 | -2.31887 | 7.69E-30 | 2.38E-28 |
| TNC | 11.02095 | 38.56456 | 1.807027 | 1.82E-06 | 3.05E-06 |
| C5AR1 | 32.43466 | 9.236383 | -1.81214 | 8.61E-28 | 1.29E-26 |
| CCRL2 | 7.215452 | 2.590087 | -1.47809 | 5.79E-26 | 6.16E-25 |
| CX3CR1 | 3.957878 | 1.423259 | -1.47553 | 3.18E-16 | 1.19E-15 |
| ACKR1 | 30.13205 | 6.059605 | -2.314 | 3.54E-24 | 2.78E-23 |
| EDNRB | 35.36461 | 3.382163 | -3.38629 | 2.20E-32 | 2.90E-30 |
| FPR1 | 19.58376 | 5.767473 | -1.76365 | 2.62E-19 | 1.24E-18 |
| FPR2 | 6.389966 | 0.953004 | -2.74525 | 3.26E-27 | 4.56E-26 |
| GPR17 | 0.985315 | 0.2568 | -1.93994 | 2.08E-22 | 1.29E-21 |
| CXCR2 | 3.087709 | 0.555968 | -2.47346 | 2.17E-26 | 2.44E-25 |
| PLXNA3 | 2.942397 | 6.566949 | 1.158232 | 1.22E-14 | 3.91E-14 |
| PLXNB3 | 0.315159 | 3.506216 | 3.475765 | 1.60E-20 | 8.24E-20 |
| ROBO2 | 1.397081 | 0.481514 | -1.53677 | 1.75E-21 | 1.00E-20 |
| ADM2 | 0.366086 | 4.300122 | 3.554122 | 2.54E-30 | 1.08E-28 |
| AGRP | 4.823451 | 0.646297 | -2.8998 | 2.26E-28 | 4.45E-27 |
| AGT | 1.667335 | 16.1211 | 3.273334 | 1.60E-07 | 2.85E-07 |
| AMH | 0.152588 | 0.538771 | 1.820032 | 0.042173 | 0.048108 |
| ANGPTL5 | 0.371542 | 0.070126 | -2.40551 | 2.10E-24 | 1.74E-23 |
| ANGPTL7 | 1.503572 | 0.16191 | -3.21513 | 2.76E-29 | 7.05E-28 |
| APLN | 12.66982 | 4.307941 | -1.55633 | 5.00E-15 | 1.68E-14 |
| MANF | 15.38756 | 43.13139 | 1.486973 | 2.45E-29 | 6.58E-28 |
| ARTN | 0.149816 | 1.082796 | 2.853498 | 3.09E-18 | 1.31E-17 |
| BDNF | 1.406972 | 0.31297 | -2.1685 | 1.05E-25 | 1.04E-24 |
| BMP15 | 0.043805 | 0.009962 | -2.13664 | 8.58E-09 | 1.65E-08 |
| BMP2 | 22.5957 | 7.399704 | -1.61051 | 2.03E-21 | 1.15E-20 |
| BMP8A | 0.162129 | 0.91984 | 2.504239 | 2.67E-22 | 1.63E-21 |
| BMP8B | 0.701842 | 1.82793 | 1.380993 | 7.21E-17 | 2.81E-16 |
| CALCB | 0.017153 | 0.331623 | 4.272985 | 0.000269 | 0.000378 |
| CAT | 100.1085 | 29.37069 | -1.76912 | 1.58E-32 | 2.69E-30 |
| CD70 | 0.33715 | 0.887711 | 1.396699 | 2.60E-05 | 3.93E-05 |
| CGA | 0.012746 | 5.284687 | 8.695576 | 5.96E-06 | 9.66E-06 |
| CGB7 | 0.046731 | 0.138553 | 1.567988 | 2.84E-06 | 4.68E-06 |
| CHGB | 0.140563 | 7.509505 | 5.73943 | 0.001454 | 0.0019 |
| CLCF1 | 5.127929 | 10.52859 | 1.037864 | 1.21E-10 | 2.73E-10 |
| CMTM1 | 0.373632 | 0.9231 | 1.304869 | 7.55E-19 | 3.40E-18 |
| CMTM2 | 1.511289 | 0.325615 | -2.21454 | 6.94E-26 | 7.24E-25 |
| CMTM5 | 0.183317 | 0.024462 | -2.9057 | 4.38E-30 | 1.55E-28 |
| CORT | 0.109265 | 0.221023 | 1.016368 | 1.02E-07 | 1.85E-07 |
| CSF3 | 29.69327 | 1.240552 | -4.58108 | 1.46E-16 | 5.56E-16 |
| CCN2 | 233.0455 | 85.76436 | -1.44216 | 2.50E-18 | 1.07E-17 |
| EGF | 0.336018 | 1.832699 | 2.447361 | 8.15E-14 | 2.43E-13 |
| EPO | 0.02193 | 0.058229 | 1.408843 | 0.016572 | 0.019648 |
| FAM3C | 8.519867 | 22.42759 | 1.396372 | 1.45E-20 | 7.52E-20 |
| FGF11 | 0.022455 | 0.23451 | 3.384536 | 3.35E-24 | 2.72E-23 |
| FGF13 | 0.211361 | 0.448337 | 1.084873 | 0.01632 | 0.019395 |
| FGF14 | 0.588698 | 0.273336 | -1.10685 | 1.62E-14 | 5.15E-14 |
| FGF19 | 0.001357 | 1.097355 | 9.65966 | 2.10E-14 | 6.61E-14 |
| FGF20 | 0.030095 | 0.197999 | 2.717914 | 6.03E-06 | 9.75E-06 |
| FGF5 | 0.002342 | 0.145897 | 5.96098 | 7.76E-09 | 1.50E-08 |
| VEGFD | 25.48636 | 2.31982 | -3.45764 | 1.29E-31 | 1.20E-29 |
| FIGNL2 | 0.13065 | 0.330296 | 1.338058 | 0.024419 | 0.028554 |
| GAL | 0.069196 | 2.934659 | 5.406354 | 3.79E-16 | 1.40E-15 |
| GDF10 | 11.65102 | 1.370375 | -3.08781 | 1.25E-30 | 6.01E-29 |
| GDF2 | 0.040849 | 0.002072 | -4.30104 | 1.45E-34 | 1.48E-31 |
| GDNF | 0.02764 | 0.25275 | 3.192872 | 1.32E-12 | 3.55E-12 |
| GMFG | 39.93228 | 15.52367 | -1.36309 | 1.81E-26 | 2.05E-25 |
| GPI | 17.91683 | 42.96202 | 1.261746 | 1.06E-26 | 1.31E-25 |
| GREM1 | 0.137989 | 4.824581 | 5.127774 | 2.79E-26 | 3.10E-25 |
| HBEGF | 49.49853 | 9.092514 | -2.44463 | 3.77E-27 | 5.21E-26 |
| HDGF | 50.96271 | 114.4974 | 1.167801 | 7.85E-27 | 9.90E-26 |
| IFNE | 0.023163 | 0.207531 | 3.163413 | 0.002741 | 0.003485 |
| IL11 | 0.098387 | 0.726419 | 2.884262 | 1.03E-18 | 4.58E-18 |
| IL17C | 0.031791 | 0.676563 | 4.411512 | 6.41E-15 | 2.13E-14 |
| IL17D | 1.208075 | 0.431212 | -1.48624 | 1.74E-26 | 2.00E-25 |
| IL19 | 0.008982 | 0.073018 | 3.02316 | 9.43E-05 | 0.000137 |
| IL36RN | 0.013675 | 1.140841 | 6.382394 | 3.99E-19 | 1.86E-18 |
| IL37 | 0.132428 | 13.09671 | 6.627853 | 7.14E-13 | 1.97E-12 |
| IL36G | 0.022987 | 0.536043 | 4.543442 | 7.55E-16 | 2.74E-15 |
| IL20 | 0.078317 | 0.035567 | -1.13878 | 0.000767 | 0.001025 |
| IL21 | 0.034654 | 0.099865 | 1.526967 | 4.32E-06 | 7.08E-06 |
| IL23A | 0.549734 | 2.685277 | 2.288266 | 3.68E-23 | 2.48E-22 |
| IL33 | 35.30918 | 8.15549 | -2.1142 | 3.73E-28 | 6.36E-27 |
| IL34 | 4.688876 | 2.054584 | -1.1904 | 5.05E-21 | 2.74E-20 |
| INHA | 0.087956 | 7.321084 | 6.379139 | 6.72E-14 | 2.02E-13 |
| INHBB | 4.454593 | 10.2656 | 1.204452 | 0.000276 | 0.000388 |
| INHBC | 0.02475 | 0.063755 | 1.365135 | 0.000552 | 0.000743 |
| INHBE | 0.069126 | 0.340306 | 2.299538 | 0.000725 | 0.00097 |
| INSL3 | 0.115417 | 0.502961 | 2.123585 | 3.17E-21 | 1.75E-20 |
| INSL4 | 0.008076 | 2.35017 | 8.184922 | 7.61E-06 | 1.22E-05 |
| KL | 3.792707 | 0.790593 | -2.26222 | 4.42E-29 | 1.05E-27 |
| LEFTY2 | 1.595551 | 0.443722 | -1.84633 | 4.85E-22 | 2.86E-21 |
| LHB | 0.096058 | 0.44948 | 2.226277 | 1.78E-14 | 5.62E-14 |
| LTBP4 | 53.07472 | 12.34211 | -2.10444 | 3.13E-29 | 7.79E-28 |
| MDK | 17.06061 | 156.1424 | 3.19412 | 1.44E-27 | 2.07E-26 |
| MIA | 0.072147 | 0.676101 | 3.228235 | 0.017813 | 0.021046 |
| NDP | 0.078427 | 0.857148 | 3.450126 | 0.000965 | 0.001285 |
| NPPC | 0.081694 | 0.177502 | 1.119534 | 0.011319 | 0.013707 |
| NRG1 | 1.01632 | 0.384775 | -1.40127 | 4.84E-24 | 3.78E-23 |
| NRG3 | 0.395238 | 0.067178 | -2.55667 | 5.56E-28 | 9.01E-27 |
| OGN | 9.178151 | 2.249434 | -2.02864 | 2.54E-23 | 1.73E-22 |
| OSTN | 0.065396 | 0.026329 | -1.31254 | 1.61E-19 | 7.67E-19 |
| OXT | 0.082031 | 0.400982 | 2.289295 | 0.000103 | 0.000149 |
| ENDOU | 0.233084 | 0.088054 | -1.40439 | 1.84E-21 | 1.05E-20 |
| PDGFB | 16.65215 | 6.447132 | -1.36898 | 3.37E-20 | 1.67E-19 |
| PDGFRL | 2.418613 | 7.418172 | 1.616884 | 2.36E-16 | 8.90E-16 |
| PGF | 0.823307 | 2.350999 | 1.513772 | 1.26E-13 | 3.70E-13 |
| PMCH | 0.043162 | 0.150534 | 1.802251 | 9.75E-11 | 2.22E-10 |
| PNOC | 0.293806 | 0.883825 | 1.588895 | 2.75E-11 | 6.70E-11 |
| PRLH | 0.286997 | 0.063399 | -2.17851 | 3.01E-09 | 6.01E-09 |
| PTHLH | 0.308564 | 2.181486 | 2.821669 | 1.08E-09 | 2.24E-09 |
| REG1A | 0.022978 | 4.550825 | 7.629714 | 0.035781 | 0.041088 |
| RETN | 28.16904 | 3.75112 | -2.90872 | 6.98E-28 | 1.08E-26 |
| RETNLB | 0.003007 | 0.110166 | 5.195255 | 3.57E-09 | 7.08E-09 |
| RLN3 | 0.070253 | 0.487539 | 2.794878 | 8.50E-08 | 1.54E-07 |
| SCG2 | 0.866321 | 9.72614 | 3.488894 | 0.012427 | 0.014995 |
| SCT | 0.60311 | 1.295754 | 1.103299 | 4.46E-05 | 6.63E-05 |
| SPP1 | 9.711535 | 292.0983 | 4.910611 | 3.12E-28 | 5.59E-27 |
| TGFA | 3.026673 | 9.042915 | 1.579055 | 4.76E-12 | 1.21E-11 |
| THPO | 0.155861 | 1.311992 | 3.073423 | 8.23E-06 | 1.30E-05 |
| TNFSF12 | 29.97493 | 13.99772 | -1.09856 | 9.15E-29 | 2.03E-27 |
| TNFSF13 | 20.27482 | 9.501594 | -1.09345 | 4.16E-27 | 5.67E-26 |
| TRH | 0.258162 | 0.107406 | -1.2652 | 0.000299 | 0.000417 |
| TSHB | 0.073777 | 0.02622 | -1.49249 | 2.67E-09 | 5.38E-09 |
| TSLP | 0.505153 | 0.156145 | -1.69384 | 9.62E-24 | 7.07E-23 |
| UCN | 0.545546 | 1.555178 | 1.511308 | 9.88E-11 | 2.25E-10 |
| UCN2 | 0.020774 | 0.360725 | 4.118068 | 2.14E-16 | 8.08E-16 |
| UTS2 | 0.116742 | 0.275318 | 1.237769 | 0.034769 | 0.040016 |
| VGF | 0.036752 | 2.636217 | 6.164511 | 1.37E-20 | 7.15E-20 |
| VIP | 0.329757 | 0.11641 | -1.50218 | 2.13E-15 | 7.37E-15 |
| ACVRL1 | 36.98182 | 6.668024 | -2.47148 | 4.32E-32 | 4.91E-30 |
| ADCYAP1R1 | 0.451672 | 0.110109 | -2.03635 | 2.24E-23 | 1.54E-22 |
| ADRB1 | 11.26419 | 1.357459 | -3.05276 | 4.59E-30 | 1.56E-28 |
| ADRB2 | 12.85155 | 1.681538 | -2.93409 | 9.40E-33 | 2.40E-30 |
| AGTR1 | 2.30331 | 0.706592 | -1.70476 | 3.98E-26 | 4.33E-25 |
| AGTR2 | 12.94788 | 2.627775 | -2.3008 | 1.88E-24 | 1.58E-23 |
| ANGPT1 | 7.524705 | 1.715454 | -2.13304 | 2.63E-28 | 4.94E-27 |
| ANGPT4 | 1.600741 | 0.092208 | -4.1177 | 5.63E-31 | 3.60E-29 |
| ANGPTL1 | 3.905903 | 0.943746 | -2.04919 | 6.58E-28 | 1.04E-26 |
| ANGPTL3 | 0.007906 | 0.177468 | 4.48841 | 4.52E-12 | 1.16E-11 |
| ANGPTL4 | 8.43318 | 24.51499 | 1.539516 | 4.49E-07 | 7.82E-07 |
| APLNR | 10.06196 | 3.84699 | -1.38711 | 4.76E-10 | 1.01E-09 |
| AVPR1B | 0.006088 | 0.0648 | 3.411856 | 5.55E-07 | 9.61E-07 |
| AVPR2 | 1.23126 | 0.356908 | -1.78651 | 2.15E-25 | 2.02E-24 |
| BMPR2 | 21.8914 | 9.528568 | -1.20003 | 3.38E-24 | 2.72E-23 |
| CALCRL | 34.93392 | 5.591914 | -2.64321 | 2.89E-31 | 2.11E-29 |
| CNTFR | 1.340754 | 0.278152 | -2.2691 | 9.35E-26 | 9.56E-25 |
| CRHR1 | 0.007629 | 0.104255 | 3.772438 | 0.000266 | 0.000376 |
| CRIM1 | 28.98943 | 12.5047 | -1.21306 | 1.21E-24 | 1.05E-23 |
| CRLF1 | 2.713411 | 48.46844 | 4.158866 | 9.37E-06 | 1.48E-05 |
| CRLF2 | 0.192635 | 0.546034 | 1.503121 | 0.035004 | 0.040241 |
| ENG | 109.0457 | 38.34384 | -1.50787 | 6.59E-27 | 8.42E-26 |
| FGFR2 | 11.07779 | 3.582015 | -1.62883 | 1.05E-25 | 1.04E-24 |
| FGFR4 | 16.16333 | 3.061205 | -2.40055 | 7.04E-29 | 1.60E-27 |
| FLT4 | 5.601574 | 2.359157 | -1.24756 | 1.02E-19 | 4.89E-19 |
| FSHR | 0.012195 | 0.004399 | -1.47095 | 1.37E-09 | 2.80E-09 |
| GALR2 | 0.055691 | 0.293604 | 2.398347 | 1.11E-09 | 2.30E-09 |
| GALR3 | 0.003958 | 0.051861 | 3.71174 | 8.69E-07 | 1.49E-06 |
| GCGR | 0.005742 | 0.080415 | 3.807859 | 4.99E-12 | 1.26E-11 |
| GHR | 1.399297 | 0.384977 | -1.86186 | 3.21E-28 | 5.66E-27 |
| GNRHR | 0.034515 | 0.188609 | 2.450124 | 6.54E-05 | 9.62E-05 |
| GPER1 | 4.903078 | 1.120605 | -2.12941 | 3.23E-25 | 2.92E-24 |
| HNF4G | 0.160254 | 2.02852 | 3.661996 | 6.52E-24 | 4.98E-23 |
| HTR3A | 0.055795 | 3.570794 | 5.999968 | 1.09E-25 | 1.07E-24 |
| HTR3C | 1.848308 | 0.082369 | -4.48796 | 1.80E-31 | 1.53E-29 |
| IL12RB2 | 0.122094 | 0.381431 | 1.643429 | 5.30E-06 | 8.61E-06 |
| IL17RD | 0.349648 | 0.94591 | 1.435799 | 2.92E-11 | 7.09E-11 |
| IL18R1 | 3.503218 | 1.342496 | -1.38376 | 2.35E-14 | 7.30E-14 |
| IL1RL1 | 9.922415 | 1.491551 | -2.73388 | 1.92E-23 | 1.34E-22 |
| IL1RL2 | 0.261527 | 1.421004 | 2.441879 | 1.75E-24 | 1.48E-23 |
| IL20RA | 5.907246 | 1.944368 | -1.60318 | 1.19E-20 | 6.32E-20 |
| IL20RB | 0.277458 | 4.297452 | 3.953139 | 6.98E-16 | 2.55E-15 |
| IL22RA1 | 0.541148 | 1.410714 | 1.382331 | 0.025763 | 0.029988 |
| IL22RA2 | 0.03245 | 0.598447 | 4.204913 | 5.71E-23 | 3.76E-22 |
| IL2RA | 1.249014 | 3.659566 | 1.550883 | 6.71E-13 | 1.85E-12 |
| IL31RA | 0.047719 | 0.93054 | 4.285432 | 1.30E-14 | 4.18E-14 |
| IL3RA | 19.54266 | 6.55744 | -1.57542 | 1.42E-29 | 3.93E-28 |
| IL5RA | 1.37388 | 0.33151 | -2.05113 | 1.65E-20 | 8.41E-20 |
| LEPR | 6.573552 | 1.951796 | -1.75187 | 1.09E-27 | 1.59E-26 |
| LGR4 | 1.729219 | 12.18902 | 2.81739 | 1.23E-30 | 6.01E-29 |
| LHCGR | 0.011357 | 0.08543 | 2.911148 | 0.028288 | 0.032778 |
| LIFR | 17.54679 | 6.535095 | -1.42493 | 4.18E-22 | 2.50E-21 |
| MC1R | 0.459412 | 1.108808 | 1.27115 | 1.01E-13 | 2.98E-13 |
| MC4R | 0.009012 | 0.075036 | 3.057683 | 3.55E-10 | 7.69E-10 |
| MCHR1 | 0.071815 | 0.332728 | 2.21198 | 1.09E-15 | 3.87E-15 |
| MET | 16.60897 | 48.0633 | 1.532973 | 3.85E-05 | 5.75E-05 |
| MLNR | 0.023129 | 0.067093 | 1.536451 | 0.003269 | 0.00412 |
| MTNR1A | 0.009806 | 0.171895 | 4.131759 | 4.66E-12 | 1.19E-11 |
| NMBR | 0.069918 | 0.030615 | -1.19144 | 3.28E-12 | 8.48E-12 |
| NPR1 | 15.98451 | 2.84166 | -2.49187 | 1.29E-30 | 6.01E-29 |
| NPR3 | 7.165678 | 2.177759 | -1.71826 | 3.09E-21 | 1.73E-20 |
| NR1I2 | 0.038571 | 0.227772 | 2.562015 | 0.022811 | 0.026797 |
| NR1I3 | 0.090341 | 0.187074 | 1.050153 | 1.12E-08 | 2.13E-08 |
| NR2E1 | 0.00376 | 0.104318 | 4.794231 | 5.36E-14 | 1.62E-13 |
| NR2F6 | 11.22092 | 23.83098 | 1.086647 | 1.14E-20 | 6.15E-20 |
| NR4A1 | 51.61213 | 17.19693 | -1.58556 | 1.15E-14 | 3.70E-14 |
| NR4A3 | 10.51635 | 2.479258 | -2.08465 | 1.61E-15 | 5.63E-15 |
| NR5A1 | 0.00613 | 0.076493 | 3.64148 | 1.00E-06 | 1.71E-06 |
| NR5A2 | 1.18947 | 0.575599 | -1.04718 | 1.08E-08 | 2.06E-08 |
| OPRD1 | 0.043504 | 0.256178 | 2.55794 | 1.21E-05 | 1.89E-05 |
| OSMR | 16.42034 | 34.28193 | 1.061965 | 3.11E-12 | 8.08E-12 |
| PGR | 0.571578 | 0.191917 | -1.57447 | 5.31E-24 | 4.08E-23 |
| PTGER4 | 10.28822 | 3.883394 | -1.4056 | 1.30E-26 | 1.56E-25 |
| PTH1R | 3.25431 | 0.917245 | -1.82697 | 4.16E-28 | 6.98E-27 |
| RXFP1 | 1.84606 | 0.232397 | -2.98979 | 3.72E-31 | 2.53E-29 |
| RXFP2 | 0.204878 | 0.034257 | -2.5803 | 1.45E-26 | 1.73E-25 |
| RXRG | 2.498992 | 0.545313 | -2.19619 | 4.41E-26 | 4.75E-25 |
| S1PR1 | 52.55097 | 7.907103 | -2.7325 | 2.27E-32 | 2.90E-30 |
| SSTR1 | 4.754159 | 1.043228 | -2.18814 | 6.37E-26 | 6.71E-25 |
| SSTR2 | 0.169979 | 0.366052 | 1.106692 | 1.03E-05 | 1.62E-05 |
| TEK | 21.62083 | 2.449167 | -3.14206 | 1.42E-32 | 2.69E-30 |
| TGFBR2 | 115.9618 | 39.50553 | -1.55352 | 9.11E-32 | 9.31E-30 |
| TGFBR3 | 10.80377 | 1.93277 | -2.48279 | 8.89E-31 | 5.05E-29 |
| TIE1 | 14.80812 | 4.029716 | -1.87764 | 1.01E-29 | 3.05E-28 |
| TNFRSF11A | 0.493682 | 1.266439 | 1.359125 | 4.55E-12 | 1.16E-11 |
| TNFRSF17 | 0.979781 | 3.872548 | 1.982752 | 2.80E-11 | 6.80E-11 |
| TNFRSF18 | 0.973393 | 6.601404 | 2.761679 | 1.01E-21 | 5.95E-21 |
| TNFRSF21 | 13.86349 | 59.68186 | 2.106002 | 1.97E-28 | 4.02E-27 |
| TNFRSF25 | 1.026244 | 4.031503 | 1.973944 | 2.08E-18 | 9.03E-18 |
| TNFRSF9 | 0.457801 | 1.343434 | 1.553134 | 1.50E-10 | 3.34E-10 |
| TSHR | 0.0263 | 0.256125 | 3.283709 | 8.64E-16 | 3.11E-15 |
| TUBB3 | 0.07057 | 2.100897 | 4.895801 | 1.29E-30 | 6.01E-29 |
| VIPR1 | 20.39881 | 2.275887 | -3.16398 | 3.59E-28 | 6.21E-27 |
| ICAM2 | 9.175839 | 3.648665 | -1.33047 | 1.55E-26 | 1.82E-25 |
| ITGAL | 13.57449 | 6.51129 | -1.05988 | 6.20E-18 | 2.58E-17 |
| PAK1 | 5.919229 | 14.7881 | 1.320956 | 4.24E-29 | 1.03E-27 |
| NCR2 | 0.010148 | 0.035087 | 1.78971 | 2.04E-06 | 3.39E-06 |
| TYROBP | 229.8088 | 88.81406 | -1.37157 | 8.48E-23 | 5.42E-22 |
| FCGR3A | 82.79638 | 39.7959 | -1.05695 | 1.85E-17 | 7.42E-17 |
| FCGR3B | 4.17588 | 0.903162 | -2.20902 | 1.67E-17 | 6.73E-17 |
| SHC3 | 3.100682 | 1.015979 | -1.60971 | 9.44E-23 | 5.92E-22 |
| CD244 | 1.769258 | 0.786532 | -1.16957 | 6.78E-16 | 2.48E-15 |
| PRKCG | 0.020098 | 0.116159 | 2.530959 | 1.09E-09 | 2.25E-09 |
| SH2D1B | 0.933643 | 0.296462 | -1.65502 | 3.12E-21 | 1.73E-20 |
| PRF1 | 15.22979 | 7.015996 | -1.11818 | 8.06E-15 | 2.62E-14 |
| PAK6 | 0.042712 | 0.108661 | 1.347122 | 2.27E-18 | 9.74E-18 |
| PAK5 | 0.072372 | 0.028564 | -1.34121 | 6.44E-18 | 2.64E-17 |
| CTLA4 | 0.952502 | 2.220242 | 1.220923 | 2.81E-09 | 5.64E-09 |
| CBLC | 1.329719 | 13.34192 | 3.326773 | 6.45E-28 | 1.03E-26 |
| CDK4 | 13.33031 | 29.05022 | 1.123838 | 1.15E-20 | 6.17E-20 |
| PDK1 | 0.668499 | 2.519559 | 1.914174 | 1.50E-28 | 3.19E-27 |
| PRKCQ | 4.196596 | 1.59792 | -1.39302 | 1.22E-24 | 1.05E-23 |

Supplementary Table 6. 16 prognostic ferroptosis-related genes.

| Gene Symbol | HR | HR.95L | HR.95H | p.value |
| --- | --- | --- | --- | --- |
| TXNRD1 | 1.107308 | 1.00359 | 1.221745 | 0.042217 |
| SLC7A11 | 1.122546 | 1.009317 | 1.248477 | 0.033096 |
| DDIT4 | 1.245213 | 1.098306 | 1.41177 | 0.000617 |
| SLC7A5 | 1.166857 | 1.035506 | 1.314869 | 0.011323 |
| HERPUD1 | 0.673754 | 0.533084 | 0.851544 | 0.00095 |
| SLC3A2 | 1.308638 | 1.03619 | 1.652723 | 0.023918 |
| GDF15 | 0.88156 | 0.80272 | 0.968144 | 0.008358 |
| CEBPG | 1.290664 | 1.028015 | 1.620416 | 0.027951 |
| EIF2S1 | 1.605106 | 1.170827 | 2.200467 | 0.003285 |
| RELA | 1.623309 | 1.047179 | 2.516409 | 0.030305 |
| IL33 | 0.844471 | 0.740684 | 0.962801 | 0.011519 |
| SLC2A1 | 1.241203 | 1.114576 | 1.382216 | 8.29E-05 |
| RRM2 | 1.310606 | 1.150304 | 1.493247 | 4.83E-05 |
| HNF4A | 1.286286 | 1.099714 | 1.50451 | 0.00164 |
| YWHAE | 1.392976 | 1.01249 | 1.916444 | 0.041728 |
| AURKA | 1.25187 | 1.085651 | 1.443539 | 0.001998 |

Supplementary Table 7. 51 prognostic immune-related genes.

| Gene Symbol | HR | HR.95L | HR.95H | p.value |
| --- | --- | --- | --- | --- |
| CD1B | 0.725311 | 0.572463 | 0.91897 | 0.007818 |
| CD1C | 0.82741 | 0.724054 | 0.945521 | 0.005389 |
| CD1D | 0.668428 | 0.511522 | 0.873465 | 0.003167 |
| CD1E | 0.793869 | 0.673397 | 0.935894 | 0.005979 |
| CD74 | 0.866668 | 0.779123 | 0.964049 | 0.008442 |
| CTSL | 1.236816 | 1.074955 | 1.423048 | 0.002978 |
| FCGRT | 0.699609 | 0.574076 | 0.852592 | 0.000399 |
| HLA-DMA | 0.803843 | 0.709134 | 0.911201 | 0.00064 |
| HLA-DMB | 0.826733 | 0.730096 | 0.936162 | 0.002699 |
| HLA-DOA | 0.854544 | 0.762195 | 0.958082 | 0.007063 |
| HLA-DOB | 0.786441 | 0.670539 | 0.922377 | 0.003144 |
| HLA-DPA1 | 0.868696 | 0.783029 | 0.963735 | 0.007877 |
| HLA-DPB1 | 0.861362 | 0.77365 | 0.959017 | 0.006456 |
| HLA-DQA1 | 0.857139 | 0.77349 | 0.949834 | 0.003258 |
| HLA-DQB1 | 0.875952 | 0.796716 | 0.963069 | 0.006184 |
| HLA-DRA | 0.864753 | 0.78017 | 0.958507 | 0.005659 |
| HLA-DRB1 | 0.86814 | 0.784456 | 0.960752 | 0.006254 |
| HLA-DRB5 | 0.88014 | 0.807998 | 0.958723 | 0.003433 |
| MR1 | 0.753621 | 0.598249 | 0.949344 | 0.016339 |
| HSPA1A | 1.187805 | 1.014975 | 1.390064 | 0.031934 |
| HSPA1B | 1.202423 | 1.005289 | 1.438215 | 0.043622 |
| HSPA2 | 1.226386 | 1.049366 | 1.433269 | 0.010294 |
| HSPA4 | 1.772043 | 1.240942 | 2.530446 | 0.001646 |
| HSP90AA1 | 1.330516 | 1.03182 | 1.71568 | 0.027705 |
| IFNA5 | 0.000979 | 1.21E-06 | 0.79036 | 0.042474 |
| KIR2DL1 | 0.296691 | 0.098545 | 0.893254 | 0.030718 |
| KIR3DL2 | 0.330353 | 0.124484 | 0.876682 | 0.026131 |
| KLRC2 | 1.444938 | 1.03316 | 2.020834 | 0.03151 |
| CIITA | 0.775208 | 0.654988 | 0.917493 | 0.003061 |
| PSMC1 | 1.420083 | 1.062895 | 1.897306 | 0.017663 |
| PSMC4 | 1.376867 | 1.05311 | 1.800155 | 0.01937 |
| PSMC5 | 1.563703 | 1.071074 | 2.282912 | 0.02058 |
| PSMC6 | 1.370898 | 1.032979 | 1.819362 | 0.028914 |
| PSMD1 | 1.384036 | 1.072621 | 1.785865 | 0.012454 |
| PSMD2 | 1.599241 | 1.242726 | 2.058034 | 0.000264 |
| PSMD11 | 1.525516 | 1.157054 | 2.011314 | 0.002752 |
| RELB | 1.248925 | 1.000605 | 1.55887 | 0.049378 |
| RFX5 | 0.658086 | 0.48829 | 0.886928 | 0.005995 |
| RFXAP | 0.493373 | 0.330376 | 0.736785 | 0.000555 |
| TAP2 | 1.252589 | 1.030891 | 1.521965 | 0.023446 |
| PSME3 | 1.432986 | 1.044032 | 1.966843 | 0.02597 |
| RAET1E | 1.669825 | 1.284948 | 2.169984 | 0.000125 |
| RAET1L | 1.458565 | 1.111548 | 1.913917 | 0.006472 |
| RAET1G | 1.43095 | 1.123818 | 1.82202 | 0.00365 |
| CXCL5 | 1.098642 | 1.005795 | 1.200058 | 0.036776 |
| CCL13 | 0.891635 | 0.808142 | 0.983754 | 0.022226 |
| DEFB1 | 1.083322 | 1.009122 | 1.162977 | 0.02705 |
| SFTPD | 0.901772 | 0.84803 | 0.958921 | 0.000974 |
| PTGDS | 0.847129 | 0.761486 | 0.942405 | 0.002282 |
| TMSB4X | 0.828697 | 0.688893 | 0.996873 | 0.046241 |
| S100B | 0.887115 | 0.793216 | 0.992131 | 0.035873 |

Supplementary Table 8. Gene ontology (GO) analyses of DEGs between the high-risk and low-risk groups.

| ONTOLOGY | ID | Description | GeneRatio | BgRatio | pvalue |
| --- | --- | --- | --- | --- | --- |
| BP | GO:0140014 | mitotic nuclear division | 53/511 | 264/18670 | 1.06E-30 |
| BP | GO:0007059 | chromosome segregation | 53/511 | 321/18670 | 2.35E-26 |
| BP | GO:0000280 | nuclear division | 59/511 | 407/18670 | 3.53E-26 |
| BP | GO:0000070 | mitotic sister chromatid segregation | 38/511 | 151/18670 | 4.54E-26 |
| BP | GO:0048285 | organelle fission | 61/511 | 449/18670 | 1.69E-25 |
| BP | GO:0000819 | sister chromatid segregation | 39/511 | 189/18670 | 2.70E-23 |
| BP | GO:0098813 | nuclear chromosome segregation | 43/511 | 262/18670 | 1.91E-21 |
| BP | GO:0007088 | regulation of mitotic nuclear division | 31/511 | 164/18670 | 1.35E-17 |
| BP | GO:0051983 | regulation of chromosome segregation | 24/511 | 103/18670 | 4.39E-16 |
| BP | GO:0051783 | regulation of nuclear division | 31/511 | 188/18670 | 7.65E-16 |
| BP | GO:1902850 | microtubule cytoskeleton organization involved in mitosis | 26/511 | 131/18670 | 1.68E-15 |
| BP | GO:0007091 | metaphase/anaphase transition of mitotic cell cycle | 17/511 | 54/18670 | 3.86E-14 |
| BP | GO:0044784 | metaphase/anaphase transition of cell cycle | 17/511 | 56/18670 | 7.63E-14 |
| BP | GO:0010965 | regulation of mitotic sister chromatid separation | 17/511 | 57/18670 | 1.06E-13 |
| BP | GO:0030071 | regulation of mitotic metaphase/anaphase transition | 16/511 | 51/18670 | 2.33E-13 |
| BP | GO:0051306 | mitotic sister chromatid separation | 17/511 | 60/18670 | 2.72E-13 |
| BP | GO:0051304 | chromosome separation | 20/511 | 90/18670 | 3.33E-13 |
| BP | GO:1902099 | regulation of metaphase/anaphase transition of cell cycle | 16/511 | 53/18670 | 4.59E-13 |
| BP | GO:1905818 | regulation of chromosome separation | 17/511 | 62/18670 | 4.95E-13 |
| BP | GO:0008608 | attachment of spindle microtubules to kinetochore | 13/511 | 32/18670 | 8.98E-13 |
| BP | GO:0007052 | mitotic spindle organization | 21/511 | 106/18670 | 9.34E-13 |
| BP | GO:0007051 | spindle organization | 26/511 | 170/18670 | 1.04E-12 |
| BP | GO:0033047 | regulation of mitotic sister chromatid segregation | 17/511 | 68/18670 | 2.58E-12 |
| BP | GO:0007093 | mitotic cell cycle checkpoint | 25/511 | 165/18670 | 3.55E-12 |
| BP | GO:0033045 | regulation of sister chromatid segregation | 18/511 | 80/18670 | 4.08E-12 |
| BP | GO:0000075 | cell cycle checkpoint | 27/511 | 216/18670 | 4.83E-11 |
| BP | GO:0007094 | mitotic spindle assembly checkpoint | 12/511 | 34/18670 | 4.93E-11 |
| BP | GO:0031577 | spindle checkpoint | 12/511 | 34/18670 | 4.93E-11 |
| BP | GO:0071173 | spindle assembly checkpoint | 12/511 | 34/18670 | 4.93E-11 |
| BP | GO:0071174 | mitotic spindle checkpoint | 12/511 | 34/18670 | 4.93E-11 |
| BP | GO:0033046 | negative regulation of sister chromatid segregation | 13/511 | 44/18670 | 9.94E-11 |
| BP | GO:0045841 | negative regulation of mitotic metaphase/anaphase transition | 12/511 | 36/18670 | 1.07E-10 |
| BP | GO:0051985 | negative regulation of chromosome segregation | 13/511 | 45/18670 | 1.36E-10 |
| BP | GO:1902100 | negative regulation of metaphase/ anaphase transition of cell cycle | 12/511 | 37/18670 | 1.55E-10 |
| BP | GO:0090068 | positive regulation of cell cycle process | 31/511 | 298/18670 | 2.03E-10 |
| BP | GO:2000816 | negative regulation of mitotic sister chromatid separation | 12/511 | 39/18670 | 3.10E-10 |
| BP | GO:1901987 | regulation of cell cycle phase transition | 40/511 | 480/18670 | 4.29E-10 |
| BP | GO:1905819 | negative regulation of chromosome separation | 12/511 | 40/18670 | 4.32E-10 |
| BP | GO:0071103 | DNA conformation change | 34/511 | 364/18670 | 4.83E-10 |
| BP | GO:0033048 | negative regulation of mitotic sister chromatid segregation | 12/511 | 42/18670 | 8.14E-10 |
| BP | GO:0045839 | negative regulation of mitotic nuclear division | 13/511 | 52/18670 | 9.95E-10 |
| BP | GO:0044839 | cell cycle G2/M phase transition | 28/511 | 266/18670 | 1.19E-09 |
| BP | GO:1901990 | regulation of mitotic cell cycle phase transition | 37/511 | 444/18670 | 1.98E-09 |
| BP | GO:0045787 | positive regulation of cell cycle | 34/511 | 389/18670 | 2.72E-09 |
| BP | GO:0051310 | metaphase plate congression | 13/511 | 57/18670 | 3.39E-09 |
| BP | GO:0000086 | G2/M transition of mitotic cell cycle | 26/511 | 247/18670 | 4.68E-09 |
| BP | GO:0006323 | DNA packaging | 26/511 | 247/18670 | 4.68E-09 |
| BP | GO:0019730 | antimicrobial humoral response | 18/511 | 122/18670 | 5.95E-09 |
| BP | GO:0051784 | negative regulation of nuclear division | 13/511 | 60/18670 | 6.63E-09 |
| BP | GO:0051302 | regulation of cell division | 21/511 | 168/18670 | 6.97E-09 |
| BP | GO:0051303 | establishment of chromosome localization | 14/511 | 75/18670 | 1.35E-08 |
| BP | GO:0050000 | chromosome localization | 14/511 | 76/18670 | 1.61E-08 |
| BP | GO:2001251 | negative regulation of chromosome organization | 19/511 | 146/18670 | 1.88E-08 |
| BP | GO:0033044 | regulation of chromosome organization | 30/511 | 342/18670 | 2.20E-08 |
| BP | GO:1901988 | negative regulation of cell cycle phase transition | 26/511 | 267/18670 | 2.37E-08 |
| BP | GO:0090307 | mitotic spindle assembly | 12/511 | 56/18670 | 2.90E-08 |
| BP | GO:0051225 | spindle assembly | 16/511 | 108/18670 | 3.93E-08 |
| BP | GO:0051383 | kinetochore organization | 8/511 | 21/18670 | 4.43E-08 |
| BP | GO:0071459 | protein localization to chromosome, centromeric region | 8/511 | 21/18670 | 4.43E-08 |
| BP | GO:0045930 | negative regulation of mitotic cell cycle | 29/511 | 338/18670 | 6.13E-08 |
| BP | GO:0000281 | mitotic cytokinesis | 13/511 | 72/18670 | 6.74E-08 |
| BP | GO:0010948 | negative regulation of cell cycle process | 30/511 | 361/18670 | 7.37E-08 |
| BP | GO:1901991 | negative regulation of mitotic cell cycle phase transition | 24/511 | 248/18670 | 9.26E-08 |
| BP | GO:0051321 | meiotic cell cycle | 24/511 | 249/18670 | 9.99E-08 |
| BP | GO:1902749 | regulation of cell cycle G2/M phase transition | 22/511 | 213/18670 | 1.01E-07 |
| BP | GO:0045931 | positive regulation of mitotic cell cycle | 19/511 | 163/18670 | 1.13E-07 |
| BP | GO:0043129 | surfactant homeostasis | 6/511 | 11/18670 | 1.68E-07 |
| BP | GO:0034501 | protein localization to kinetochore | 7/511 | 17/18670 | 1.69E-07 |
| BP | GO:0007080 | mitotic metaphase plate congression | 10/511 | 44/18670 | 2.32E-07 |
| BP | GO:1903046 | meiotic cell cycle process | 20/511 | 188/18670 | 2.39E-07 |
| BP | GO:0034508 | centromere complex assembly | 11/511 | 56/18670 | 2.84E-07 |
| BP | GO:0019731 | antibacterial humoral response | 10/511 | 46/18670 | 3.63E-07 |
| BP | GO:0030261 | chromosome condensation | 10/511 | 47/18670 | 4.50E-07 |
| BP | GO:0010389 | regulation of G2/M transition of mitotic cell cycle | 20/511 | 196/18670 | 4.69E-07 |
| BP | GO:0006334 | nucleosome assembly | 17/511 | 145/18670 | 4.83E-07 |
| BP | GO:0061640 | cytoskeleton-dependent cytokinesis | 14/511 | 100/18670 | 5.67E-07 |
| BP | GO:0048875 | chemical homeostasis within a tissue | 6/511 | 13/18670 | 5.95E-07 |
| BP | GO:0016572 | histone phosphorylation | 9/511 | 38/18670 | 6.46E-07 |
| BP | GO:0007568 | aging | 26/511 | 321/18670 | 8.98E-07 |
| BP | GO:0000910 | cytokinesis | 18/511 | 171/18670 | 1.11E-06 |
| BP | GO:0140013 | meiotic nuclear division | 18/511 | 172/18670 | 1.21E-06 |
| BP | GO:0065004 | protein-DNA complex assembly | 22/511 | 248/18670 | 1.40E-06 |
| BP | GO:0031638 | zymogen activation | 10/511 | 53/18670 | 1.46E-06 |
| BP | GO:0045840 | positive regulation of mitotic nuclear division | 10/511 | 53/18670 | 1.46E-06 |
| BP | GO:0031570 | DNA integrity checkpoint | 17/511 | 157/18670 | 1.49E-06 |
| BP | GO:0006336 | DNA replication-independent nucleosome assembly | 10/511 | 54/18670 | 1.75E-06 |
| BP | GO:0006260 | DNA replication | 23/511 | 273/18670 | 1.97E-06 |
| BP | GO:0034724 | DNA replication-independent nucleosome organization | 10/511 | 55/18670 | 2.09E-06 |
| BP | GO:0034080 | CENP-A containing nucleosome assembly | 9/511 | 44/18670 | 2.43E-06 |
| BP | GO:0061641 | CENP-A containing chromatin organization | 9/511 | 44/18670 | 2.43E-06 |
| BP | GO:0031497 | chromatin assembly | 19/511 | 202/18670 | 3.09E-06 |
| BP | GO:0034728 | nucleosome organization | 18/511 | 184/18670 | 3.21E-06 |
| BP | GO:0051231 | spindle elongation | 5/511 | 10/18670 | 3.39E-06 |
| BP | GO:0051781 | positive regulation of cell division | 12/511 | 87/18670 | 4.27E-06 |
| BP | GO:0071824 | protein-DNA complex subunit organization | 23/511 | 288/18670 | 4.84E-06 |
| BP | GO:0031055 | chromatin remodeling at centromere | 9/511 | 48/18670 | 5.21E-06 |
| BP | GO:0097529 | myeloid leukocyte migration | 19/511 | 210/18670 | 5.46E-06 |
| BP | GO:0006959 | humoral immune response | 26/511 | 356/18670 | 6.04E-06 |
| BP | GO:0016485 | protein processing | 19/511 | 217/18670 | 8.78E-06 |
| BP | GO:0051988 | regulation of attachment of spindle microtubules to kinetochore | 5/511 | 12/18670 | 1.02E-05 |
| BP | GO:0051785 | positive regulation of nuclear division | 10/511 | 66/18670 | 1.15E-05 |
| BP | GO:0051384 | response to glucocorticoid | 15/511 | 146/18670 | 1.17E-05 |
| BP | GO:0031145 | anaphase-promoting complex-dependent catabolic process | 11/511 | 81/18670 | 1.25E-05 |
| BP | GO:0044818 | mitotic G2/M transition checkpoint | 7/511 | 30/18670 | 1.30E-05 |
| BP | GO:0042403 | thyroid hormone metabolic process | 6/511 | 21/18670 | 1.56E-05 |
| BP | GO:0034502 | protein localization to chromosome | 11/511 | 83/18670 | 1.58E-05 |
| BP | GO:0051315 | attachment of mitotic spindle microtubules to kinetochore | 5/511 | 13/18670 | 1.62E-05 |
| BP | GO:0007143 | female meiotic nuclear division | 7/511 | 31/18670 | 1.64E-05 |
| BP | GO:0031572 | G2 DNA damage checkpoint | 7/511 | 31/18670 | 1.64E-05 |
| BP | GO:0006333 | chromatin assembly or disassembly | 19/511 | 228/18670 | 1.77E-05 |
| BP | GO:0052548 | regulation of endopeptidase activity | 28/511 | 425/18670 | 1.83E-05 |
| BP | GO:0010951 | negative regulation of endopeptidase activity | 20/511 | 250/18670 | 1.95E-05 |
| BP | GO:0045861 | negative regulation of proteolysis | 25/511 | 358/18670 | 1.95E-05 |
| BP | GO:0010755 | regulation of plasminogen activation | 5/511 | 14/18670 | 2.46E-05 |
| BP | GO:0040001 | establishment of mitotic spindle localization | 7/511 | 33/18670 | 2.54E-05 |
| BP | GO:0044843 | cell cycle G1/S phase transition | 22/511 | 298/18670 | 2.64E-05 |
| BP | GO:0061844 | antimicrobial humoral immune response mediated by antimicrobial peptide | 10/511 | 73/18670 | 2.86E-05 |
| BP | GO:1902750 | negative regulation of cell cycle G2/M phase transition | 12/511 | 105/18670 | 3.01E-05 |
| BP | GO:0032465 | regulation of cytokinesis | 11/511 | 89/18670 | 3.08E-05 |
| BP | GO:0044774 | mitotic DNA integrity checkpoint | 12/511 | 106/18670 | 3.31E-05 |
| BP | GO:1901989 | positive regulation of cell cycle phase transition | 12/511 | 106/18670 | 3.31E-05 |
| BP | GO:0045132 | meiotic chromosome segregation | 11/511 | 90/18670 | 3.43E-05 |
| BP | GO:0043486 | histone exchange | 9/511 | 60/18670 | 3.43E-05 |
| BP | GO:1901992 | positive regulation of mitotic cell cycle phase transition | 11/511 | 91/18670 | 3.80E-05 |
| BP | GO:0010466 | negative regulation of peptidase activity | 20/511 | 262/18670 | 3.81E-05 |
| BP | GO:0031960 | response to corticosteroid | 15/511 | 162/18670 | 4.02E-05 |
| BP | GO:0051653 | spindle localization | 8/511 | 48/18670 | 4.29E-05 |
| BP | GO:0031639 | plasminogen activation | 6/511 | 25/18670 | 4.64E-05 |
| BP | GO:0007062 | sister chromatid cohesion | 9/511 | 63/18670 | 5.11E-05 |
| BP | GO:0000076 | DNA replication checkpoint | 5/511 | 16/18670 | 5.12E-05 |
| BP | GO:0051382 | kinetochore assembly | 5/511 | 16/18670 | 5.12E-05 |
| BP | GO:0052547 | regulation of peptidase activity | 28/511 | 452/18670 | 5.47E-05 |
| BP | GO:0061351 | neural precursor cell proliferation | 14/511 | 150/18670 | 6.61E-05 |
| BP | GO:0006590 | thyroid hormone generation | 5/511 | 17/18670 | 7.10E-05 |
| BP | GO:0032506 | cytokinetic process | 7/511 | 39/18670 | 7.94E-05 |
| BP | GO:0030198 | extracellular matrix organization | 24/511 | 368/18670 | 8.42E-05 |
| BP | GO:0043062 | extracellular structure organization | 24/511 | 369/18670 | 8.78E-05 |
| BP | GO:0032467 | positive regulation of cytokinesis | 7/511 | 40/18670 | 9.40E-05 |
| BP | GO:0051782 | negative regulation of cell division | 5/511 | 18/18670 | 9.60E-05 |
| BP | GO:0051255 | spindle midzone assembly | 4/511 | 10/18670 | 0.000102 |
| BP | GO:0031099 | regeneration | 16/511 | 198/18670 | 0.000116 |
| BP | GO:0051047 | positive regulation of secretion | 26/511 | 428/18670 | 0.000137 |
| BP | GO:0051293 | establishment of spindle localization | 7/511 | 43/18670 | 0.000151 |
| BP | GO:0030595 | leukocyte chemotaxis | 17/511 | 224/18670 | 0.000154 |
| BP | GO:0035404 | histone-serine phosphorylation | 4/511 | 11/18670 | 0.000157 |
| BP | GO:0032602 | chemokine production | 10/511 | 89/18670 | 0.000158 |
| BP | GO:0006310 | DNA recombination | 20/511 | 292/18670 | 0.000169 |
| BP | GO:0006302 | double-strand break repair | 18/511 | 248/18670 | 0.000174 |
| BP | GO:0051346 | negative regulation of hydrolase activity | 27/511 | 463/18670 | 0.0002 |
| BP | GO:0006575 | cellular modified amino acid metabolic process | 15/511 | 188/18670 | 0.000215 |
| BP | GO:0010639 | negative regulation of organelle organization | 24/511 | 393/18670 | 0.000228 |
| BP | GO:0010972 | negative regulation of G2/M transition of mitotic cell cycle | 10/511 | 93/18670 | 0.000228 |
| BP | GO:0007077 | mitotic nuclear envelope disassembly | 4/511 | 12/18670 | 0.00023 |
| BP | GO:1904668 | positive regulation of ubiquitin protein ligase activity | 4/511 | 12/18670 | 0.00023 |
| BP | GO:0010955 | negative regulation of protein processing | 6/511 | 33/18670 | 0.000241 |
| BP | GO:1903318 | negative regulation of protein maturation | 6/511 | 33/18670 | 0.000241 |
| BP | GO:0007405 | neuroblast proliferation | 8/511 | 61/18670 | 0.000245 |
| BP | GO:0042445 | hormone metabolic process | 17/511 | 233/18670 | 0.000246 |
| BP | GO:0000082 | G1/S transition of mitotic cell cycle | 19/511 | 279/18670 | 0.000263 |
| BP | GO:0006261 | DNA-dependent DNA replication | 13/511 | 151/18670 | 0.000268 |
| BP | GO:1904666 | regulation of ubiquitin protein ligase activity | 5/511 | 22/18670 | 0.00027 |
| BP | GO:0042035 | regulation of cytokine biosynthetic process | 11/511 | 114/18670 | 0.000294 |
| BP | GO:0051656 | establishment of organelle localization | 24/511 | 401/18670 | 0.000306 |
| BP | GO:0007064 | mitotic sister chromatid cohesion | 5/511 | 23/18670 | 0.000337 |
| BP | GO:0045109 | intermediate filament organization | 5/511 | 23/18670 | 0.000337 |
| BP | GO:1903429 | regulation of cell maturation | 5/511 | 23/18670 | 0.000337 |
| BP | GO:0051604 | protein maturation | 19/511 | 285/18670 | 0.000343 |
| BP | GO:0045104 | intermediate filament cytoskeleton organization | 7/511 | 50/18670 | 0.000398 |
| BP | GO:0070613 | regulation of protein processing | 8/511 | 66/18670 | 0.000425 |
| BP | GO:0061982 | meiosis I cell cycle process | 11/511 | 119/18670 | 0.000427 |
| BP | GO:0007100 | mitotic centrosome separation | 4/511 | 14/18670 | 0.000446 |
| BP | GO:0050930 | induction of positive chemotaxis | 4/511 | 14/18670 | 0.000446 |
| BP | GO:0090231 | regulation of spindle checkpoint | 4/511 | 14/18670 | 0.000446 |
| BP | GO:0090266 | regulation of mitotic cell cycle spindle assembly checkpoint | 4/511 | 14/18670 | 0.000446 |
| BP | GO:1903504 | regulation of mitotic spindle checkpoint | 4/511 | 14/18670 | 0.000446 |
| BP | GO:0045103 | intermediate filament-based process | 7/511 | 51/18670 | 0.000451 |
| BP | GO:0006270 | DNA replication initiation | 6/511 | 37/18670 | 0.000461 |
| BP | GO:0000079 | regulation of cyclin-dependent protein serine/threonine kinase activity | 10/511 | 102/18670 | 0.000484 |
| BP | GO:0051307 | meiotic chromosome separation | 5/511 | 25/18670 | 0.000508 |
| BP | GO:1903317 | regulation of protein maturation | 8/511 | 68/18670 | 0.000521 |
| BP | GO:0042089 | cytokine biosynthetic process | 11/511 | 123/18670 | 0.000566 |
| BP | GO:0051299 | centrosome separation | 4/511 | 15/18670 | 0.000595 |
| BP | GO:0007098 | centrosome cycle | 11/511 | 124/18670 | 0.000606 |
| BP | GO:0042107 | cytokine metabolic process | 11/511 | 124/18670 | 0.000606 |
| BP | GO:0042044 | fluid transport | 5/511 | 26/18670 | 0.000615 |
| BP | GO:0030199 | collagen fibril organization | 7/511 | 54/18670 | 0.000642 |
| BP | GO:0000077 | DNA damage checkpoint | 12/511 | 145/18670 | 0.000651 |
| BP | GO:1904029 | regulation of cyclin-dependent protein kinase activity | 10/511 | 106/18670 | 0.000657 |
| BP | GO:0043044 | ATP-dependent chromatin remodeling | 9/511 | 88/18670 | 0.000675 |
| BP | GO:0030397 | membrane disassembly | 4/511 | 16/18670 | 0.000777 |
| BP | GO:0051081 | nuclear envelope disassembly | 4/511 | 16/18670 | 0.000777 |
| BP | GO:1903532 | positive regulation of secretion by cell | 23/511 | 403/18670 | 0.00078 |
| BP | GO:1903035 | negative regulation of response to wounding | 9/511 | 90/18670 | 0.000795 |
| BP | GO:0072401 | signal transduction involved in DNA integrity checkpoint | 8/511 | 73/18670 | 0.000842 |
| BP | GO:0072422 | signal transduction involved in DNA damage checkpoint | 8/511 | 73/18670 | 0.000842 |
| BP | GO:0007565 | female pregnancy | 14/511 | 192/18670 | 0.000851 |
| BP | GO:0051984 | positive regulation of chromosome segregation | 5/511 | 28/18670 | 0.000879 |
| BP | GO:0007018 | microtubule-based movement | 18/511 | 285/18670 | 0.000915 |
| BP | GO:0072395 | signal transduction involved in cell cycle checkpoint | 8/511 | 74/18670 | 0.000922 |
| BP | GO:0071385 | cellular response to glucocorticoid stimulus | 7/511 | 58/18670 | 0.000993 |
| BP | GO:0007076 | mitotic chromosome condensation | 4/511 | 17/18670 | 0.000994 |
| BP | GO:0042303 | molting cycle | 10/511 | 112/18670 | 0.001009 |
| BP | GO:0042633 | hair cycle | 10/511 | 112/18670 | 0.001009 |
| BP | GO:0000083 | regulation of transcription involved in G1/S transition of mitotic cell cycle | 5/511 | 29/18670 | 0.001038 |
| BP | GO:0000132 | establishment of mitotic spindle orientation | 5/511 | 29/18670 | 0.001038 |
| BP | GO:0031023 | microtubule organizing center organization | 11/511 | 133/18670 | 0.001086 |
| BP | GO:0042770 | signal transduction in response to DNA damage | 11/511 | 133/18670 | 0.001086 |
| BP | GO:0000724 | double-strand break repair via homologous recombination | 11/511 | 134/18670 | 0.001154 |
| BP | GO:2000779 | regulation of double-strand break repair | 8/511 | 77/18670 | 0.001198 |
| BP | GO:0071549 | cellular response to dexamethasone stimulus | 5/511 | 30/18670 | 0.001218 |
| BP | GO:0044706 | multi-multicellular organism process | 15/511 | 222/18670 | 0.001226 |
| BP | GO:0000725 | recombinational repair | 11/511 | 135/18670 | 0.001227 |
| BP | GO:2000243 | positive regulation of reproductive process | 8/511 | 78/18670 | 0.001304 |
| BP | GO:0071384 | cellular response to corticosteroid stimulus | 7/511 | 61/18670 | 0.001343 |
| BP | GO:0035987 | endodermal cell differentiation | 6/511 | 45/18670 | 0.001344 |
| BP | GO:0044773 | mitotic DNA damage checkpoint | 9/511 | 97/18670 | 0.00136 |
| BP | GO:0006691 | leukotriene metabolic process | 5/511 | 31/18670 | 0.00142 |
| BP | GO:0045766 | positive regulation of angiogenesis | 14/511 | 204/18670 | 0.001522 |
| BP | GO:2000241 | regulation of reproductive process | 12/511 | 160/18670 | 0.001537 |
| BP | GO:0006833 | water transport | 4/511 | 19/18670 | 0.00155 |
| BP | GO:0050715 | positive regulation of cytokine secretion | 11/511 | 139/18670 | 0.001553 |
| BP | GO:0032392 | DNA geometric change | 10/511 | 119/18670 | 0.001602 |
| BP | GO:1904018 | positive regulation of vasculature development | 15/511 | 230/18670 | 0.00174 |
| BP | GO:0032642 | regulation of chemokine production | 8/511 | 82/18670 | 0.001803 |
| BP | GO:0070507 | regulation of microtubule cytoskeleton organization | 13/511 | 186/18670 | 0.001883 |
| BP | GO:0060326 | cell chemotaxis | 18/511 | 304/18670 | 0.001884 |
| BP | GO:0048608 | reproductive structure development | 23/511 | 431/18670 | 0.001886 |
| BP | GO:0007095 | mitotic G2 DNA damage checkpoint | 4/511 | 20/18670 | 0.001896 |
| BP | GO:0034104 | negative regulation of tissue remodeling | 4/511 | 20/18670 | 0.001896 |
| BP | GO:0051443 | positive regulation of ubiquitin-protein transferase activity | 5/511 | 33/18670 | 0.001897 |
| BP | GO:1902692 | regulation of neuroblast proliferation | 5/511 | 33/18670 | 0.001897 |
| BP | GO:0061458 | reproductive system development | 23/511 | 434/18670 | 0.00206 |
| BP | GO:0051445 | regulation of meiotic cell cycle | 6/511 | 49/18670 | 0.002104 |
| BP | GO:0071715 | icosanoid transport | 6/511 | 49/18670 | 0.002104 |
| BP | GO:1901571 | fatty acid derivative transport | 6/511 | 49/18670 | 0.002104 |
| BP | GO:0033314 | mitotic DNA replication checkpoint | 3/511 | 10/18670 | 0.002119 |
| BP | GO:1902101 | positive regulation of metaphase/anaphase transition of cell cycle | 3/511 | 10/18670 | 0.002119 |
| BP | GO:0030593 | neutrophil chemotaxis | 9/511 | 104/18670 | 0.002211 |
| BP | GO:0031935 | regulation of chromatin silencing | 4/511 | 21/18670 | 0.002292 |
| BP | GO:0001706 | endoderm formation | 6/511 | 50/18670 | 0.002337 |
| BP | GO:0001942 | hair follicle development | 8/511 | 86/18670 | 0.002441 |
| BP | GO:0051294 | establishment of spindle orientation | 5/511 | 35/18670 | 0.002481 |
| BP | GO:0010812 | negative regulation of cell-substrate adhesion | 7/511 | 68/18670 | 0.002533 |
| BP | GO:0010876 | lipid localization | 22/511 | 415/18670 | 0.002542 |
| BP | GO:0002793 | positive regulation of peptide secretion | 17/511 | 288/18670 | 0.002569 |
| BP | GO:0048146 | positive regulation of fibroblast proliferation | 6/511 | 51/18670 | 0.002589 |
| BP | GO:0030330 | DNA damage response, signal transduction by p53 class mediator | 9/511 | 107/18670 | 0.002686 |
| BP | GO:0002052 | positive regulation of neuroblast proliferation | 4/511 | 22/18670 | 0.002741 |
| BP | GO:0002385 | mucosal immune response | 5/511 | 36/18670 | 0.002817 |
| BP | GO:0022404 | molting cycle process | 8/511 | 88/18670 | 0.002821 |
| BP | GO:0022405 | hair cycle process | 8/511 | 88/18670 | 0.002821 |
| BP | GO:0098773 | skin epidermis development | 8/511 | 88/18670 | 0.002821 |
| BP | GO:2000177 | regulation of neural precursor cell proliferation | 8/511 | 88/18670 | 0.002821 |
| BP | GO:0000727 | double-strand break repair via break-induced replication | 3/511 | 11/18670 | 0.002854 |
| BP | GO:0040015 | negative regulation of multicellular organism growth | 3/511 | 11/18670 | 0.002854 |
| BP | GO:1905820 | positive regulation of chromosome separation | 3/511 | 11/18670 | 0.002854 |
| BP | GO:0051438 | regulation of ubiquitin-protein transferase activity | 6/511 | 52/18670 | 0.00286 |
| BP | GO:0055078 | sodium ion homeostasis | 6/511 | 52/18670 | 0.00286 |
| BP | GO:0072331 | signal transduction by p53 class mediator | 16/511 | 267/18670 | 0.002925 |
| BP | GO:0050714 | positive regulation of protein secretion | 16/511 | 268/18670 | 0.003034 |
| BP | GO:0001890 | placenta development | 11/511 | 152/18670 | 0.003139 |
| BP | GO:0007131 | reciprocal meiotic recombination | 6/511 | 53/18670 | 0.003152 |
| BP | GO:0032508 | DNA duplex unwinding | 9/511 | 110/18670 | 0.003238 |
| BP | GO:0035825 | homologous recombination | 6/511 | 54/18670 | 0.003466 |
| BP | GO:0001578 | microtubule bundle formation | 8/511 | 91/18670 | 0.003475 |
| BP | GO:0070268 | cornification | 9/511 | 112/18670 | 0.003653 |
| BP | GO:0045926 | negative regulation of growth | 15/511 | 249/18670 | 0.003712 |
| BP | GO:0008228 | opsonization | 3/511 | 12/18670 | 0.003729 |
| BP | GO:0019755 | one-carbon compound transport | 3/511 | 12/18670 | 0.003729 |
| BP | GO:0070601 | centromeric sister chromatid cohesion | 3/511 | 12/18670 | 0.003729 |
| BP | GO:0072697 | protein localization to cell cortex | 3/511 | 12/18670 | 0.003729 |
| BP | GO:0002227 | innate immune response in mucosa | 4/511 | 24/18670 | 0.003814 |
| BP | GO:0044062 | regulation of excretion | 4/511 | 24/18670 | 0.003814 |
| BP | GO:0046697 | decidualization | 4/511 | 24/18670 | 0.003814 |
| CC | GO:0000779 | condensed chromosome, centromeric region | 30/532 | 118/19717 | 4.01E-21 |
| CC | GO:0000793 | condensed chromosome | 39/532 | 223/19717 | 9.25E-21 |
| CC | GO:0000775 | chromosome, centromeric region | 36/532 | 193/19717 | 3.20E-20 |
| CC | GO:0000776 | kinetochore | 30/532 | 135/19717 | 2.58E-19 |
| CC | GO:0000777 | condensed chromosome kinetochore | 27/532 | 105/19717 | 2.81E-19 |
| CC | GO:0098687 | chromosomal region | 43/532 | 347/19717 | 6.43E-17 |
| CC | GO:0005819 | spindle | 42/532 | 347/19717 | 3.51E-16 |
| CC | GO:0042599 | lamellar body | 10/532 | 17/19717 | 3.09E-12 |
| CC | GO:0072686 | mitotic spindle | 20/532 | 109/19717 | 1.14E-11 |
| CC | GO:0000780 | condensed nuclear chromosome, centromeric region | 11/532 | 26/19717 | 2.67E-11 |
| CC | GO:0030496 | midbody | 23/532 | 173/19717 | 2.98E-10 |
| CC | GO:0005876 | spindle microtubule | 14/532 | 59/19717 | 3.99E-10 |
| CC | GO:0000940 | condensed chromosome outer kinetochore | 8/532 | 14/19717 | 6.94E-10 |
| CC | GO:0000794 | condensed nuclear chromosome | 17/532 | 99/19717 | 1.18E-09 |
| CC | GO:0000778 | condensed nuclear chromosome kinetochore | 8/532 | 15/19717 | 1.45E-09 |
| CC | GO:0000922 | spindle pole | 20/532 | 164/19717 | 1.95E-08 |
| CC | GO:0005871 | kinesin complex | 11/532 | 55/19717 | 2.03E-07 |
| CC | GO:0051233 | spindle midzone | 9/532 | 34/19717 | 2.04E-07 |
| CC | GO:0005874 | microtubule | 31/532 | 416/19717 | 3.77E-07 |
| CC | GO:0044815 | DNA packaging complex | 14/532 | 115/19717 | 2.68E-06 |
| CC | GO:0005875 | microtubule associated complex | 15/532 | 152/19717 | 1.61E-05 |
| CC | GO:0062023 | collagen-containing extracellular matrix | 27/532 | 406/19717 | 1.73E-05 |
| CC | GO:0000786 | nucleosome | 12/532 | 107/19717 | 3.18E-05 |
| CC | GO:0016324 | apical plasma membrane | 22/532 | 318/19717 | 5.74E-05 |
| CC | GO:0031225 | anchored component of membrane | 15/532 | 170/19717 | 6.00E-05 |
| CC | GO:0005771 | multivesicular body | 8/532 | 51/19717 | 6.11E-05 |
| CC | GO:0032993 | protein-DNA complex | 16/532 | 202/19717 | 0.000125 |
| CC | GO:0045177 | apical part of cell | 24/532 | 384/19717 | 0.000131 |
| CC | GO:0032153 | cell division site | 8/532 | 68/19717 | 0.000475 |
| CC | GO:0045120 | pronucleus | 4/532 | 15/19717 | 0.000564 |
| CC | GO:0046658 | anchored component of plasma membrane | 7/532 | 59/19717 | 0.001014 |
| CC | GO:0045334 | clathrin-coated endocytic vesicle | 7/532 | 63/19717 | 0.0015 |
| CC | GO:0005680 | anaphase-promoting complex | 4/532 | 21/19717 | 0.002177 |
| CC | GO:0032154 | cleavage furrow | 6/532 | 55/19717 | 0.003548 |
| CC | GO:0000800 | lateral element | 3/532 | 13/19717 | 0.004565 |
| CC | GO:1990023 | mitotic spindle midzone | 3/532 | 13/19717 | 0.004565 |
| CC | GO:0045171 | intercellular bridge | 6/532 | 59/19717 | 0.00504 |
| CC | GO:0030136 | clathrin-coated vesicle | 12/532 | 188/19717 | 0.005147 |
| CC | GO:0097431 | mitotic spindle pole | 4/532 | 27/19717 | 0.005618 |
| CC | GO:0010369 | chromocenter | 3/532 | 14/19717 | 0.005695 |
| CC | GO:0000152 | nuclear ubiquitin ligase complex | 5/532 | 43/19717 | 0.005801 |
| MF | GO:0004867 | serine-type endopeptidase inhibitor activity | 16/507 | 94/17696 | 9.54E-09 |
| MF | GO:0008017 | microtubule binding | 24/507 | 246/17696 | 1.82E-07 |
| MF | GO:0015631 | tubulin binding | 28/507 | 336/17696 | 4.72E-07 |
| MF | GO:0004866 | endopeptidase inhibitor activity | 18/507 | 175/17696 | 2.94E-06 |
| MF | GO:0035173 | histone kinase activity | 6/507 | 17/17696 | 5.08E-06 |
| MF | GO:0030414 | peptidase inhibitor activity | 18/507 | 182/17696 | 5.14E-06 |
| MF | GO:0061135 | endopeptidase regulator activity | 18/507 | 182/17696 | 5.14E-06 |
| MF | GO:0061134 | peptidase regulator activity | 19/507 | 219/17696 | 1.88E-05 |
| MF | GO:0003777 | microtubule motor activity | 11/507 | 84/17696 | 2.69E-05 |
| MF | GO:0008201 | heparin binding | 15/507 | 169/17696 | 0.000108 |
| MF | GO:0005539 | glycosaminoglycan binding | 18/507 | 229/17696 | 0.000112 |
| MF | GO:0071723 | lipopeptide binding | 4/507 | 10/17696 | 0.000122 |
| MF | GO:0004252 | serine-type endopeptidase activity | 14/507 | 160/17696 | 0.000212 |
| MF | GO:0016825 | hydrolase activity, acting on acid phosphorus-nitrogen bonds | 15/507 | 186/17696 | 0.000311 |
| MF | GO:0017171 | serine hydrolase activity | 15/507 | 186/17696 | 0.000311 |
| MF | GO:0016887 | ATPase activity | 26/507 | 434/17696 | 0.000344 |
| MF | GO:0005172 | vascular endothelial growth factor receptor binding | 4/507 | 13/17696 | 0.000387 |
| MF | GO:0003688 | DNA replication origin binding | 5/507 | 24/17696 | 0.000512 |
| MF | GO:0003774 | motor activity | 12/507 | 136/17696 | 0.000546 |
| MF | GO:0005044 | scavenger receptor activity | 7/507 | 51/17696 | 0.000591 |
| MF | GO:0008236 | serine-type peptidase activity | 14/507 | 182/17696 | 0.00078 |
| MF | GO:0004857 | enzyme inhibitor activity | 22/507 | 375/17696 | 0.00126 |
| MF | GO:0038024 | cargo receptor activity | 8/507 | 76/17696 | 0.001468 |
| MF | GO:0048018 | receptor ligand activity | 26/507 | 482/17696 | 0.001597 |
| MF | GO:0030546 | signaling receptor activator activity | 26/507 | 487/17696 | 0.001843 |

Supplementary Table 9. KEGG pathway analyses of DEGs between the high-risk and low-risk groups.

| ID | Description | GeneRatio | BgRatio | pvalue |
| --- | --- | --- | --- | --- |
| hsa04110 | Cell cycle | 19/234 | 124/8076 | 2.22E-09 |
| hsa04914 | Progesterone-mediated oocyte maturation | 11/234 | 100/8076 | 0.000139 |
| hsa04115 | p53 signaling pathway | 9/234 | 73/8076 | 0.000238 |
| hsa04114 | Oocyte meiosis | 12/234 | 129/8076 | 0.000345 |
| hsa05322 | Systemic lupus erythematosus | 12/234 | 136/8076 | 0.00056 |
| hsa04610 | Complement and coagulation cascades | 9/234 | 85/8076 | 0.000748 |
| hsa00591 | Linoleic acid metabolism | 5/234 | 29/8076 | 0.001315 |

Supplementary Figure 1


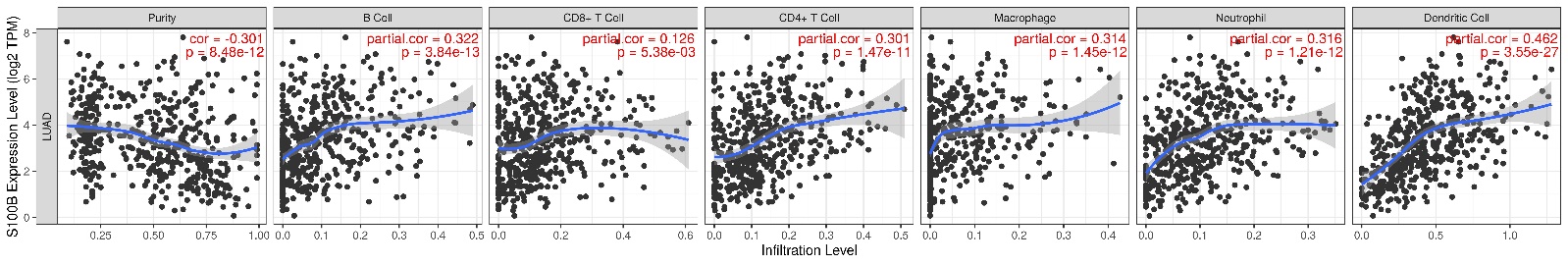

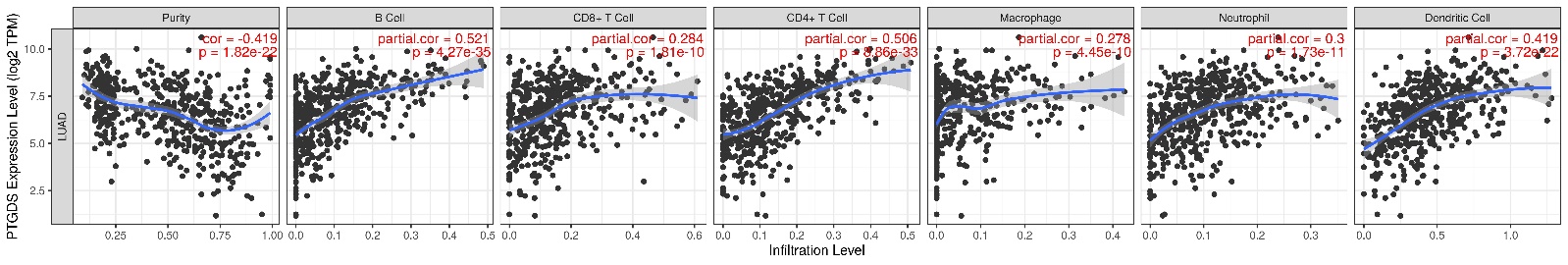

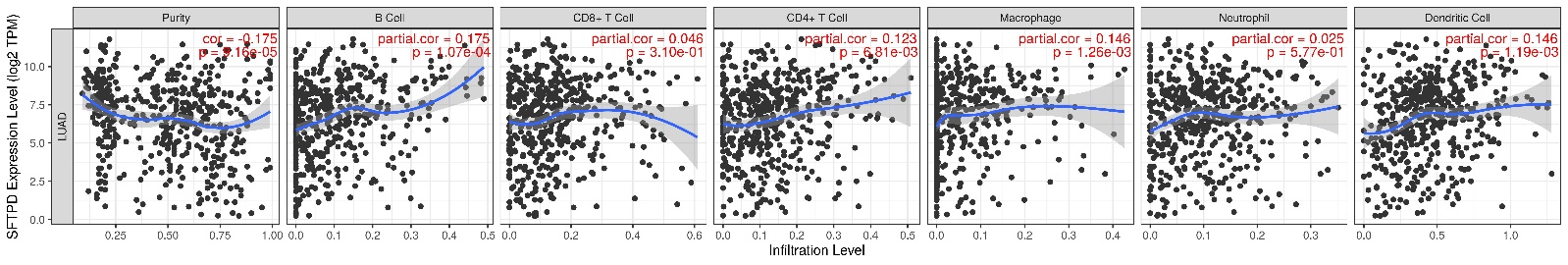

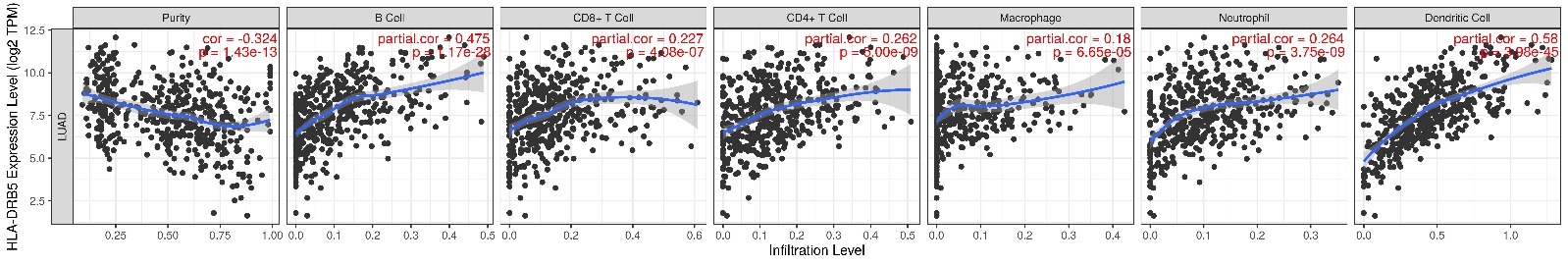

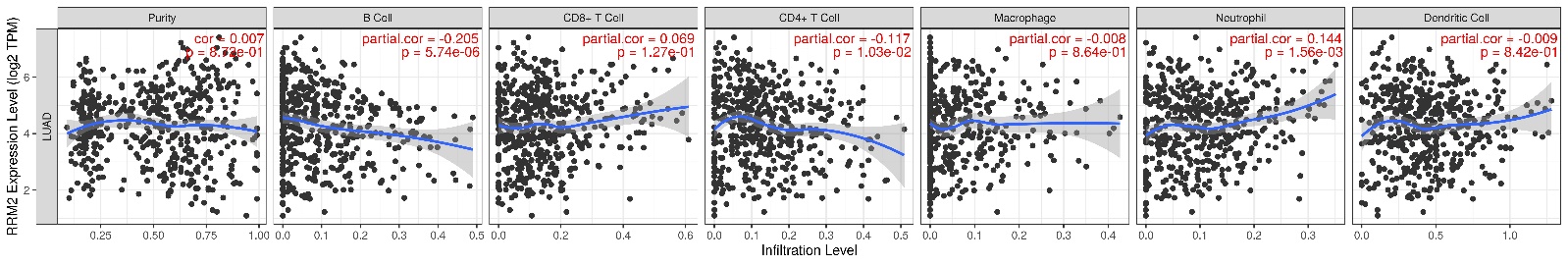

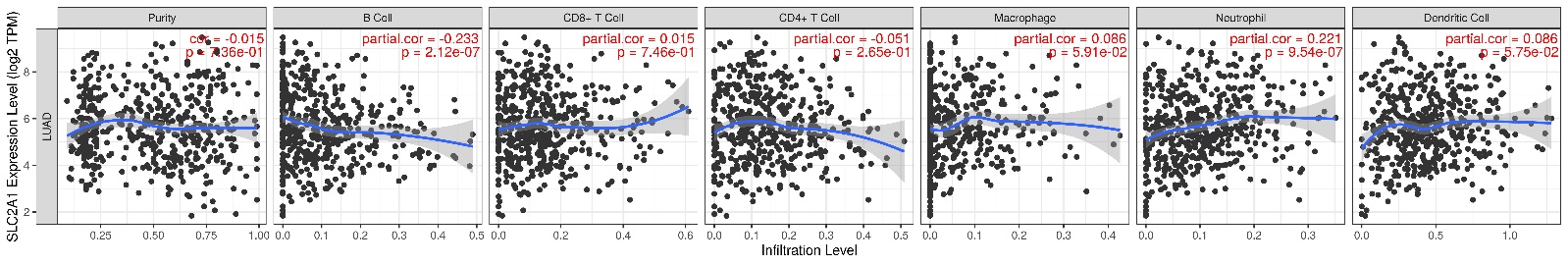

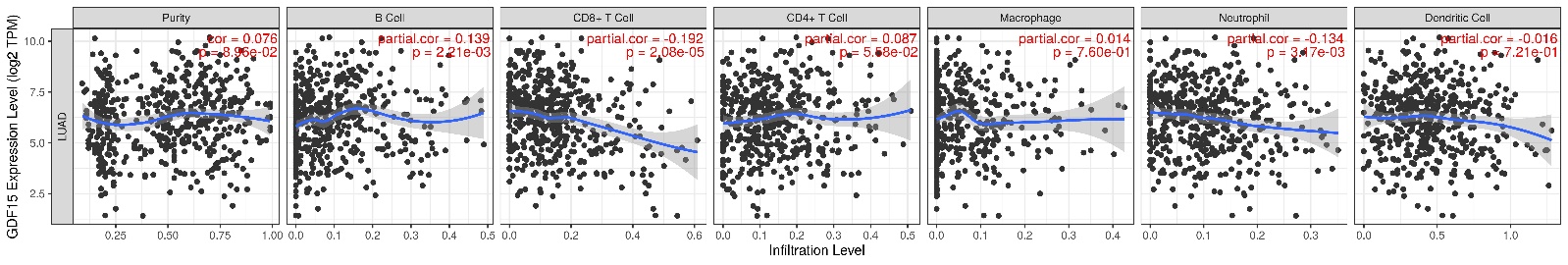

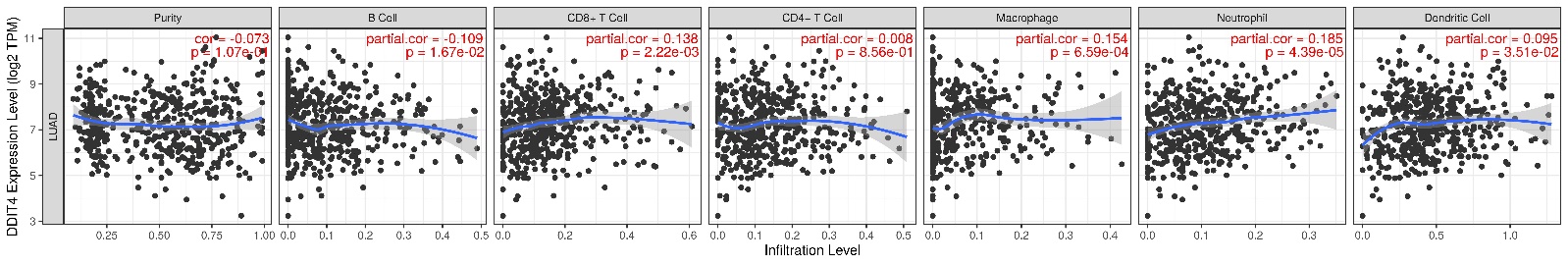

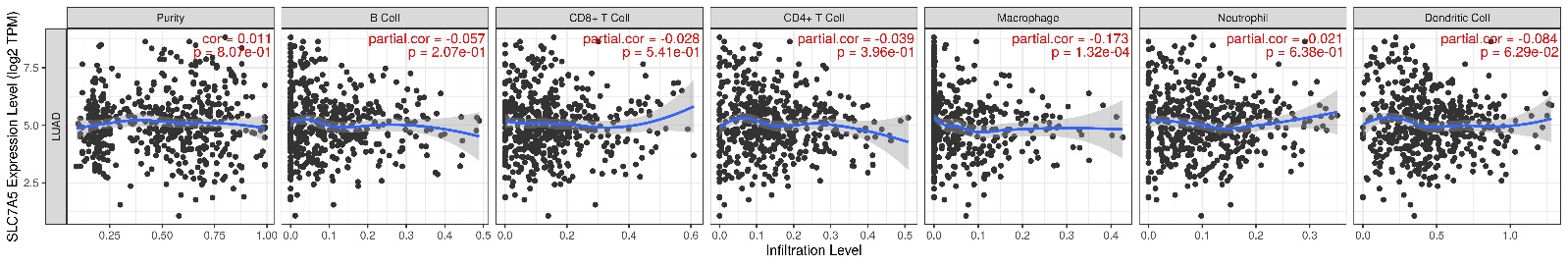

Supplement: Supplementary file 1 [file Data_Sheet_1.docx]
